# Structural modifications of toxyloxanthone C and macluraxanthone isolated from *Maclura cochinchinensis*: cytotoxicity, antibacterial activity, and *in silico* studies

**DOI:** 10.1039/d5ra05758b

**Published:** 2025-09-17

**Authors:** Chaiwat Linphosan, Waranya Klangsawad, Jantana Yahuafai, Jinda Jandaruang, Trinop Promgool, Siripit Pitchuanchom, Jakkapat Paluka, Sophon Boonlue, Kitisak Poopasit, Kwanjai Kanokmedhakul, Oue-artorn Limtragool

**Affiliations:** a Multidisciplinary Research Unit of Pure and Applied Chemistry, Department of Chemistry and Center of Excellence for Innovation in Chemistry, Faculty of Science, Mahasarakham University Maha Sarakham Thailand oueartorn.l@msu.ac.th +66-43 754246 +66-43 754246; b Department of Chemistry and Center of Excellence for Innovation in Chemistry, Faculty of Science, Khon Kaen University Khon Kaen Thailand; c Clinical Research Section, Division of Research and Academic Support, National Cancer Institute Bangkok Thailand; d Innovation in Chemistry for Community Research Unit, Chemistry Program, Faculty of Science and Technology, Sakon Nakhon Rajabhat University Sakon Nakhon Thailand; e Department of Industrial Chemistry, Faculty of Applied Science, King Mongkut's University of Technology North Bangkok Bangkok Thailand; f Research Administration Division, Khon Kaen University Thailand; g Department of Microbiology, Faculty of Science, Khon Kaen University Khon Kaen Thailand

## Abstract

Toxyloxanthone C and macluraxanthone, isolated from the roots of *Maclura cochinchinensis*, have been reported to exhibit promising cytotoxic and antibacterial activities. Accordingly, thirteen xanthone derivatives were synthesized from these two parent xanthones by simple acylation, alkylation, sulfonylation, and bromination reactions. All derivatives were evaluated for their cytotoxicity against three cancer cell lines, HelaS3, A549, and HepG2, and their antibacterial activity against four Gram-positive bacterial strains, namely methicillin-resistant *Staphylococcus aureus*, *S. aureus*, *Bacillus subtilis*, and *Bacillus cereus*. 5,6-Diacetoxytoxyloxanthone C (1a) displayed cytotoxicity against three cancer cells with IC_50_ values ranging from 12.20 to 22.61 μM. Additionally, 1a demonstrated potent cytotoxicity against A549 cells with IC_50_ = 12.20 μM, without showing cytotoxicity toward Vero cells. Moreover, 1,5,6-tripentanoyloxytoxyloxanthone C (1b) and 4-bromotoxyloxanthone C (1f) exhibited significant cytotoxicity against A549 cells with IC_50_ values of 5.77 and 7.52 μM, respectively. In addition, 1a and 1f showed potent antibacterial activity against all bacteria tested with the same MIC value of 4 μg mL^−1^, which was stronger than the parent xanthone 1. Molecular docking studies revealed that 1a, 1b, and 1f interacted with CDK2 through a competitive inhibition mechanism. Additionally, the binding conformations of 1a and 1f within the active sites of key enzymes involved in bacterial cell wall synthesis were similar to that of the tetracycline drug. The *in silico* physicochemical investigation indicated that 1a and 1f exhibited a favorable drug-likeness. Based on this finding, 1a represents a promising lead candidate for further study as an anticancer and an antibacterial agent.

## Abstract 2

Thirteen xanthone derivatives were synthesized through the structural modification of toxyloxanthone C (1) and macluraxanthone (2). The acetylated and brominated derivatives of 1 exhibited enhanced anticancer and antibacterial activities.

## Graphical abstract

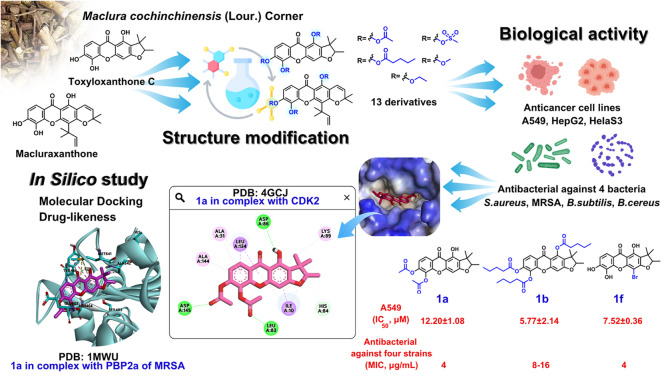

## 1 Introduction

*Maclura cochinchinensis* (Lour.) Corner is a shrub belonging to the Moraceae family and is widely distributed across various regions of Asia, including China, Japan, Korea, Taiwan, India, Vietnam, Laos, and Thailand. Traditionally, the heartwood of this plant has been utilized in the treatment of chronic fever, diarrhea, and skin infections.^1,2^ Additionally, its leaves have been used for wound healing, while the roots have been employed in traditional medicine to treat bruising, boils, scabies, rheumatism, blood stasis, dysmenorrhea, and contusions.^2,3^ Xanthones and flavonoids, the major bioactive constituents isolated from *M. cochinchinensis*,^2–7^ have demonstrated a variety of biological activities, including antioxidant,^1^ antibacterial,^4,7^ anticancer,^1,3,4^ and anti-inflammatory.^2,6^

Several xanthone derivatives have been reported for structural modifications to enhance their biological activity. Three xanthones, α-mangostin, β-mangostin, and γ-mangostin, were partially modified under acidic conditions and subsequently evaluated for their antibacterial activities. Several of their analogues demonstrated improved pharmacokinetic properties. However, all modified derivatives showed weaker antibacterial activities against methicillin-resistant *Staphylococcus aureus* (MRSA), *Bacillus subtilis*, and *Pseudomonas aeruginosa* compared to their parent compounds.^8^ Furthermore, α-mangostin was partially synthesized by cationic modification of the free 3-OH and 6-OH groups with amine moieties, followed by antibacterial evaluation. Some of its derivatives exhibited enhanced antibacterial potency against MRSA and *S. aureus*.^9^ Ananixanthone isolated from *Calophyllum teysmannii* stem bark, underwent structural modifications through acetylation, methylation, and benzylation at the 1-OH and 5-OH positions. The parent compound exhibited more potent cytotoxicity against SNU-1 (stomach cancer) and K562 (leukemia) cell lines. Among its derivatives, only 5-methoxyananixanthone exhibited superior cytotoxicity against LS174T (colon cancer) cells.^10^

Besides *M. cochinchinensis*, toxyloxanthone C (1) has been isolated from various plants such as *Cudrania*,^11–15^*Cratoxylum*,^16^ and *Rheedia*^17^ species. It has been reported to exhibit antifungal,^12,18^ antibacterial,^14,19^ and cytotoxicity against HCT-116, SMMC-7721, SGC-7901, and BGC-823 cancer cell lines.^13,18^ Whereas, macluraxanthone (2) has been reported in the *Garcinia*,^20–22^*Mesua*,^23^ and *Calophyllum*^24^ species. It has been shown cytotoxicity against several cancer cell lines such as HeLa, A549, PC-3, HT-29, WPMY-1, Hep G2, NCI–H23, and KB.^20,21,24,25^ Additionally, 2 exhibited *anti*-HIV,^20^ antibacterial,^4^ and antimalarial.^26^ From our previous report,^4^ xanthones 1 and 2 were isolated from *M. cochinchinensis*, they showed potent cytotoxicity toward three cancer cell lines, HelaS3, A549, and HepG2 and also exhibited antibacterial activity against four Gram-positive bacteria MRSA, *S. aureus*, *B. subtilis*, and *Bacillus cereus*. Herein, further study of these bioactive xanthones is reported. The chemical structural modifications of parent xanthones 1 and 2 using simple organic reactions yielded thirteen xanthone derivatives. All compounds were evaluated for their cytotoxicity and antibacterial activity. Additionally, molecular docking simulations and drug-like property are also presented.

## 2 Result and discussion

### 2.1 Chemistry

**Scheme 1:**
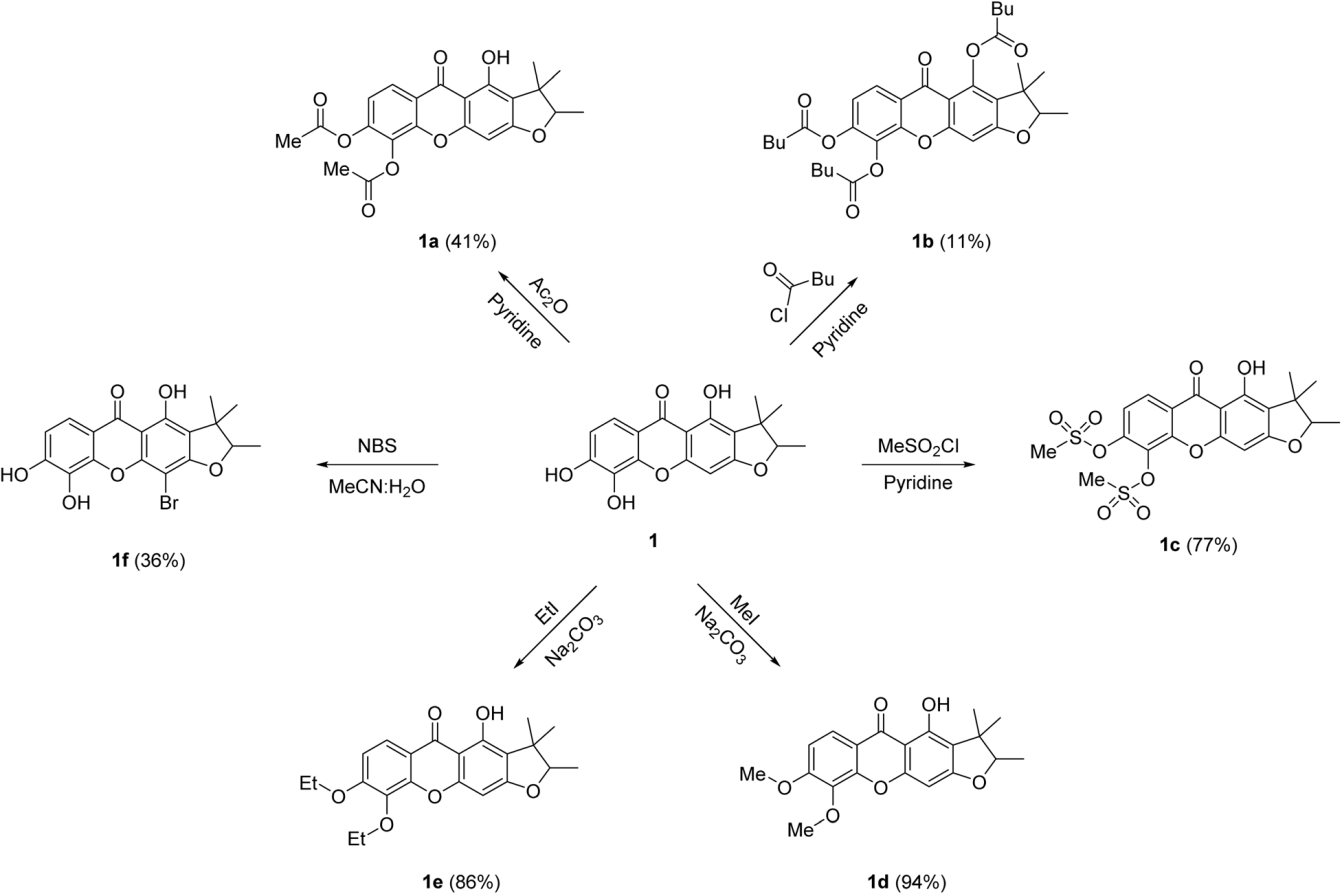
Derivatives of toxyloxanthone C (1).

**Scheme 2:**
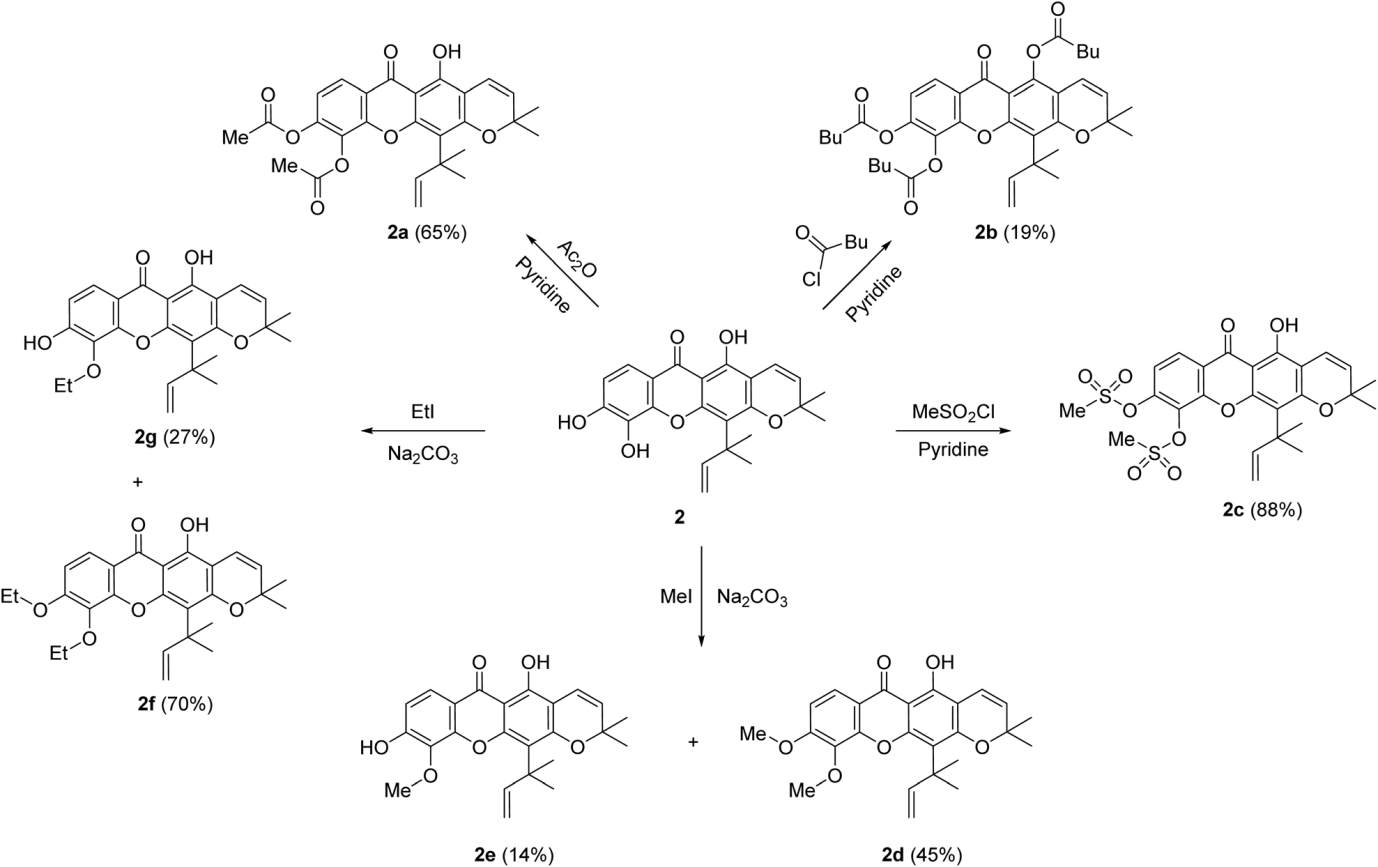
Derivatives of macluraxanthone (2).

From our previous reported,^4^ toxyloxanthone C (1) and macluraxanthone (2) were the main compounds isolated from the roots of *M. cochinchinensis*. Xanthone 1 exhibited cytotoxicity against the HelaS3, A549, HepG2, and Vero cell lines with IC_50_ values of 13.55 ± 1.11, 21.78 ± 4.89, 20.81 ± 2.57, and 8.52 ± 0.64 μM, respectively. While xanthone 2 showed cytotoxicity against the HelaS3, A549, HepG2, and Vero cell lines with IC_50_ values of 1.59 ± 0.12, 6.46 ± 0.98, 5.26 ± 0.41, and 4.29 ± 0.60, respectively. In addition, 1 displayed antibacterial activity against MRSA, *S. aureus, B. subtilis*, and *B. cereus* with MIC values of 128 μg mL^−1^ for all strains, and 2 exhibited activities toward MRSA, *B. subtilis*, and *B. cereus* with MIC values of 16, 64, and 64 μg mL^−1^, respectively.^4^ To improve their bioactivities, structural modifications of 1 and 2 were performed. Thirteen xanthone derivatives were successfully synthesized using simple organic reactions, including acylation, alkylation, sulfonylation, and bromination, as shown in Schemes 1 and 2.

According to literature,^27^ acetyl substitution at 6-OH of α-mangostin exhibited significant antibacterial activity against MRSA, which was better than the standard drug penicillin. Furthermore, the acetyl substitutions at 1-OH and 6-OH of α-mangostin, when combined with penicillin, exhibited greater antibacterial efficacy against MRSA compared to penicillin alone. Therefore, acylation reactions were applied to the parent xanthones 1 and 2 to investigate the antibacterial activity. Acetylation of 1 and 2 using acetic anhydride in pyridine afforded derivatives 1a (41%) and 2a (65%). The ^1^H NMR data of 1a showed two singlet signals of two methyl groups at *δ*_H_ 2.43 and 2.35, while the ^13^C NMR data of 1a showed carbonyl signals at *δ*_C_ 167.7 and 167.4, corresponding to acetoxy groups. Similarly, the ^1^H NMR data signals of 2a at *δ*_H_ 2.39 and 2.34, together with its ^13^C NMR signals at *δ*_C_ 167.7 and 167.6, confirmed the presence of two acetoxy groups. Additionally, the ^1^H NMR data of 1a and 2a exhibited intramolecular H-bonding signals at *δ*_H_ 12.84 and 13.40, respectively. Treatments of 1 and 2 with pentanoyl chloride in the presence of pyridine provided xanthone derivatives 1b (11%) and 2b (19%), respectively. Notably, the ^1^H NMR spectra of 1b and 2b displayed no intramolecular H-bonding signal around *δ*_H_ 12.8 and 13.4, respectively. In addition, their ^13^C NMR spectra revealed three carbonyl signals at *δ*_C_ 172.6, 170.6, and 170.1 for 1b and at *δ*_C_ 172.1, 170.6, and 170.5 for 2b. In the acylation reactions, the use of a more electrophilic acid chloride resulted in acyl substitution at the 1-OH, 5-OH, and 6-OH positions.^27^ Structural modifications were further carried out by treated 1 and 2 with methanesulfonyl chloride in pyridine, afforded mesylated derivatives 1c (77%) and 2c (88%). The ^1^H NMR spectra of 1c and 2c showed singlet signals of two methyl groups at *δ*_H_ 3.49 and 3.38 for 1c, as well as 3.43 and 3.33 for 2c, corresponding to mesyl groups. According to a previous report,^10^ methylation of 5-OH group in ananixanthone resulted in enhanced cytotoxicity against the LS174T cell line compared to its parent compound. Therefore, alkylation reactions were carried out on xanthones 1 and 2 to further investigate their cytotoxic potential. Methylation of 1 using methyl iodide in the presence of sodium carbonate produced the dimethylated product 1d (94%), whereas methylation of 2 yielded both a dimethylated product 2d (45%) and a monomethylated product 2e (14%). The ^1^H NMR data of 1d showed two methoxy signals at *δ*_H_ 4.00 and 3.99, whereas 2d displayed two methoxy signals at *δ*_H_ 4.00 and 3.92. The ^1^H NMR data of 2e exhibited a singlet signal of a methoxy group at *δ*_H_ 4.01, while the HMBC spectrum revealed a correlation between these methoxy protons to C-5, confirming the substitution at the C-5 position. Ethylation of 1 using ethyl iodide in the presence of sodium carbonate provided diethylated product 1e (86%), while 2 produced diethylated 2f (70%) and monoethylated 2g (27%) derivatives. In the alkylation reactions, 2 reacted with alkyl halide to yield both mono- and dialkylated products, which might due to the steric hindrance of the isoprenyl group at the C-4 position. Lastly, brominated xanthones was reported to demonstrate notable antibacterial activities against *S. aureus* and MRSA.^28^ Consequently, the bromination reaction was applied to the parent xanthones 1 and 2 to analyze their antibacterial efficacy. Bromination of 1 using *N*-bromosuccinimide (NBS) in aqueous acetonitrile at room temperature afforded 4-bromoxanthone 1f (36%). The ^1^H NMR data of 1f showed the absence of the aromatic singlet signal around *δ*_H_ 6.4 corresponding to H-4, while the ^13^C NMR data of 1f showed a characteristic signal for the C-4 brominated carbon at *δ*_C_ 81.9, supporting the bromination position. In contrast, the reaction of 2 under the same bromination conditions did not yield any brominated product, due to the less nucleophilicity of the aromatic ring B.

### 2.2 Biological activity

Thirteen xanthone derivatives 1a–f and 2a–g were evaluated for their cytotoxicity against human cervical cancer cells (HelaS3), human lung cancer cells (A549), human liver cancer cells (HepG2), and African green monkey kidney cells (Vero), and the result is summarized in Table 1. The acetyl derivative 1a displayed cytotoxicity against HelaS3, A549, and HepG2 cell lines, with IC_50_ values of 22.61 ± 2.02, 12.20 ± 1.08, and 21.10 ± 1.18 μM, respectively. Notably, 1a exhibited twice cytotoxic potency against A549 cells compared to its parent xanthone 1. Moreover, 1a showed no cytotoxicity against normal cells (IC_50_ > 100 μM). Compound 1b displayed strong toward A549 with IC_50_ values of 5.77 ± 2.14 μM, approximately fourfold higher than its parent 1. However, 1b also exhibits strong cytotoxicity to normal cells. In addition, 1b demonstrated cytotoxicity against HepG2 with IC_50_ values of 17.15 ± 4.50 μM comparable to 1. The acetyl derivative 2a exhibited moderate cytotoxicity against three cancer cell lines with IC_50_ values in the range of 15.66 ± 1.17 to 25.55 ± 4.77 μM. The pentanoyl derivative 2b showed weak cytotoxicity against HelaS3 (IC_50_ = 55.31 ± 2.18 μM) and A549 (IC_50_ = 41.84 ± 1.91 μM) cancer cells. This result indicated that the acyl derivatives of 2 decrease the cytotoxicity against the cancer cell lines tested. The mesylated derivatives, 1c and 2c, displayed weak cytotoxicity against HelaS3 cell line, with IC_50_ values of 65.33 ± 3.92 and 84.26 ± 7.86 μM, respectively. In contrast, the dimethylated products 1d and 2d, as well as the ethylated derivatives 2f and 2g, showed no cytotoxicity for all cancer cells tested. This suggested that the alkylation derivatives at the 5-OH and 6-OH positions of 1 and 2 reduce their cytotoxic potency. The bromoxanthone derivative 1f demonstrated moderate cytotoxicity to HelaS3 and HepG2 cell lines, with IC_50_ values of 17.43 ± 1.75 and 19.42 ± 1.52 μM, respectively. In addition, 1f exhibited strong cytotoxicity against A549 (IC_50_ = 7.52 ± 0.36 μM), which is threefold higher than that of its parent 1. However, 1f showed cytotoxic toward normal cells. It was found that others derivative 2 in this study eliminated the cytotoxicity of all cancer cell lines tested. In conclusion, acylation and bromination of 1 enhance the cytotoxicity toward A549 cell line, but only acetyl 1a has the potential to be developed as an anticancer drug targeting A549 cells, as it has anticancer activity without causing toxicity to normal cells (IC_50_ > 100 μM).

**Table 1:** *In vitro* cytotoxicity of toxyloxanthone C and macluraxanthone derivatives^a^

Compound	Cytotoxicity (IC_50_, μM)			
	HelaS3	A549	HepG2	Vero
1a	22.61 ± 2.02	12.20 ± 1.08	21.10 ± 1.18	>100
1b	20.46 ± 4.92	5.77 ± 2.14	17.15 ± 4.50	5.59 ± 0.36
1c	65.33 ± 3.92	>100	>100	>100
1d	>100	>100	>100	>100
1e	50.39 ± 8.45	>100	>100	>100
1f	17.43 ± 1.75	7.52 ± 0.36	19.42 ± 1.52	9.52 ± 1.02
2a	25.55 ± 4.77	15.66 ± 1.17	24.54 ± 2.98	10.10 ± 1.67
2b	55.31 ± 2.18	41.84 ± 1.91	>100	35.95 ± 2.32
2c	84.26 ± 7.86	48.83 ± 4.73	>100	>100
2d	>100	>100	>100	>100
2e	>100	90.46 ± 6.23	>100	>100
2f	>100	>100	>100	>100
2g	>100	>100	>100	>100
1	13.55 ± 1.11	21.78 ± 4.89	20.81 ± 2.57	8.52 ± 0.64
2	1.59 ± 0.12	6.46 ± 0.98	5.26 ± 0.41	4.29 ± 0.60
Doxorubicin	0.29 ± 0.02	0.48 ± 0.036	0.37 ± 0.02	1.95 ± 0.13

a HelaS3 = human cervical carcinoma, A549 = human lung carcinoma, HepG2 = human hepatocellular carcinoma, Vero = African green monkey kidney.

All xanthone derivatives 1a–f and 2a–g were further evaluated for antibacterial activity against four Gram-positive bacteria, including MRSA, *S. aureus*, *B. subtilis*, and *B. cereus,* using the microdilution method (Table 2). Compounds 1a and 1f exhibited significant antibacterial efficacy against all bacterial strains with the same MIC value at 4 μg mL^−1^, representing 32-fold improvement in potency compared to their parent 1. Similarly, 1b also showed notable activity with MIC values ranging from 8 to 16 μg mL^−1^, indicated their increasing potency activity compared to its parent 1. This suggested that acylation and bromination of 1 enhanced antibacterial activity. Compounds 1c, 1d, and 1e showed no antibacterial activity against all bacterial strains. It should be noted that sulfonylation and alkylation at the 5-OH and 6-OH positions decreased the antibacterial activity. In case of derivatives of 2, only the acetyl derivative 2a exhibited antibacterial activity against all tested bacteria with MIC values ranging from 8 to 32 μg mL^−1^, which showed more effective than its parent xanthone. While the other derivatives 2b–g exhibited no antibacterial activity against all tested bacteria. This revealed that the sulfonylation and alkylation of 2 reduced their antibacterial activity.

**Table 2:** Antibacterial activity of toxyloxanthone C and macluraxanthone derivatives

Compound	MIC (μg mL^−1^)			
	MRSA^a^	*S. aureus* b	*B. subtilis* c	*B. cereus* d
1a	4	4	4	4
1b	8	8	8	16
1c	>128	>128	>128	>128
1d	>128	>128	>128	>128
1e	>128	>128	>128	>128
1f	4	4	4	4
2a	8	16	32	16
2b	>128	>128	>128	>128
2c	>128	>128	>128	>128
2d	>128	>128	>128	>128
2e	>128	>128	>128	>128
2f	>128	>128	>128	>128
2g	>128	>128	>128	>128
1	128	128	128	128
2	16	>128	64	64
Vancomycin	2	2	2	1

a Methicillin resistant *Staphylococcus aureus*

b *S. aureus* ATCC 25923.

c Bacillus subtilis ATCC 6633.

d Bacillus cereus ATCC 11778.

### 2.3 Molecular docking simulation

**Fig. 1:**
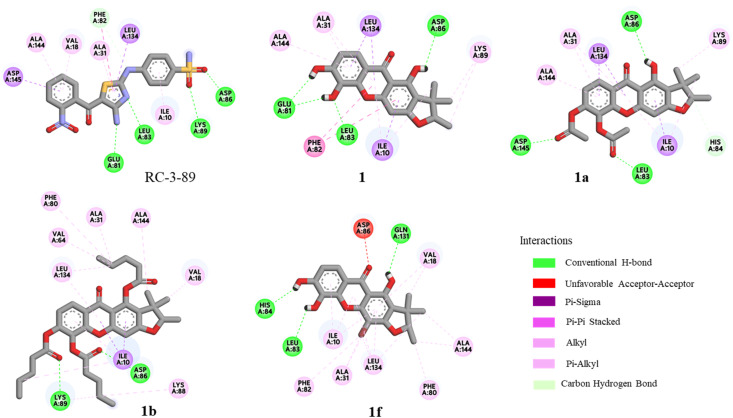
The binding interactions of RC-3-89, 1, 1a, 1b, and 1f with CDK2.

**Fig. 2:**
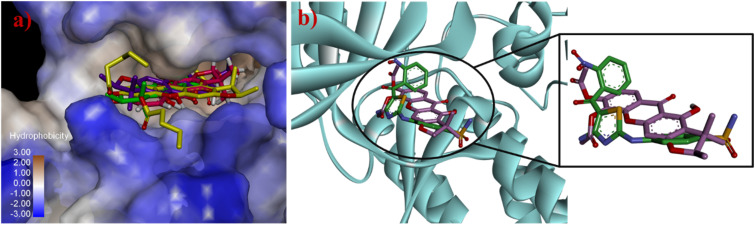
(a) Docking poses of 1 (green),1a (purple), 1b (yellow), and 1f (pink) onto the active site of CDK2 and (b) superimposed conformation of co-crystallized ligand (green) and 1a (purple) onto the active site of CDK2.

Molecular docking simulations were performed using the AutoDock program to elucidate the binding interactions of the active xanthones (1, 1a, 1b, and 1f) with cyclin-dependent kinase 2 (CDK2). As a key regulator of the cell cycle, CDK2 is frequently overexpressed in various cancers, resulting in dysregulated proliferation and tumor progression.^29^ Therefore, the inhibition of CDK2 could be a pharmacological strategy for cancer treatment.^29–32^ The crystal structure of CDK2 in complex with the inhibitor RC-3-89 was validated by a redocking experiment, and the results are summarized in Fig. 1 and Table 3. The ligand RC-3-89 occupied the ATP binding site of CDK2, interacting with key amino acid residues in the hinge region (Glu81, Phe82, and Leu83), the front specificity pocket (Asp86 and Lys89), the DFG motif (Asp145), and the hydrophobic pocket (Ile10, Val18, Ala31, and Leu134).^29^ Consistently, compounds 1, 1a, 1b, and 1f were also accommodated within the ATP binding site of CDK2 in the hinge region, suggesting a competitive inhibition mechanism (Fig. 2a).^32,33^ The xanthone core structure of all docking compounds formed hydrophobic interactions in the hydrophobic pocket of CDK2, as shown in Fig. 1. The docking analysis of 1 revealed the formation of three hydrogen bonds in the hinge region, including the interactions of 5-OH with Leu83 and Glu81, as well as 6-OH with Glu81. Compound 1 formed hydrogen bond with Asp86 and pi-alkyl with Lys89 in the front specificity pocket. Compound 1a interacted with CDK2 through hydrogen bonding with Leu83, Asp86, and Asp145, consistent with the binding interaction observed for the reference ligand RC-3-89, as shown in Fig. 1 and 2b. Compound 1b demonstrated the lowest binding energy among the xanthone derivatives, with a binding energy of −9.78 kcal mol^−1^. The two carbonyl moieties of pentanoyl groups formed hydrogen bonds with Asp86 and Lys89 amino acids. Additionally, the side chains of pentanoyl moieties created hydrophobic interactions in the hydrophobic pocket with Ile10, Ala31, and Leu134 as well as with the gatekeeper residue Phe80. The results suggested that the pentanoyl substitutions may enhance the stability of the compound within the hydrophobic pocket of the ATP binding site. Docking analysis of 1f demonstrated hydrogen bonding interactions of 1-OH, 5-OH, and 6-OH with Gln131, Leu83, and His84, respectively. Additionally, the bromine atom formed hydrophobic interactions with Ala31, Phe82, and Leu134 residues, which may play a crucial role in stabilizing the protein-ligand interaction.

**Table 3:** Summary of the binding interactions and energies of active xanthone with CDK2, PBP2a of MRSA, and SCWP *O*-acetyltransferase of *B. cereus*

Compound	Binding energy (kcal mol^−1^)	Protein-ligand interactions		
		Hydrogen bond	Hydrophobic	Electrostatic
**Interactions of xanthone with CDK2**				
1	−8.62	Glu81, Leu83, Asp86	Ile10, Ala31, Phe82, Lys89, Leu134, Ala144	—
1a	−9.43	Lue83, Asp86, Asp145	Ile10, Ala31, His84, Lys89, Leu134, Ala144	—
1b	−9.78	Asp86, Lys89	Ile10, Val18, Ala31, Phe80, Phe82, Leu134, Ala144	—
1f	−8.52	Leu83, His84, Gln131	Ile10, Val18, Ala31, Phe80, Phe82, Ala144, Lue134	—
RC-3-89	−10.51	Glu81, Leu83, Asp86, Lys89	Ile10, Val18, Ala31, Phe82, Leu134, Ala144, Asp145	—

**Interactions of xanthone with PBP2a of MRSA**				
1a	−8.02	Ser403, Glu602, Asn464, Met641	Tyr446, His583, Ala642	—
1f	−7.29	Ser403, Ser462, Ser598	Asn464, Tyr519, Met641	—
Tatracycline	−8.35	Ser403, Tyr444, Try446, Ser462, Asn464, Ser598	Gln521, Ala646, Met641	—

**Interactions of xanthone with *B. subtilis* LCP enzyme**				
1a	−7.55	Thr197, Arg210, Gln214	Asp75, Asp85, Arg106, Arg210	Asp75, Asp85
1f	−7.32	Gln211	Pro105, Arg106, Asp207, Phe208, Leu273, Tyr286	Asp107, Asp207
Tatracycline	−5.86	Asp75, Arg210, Gln214	Asp75, Asp85, Arg198, Arg210	Arg198

**Interactions of xanthone with SCWP *O*-acetyltransferase of *B. cereus***				
1a	−7.18	Lys103, Asn104, Asn251, Arg359	His201, His202, Ser249, Phe250	His201
1f	−6.97	Lys103, Asn251, Ser385	His201	Asp92, His201, Arg359
Tetracycline	−5.75	Lys103, Asn104, Asn251, Ser385	His201, His202, Asn386	Arg359

**Fig. 3:**
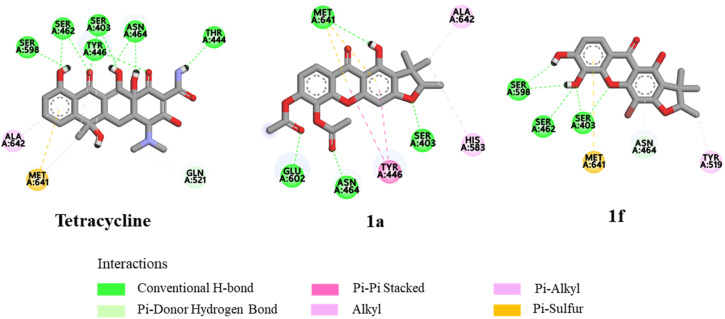
The binding interactions of tetracycline, 1a, and 1f with the PBP2a of MRSA.

**Fig. 4:**
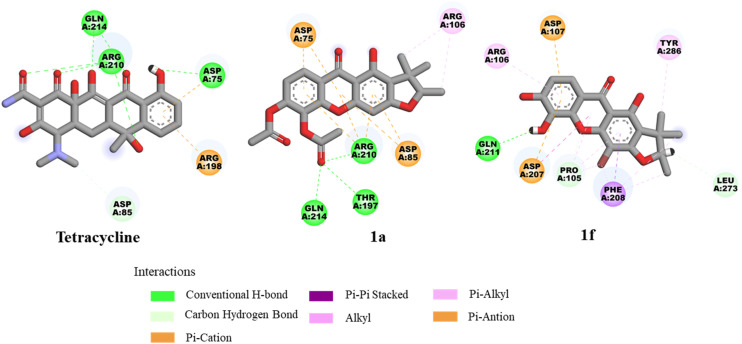
The binding interactions of tetracycline, 1a, and 1f with *B. subtilis* LCP enzyme.

**Fig. 5:**
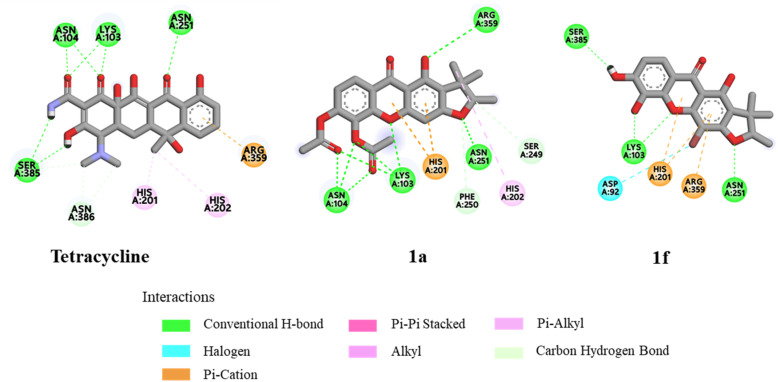
The binding interactions of tetracycline, 1a, and 1f with SCWP *O*-acetyltransferase of *B. cereus*.

**Fig. 6:**
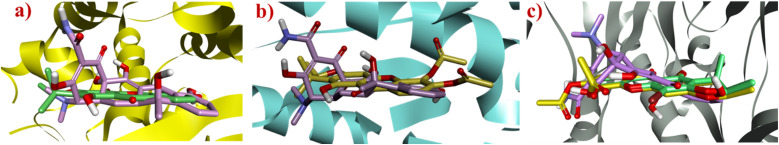
The superimposed conformations of (a) tetracycline (purple) and 1f (green) with the PBP2a of MRSA, (b) tetracycline (purple) and 1a (yellow) with *B. subtilis* LCP enzyme, and (c) tetracycline (purple), 1a (yellow), and 1f (green) with SCWP *O*-acetyltransferase of *B. cereus*.

Molecular docking studies were investigated to support the *in vitro* antibacterial activity toward MRSA, *B. subtilis*, and *B. cereus*, using the penicillin-binding protein 2a (PBP2a) from MRSA,^34^ the *B. subtilis* TagU,^35,36^ and the secondary cell wall polysaccharides (SCWP) *O*-acetyltransferase of *B. cereus*^37^ as the protein templates. The PBP2a is crucial transpeptidase enzyme for the cell wall synthesis and β-lactam antibiotic resistance in MRSA.^34,38^ In contrast, the *B. subtilis* LytR–CpsA–Psr (LCP) enzyme play a crucial role in cell wall assembly and maintenance. This enzyme is responsible for transferring wall teichoic acids and capsular polysaccharides onto the peptidoglycan of Gram-positive bacteria.^35^ SCWP *O*-acetyltransferase is an essential enzyme for the stable binding of S-layer proteins to the bacterial cell wall, resulting in the enhanced structural integrity of cell wall.^37^ Therefore, the molecular docking of these key enzymes involved in cell wall synthesis of bacterial pathogens was performed to elucidate their potential for treating bacterial infections. Tetracycline was investigated as a control for all proteins and the results are summarized in Table 3. The active site of the PBP2a of MRSA consists of the amino acid residues Ser403, Lys406, Tyr446, Ser462, Asn464, Ser598, Gly599, Thr600, and Met641.^34^ Tetracycline exhibited the lowest binding energy of −8.35 kcal mol^−1^ and formed hydrogen bonds with key amino acid residues Ser403, Tyr446, Ser462, Asn464, and Ser598 within the PBP2a active site of the transpeptidase domain (Fig. 3). Compounds 1a and 1f demonstrated the binding energy of −8.02 and −7.29 kcal mol^−1^, respectively, within the PBP2a active site. Compound 1a established hydrogen bonds with Ser403, Asn464, and Met641 and formed pi–pi stacking with Tyr446. Significantly, the carbonyl of acetyl groups formed hydrogen bonds with Glu602 and Asn464, indicating that the acetyl groups may play an important role in antibacterial against MRSA. Compound 1f established hydrogen bonds with Ser403, Ser462, and Ser598. Moreover, the superimposed conformation of 1f in the PBP2a active site was similar to those of tetracycline, as shown in Fig. 6a. According to the docking results of *B. subtilis*, the amino acids Asp75, Arg83, Asp85, Arg106, Asp107, Lys120, Phe156, Arg198, Arg200, Asp207, and Arg210 are considered as the active site of *B. subtilis* LCP enzyme.^35^ Tetracycline showed a binding energy of −5.86 kcal mol^−1^ and formed hydrogen bonds with Asp75, Asp207, Arg210, and Gln214 (Fig. 4). Compound 1a demonstrated the lowest binding energy of −7.55 kcal mol^−1^. Its xanthone core structure formed electrostatic interactions with key amino residues Asp75, Asp85, and Arg210. The carbonyl group at C-5 formed three hydrogen bonds with Thr197, Arg210, and Gln214 amino acids. The docking poses of 1a exhibited similar orientation to that of tetracycline in the active site (Fig. 6b). Compound 1f established a hydrogen bond with Gln211, while its bromine atom interacted with Pro105 and Phe208 through hydrophobic interactions. For the SCWP *O*-acetyltransferase molecular docking result of *B. cereus*, the amino acid residues Lys186, His201, His202, Ser337, Ser354, Arg359, and Ser364 are considered as the active site of SCWP *O*-acetyltransferase of *B. cereus*.^37^ The binding energy of tetracycline was calculated to be −5.75 kcal mol^−1^ (Table 3). Docking analysis revealed that tetracycline formed hydrogen bonds with Lys103, Asn104, Asn251, and Ser385. Additionally, it exhibited a pi–cation interaction with Arg359 and pi-alkyl interactions with His201 and His202 (Fig. 5). Compound 1a displayed a hydrogen bond and a pi cation interaction with the key amino acids Arg359 and His201, respectively. Furthermore, the acetyl groups of 1a established hydrogen bonds with the Lys103 and Asn104 residues, indicating that the acetyl groups could improve antibacterial toward *B. cereus*. Compound 1f formed three hydrogen bonds with Lys103, Asn251, and Ser385 and pi–cation interactions with the key amino acids His201 and Arg359. The bromine atom established a halogen bond with Asp92, suggesting its role in stabilizing the protein-ligand interaction. Moreover, the binding energy of 1a and 1f was lower than that of tetracycline. Structural superimpositions of 1a, 1f, and tetracycline further confirmed their similar binding interaction within the active site (Fig. 6c).

### 2.4 Physicochemical properties

Lipinski's and Veber's rules were used to evaluate the drug-likeness of all compounds, and the results are summarized in Table 4. Lipinski's rule considers many criteria, including molecular weight (MW ≤ 500), hydrogen bond acceptors (HBA ≤ 10), hydrogen bond donors (HBD ≤ 5), and log *P* (lipophilicity index <5).^39^ A compound that violates more than one of these criteria is less likely to be an orally active drug. All compounds, except for 1b and 2b, meet these criteria, suggesting their potential for good oral bioavailability. These indicated that the structural modification of both parent compounds with pentanoyl chloride tends to compromise their drug-like properties. While Veber's rule considers the number of rotatable bonds (NORTB ≤ 10) and topological polar surface area (TPSA ≤ 140).^40^ Most compounds, with the exception of 1b, 2b, 1c, and 2c, comply with both criteria, supporting the favorable oral absorption. The results of Lipinski's and Veber's rules indicated that compounds 1a and 1f could be developed to be the orally active drug.

**Table 4:** Physicochemical properties of modified and parent compounds by *in silico* analysis

Compound	MW^a^ (g mol^−1^)	LogP^b^	HBA^c^	HBD^d^	NORTB^e^	TPSA^f^ (Å^2^)
1a	412.39	3.35	8	1	4	112.27
1b	580.67	6.64	9	0	15	118.34
1c	484.50	2.93	10	1	4	163.17
1d	356.37	3.49	6	1	2	78.13
1e	384.42	4.14	6	1	4	78.13
1f	407.21	3.32	6	3	0	100.13
2a	478.49	4.59	8	1	6	112.27
2b	646.77	7.69	9	0	17	118.34
2c	550.60	4.09	10	1	6	163.17
2d	422.47	4.68	6	1	4	78.13
2e	408.44	4.37	6	2	3	89.13
2f	450.52	5.33	6	1	6	78.13
2g	422.47	4.64	6	2	4	89.13
1	328.32	2.74	6	3	0	100.13
2	394.42	3.95	6	3	2	100.13

a Molecular weight.

b Octanal/water partition coefficient.

c Number of hydrogen bond acceptors.

d Number of hydrogen bond donors.

e Number of rotatable bonds.

f Topological polar surface area.

## 3 Experimental

### 3.1 General experimental procedures

Melting points were determined on a SANYO Gallenkamp (Leicester, UK) melting point apparatus and are uncorrected. IR spectra were taken on a PerkinElmer Spectrum One spectrophotometer (Agilent Technologies, U.S.A.). NMR spectra were recorded in CDCl_3_ and CD_3_OD on a Bucker Ascend 400 MHz (Bruker, Germany). Mass spectra were performed on an Agilent 1260 Infinity series high-performance liquid chromatography system (Agilent, Waldbronn, Germany), coupled with a 6540 ultra-high-definition accurate mass spectrometer (Agilent Technologies, Singapore). Column chromatography was carried out on SiliCycle silica gel (40–63 μm, SiliCycle, Inc., Canada), TLC was performed with precoated Merck silica gel 60 PF254 on an aluminum (Merck, Darmstadt, Germany).

### 3.2 Plant material

Roots of *Maclura cochinchinensis* were collected from Pattani province, Thailand.^4^ The specimens were identified by Prof. Pranom Chantaranothai, Department of Biology, Khon Kaen University, Thailand, where a voucher specimen (SK17) was deposited.

### 3.3 Preparation of xanthone derivatives

#### 3.3.1 Isolation of toxyloxanthone C (1) and macluraxanthone (2)

The starting xanthone, macluraxanthone (2), was obtained from the crude hexane and EtOAc extracts of the root of *M. cochinchinensis*. Meanwhile, toxyloxanthone C (1) was recrystallized from the solid of subfraction E7.1 using MeOH as a solvent, following the procedure described in a previous publication.^4^

Toxyloxanthone C (1); yellow solid; [*α*]_D_^24^ +17.7 (*c* 0.36, acetone);^41^^1^H NMR (400 MHz, (CH_3_)_2_CO) *δ* 13.45 (1H s, 1-OH), 7.60 (1H, d, *J* = 8.4 Hz, H-8), 6.96 (1H, d, *J* = 8.4 Hz, H-7), 6.30 (1H, s, H-4), 4.50 (1H, q, *J* = 6.8 Hz, H-12), 1.46 (s, 3H, H-14), 1.36 (3H, d, *J* = 6.4 Hz, H-13), 1.22 (3H, s, H-15); ^13^C NMR (100 MHz, (CH_3_)_2_CO) *δ* 181.2 (C-9), 166.2 (C-1), 159.1 (C-3), 158.2 (C-4a), 151.6 (C-6), 146.5 (C-10a), 132.7 (C-5), 117.0 (C-8), 116.9 (C-8a), 114.2 (C-2), 113.3 (C-7), 103.6 (C-9a), 91.3 (C-12), 89.6 (C-4), 43.6 (C-11), 25.0 (C-14), 20.5 (C-15), 14.2 (C-13); HRESI-MS *m*/*z* 329.1025 [M + H]^+^ (Calc. for 329.1025, C_18_H_17_O_6_).

Macluraxanthone (2); yellow crystals; ^1^H NMR (400 MHz, CD_3_OD) *δ* 7.56 (1H, d, *J* = 8.8 Hz, H-8), 6.88 (1H, d, *J* = 8.4 Hz, H-7), 6.69 (1H, d, *J* = 10.0 Hz, H-11), 6.44 (1H, dd, *J* = 17.6, 10.4 Hz, H-19), 5.64 (1H, d, *J* = 10.0 Hz, H-12), 4.99 (1H, dd, *J* = 17.6, 1.2 Hz, H-20a), 4.84 (1H, dd, *J* = 10.4, 1.2 Hz, H-20b), 1.73 (6H, s, H-17,H-18), 1.47 (6H, s, H-14,H-15); ^13^C NMR (100 MHz, CD_3_OD) *δ* 182.5 (C-9), 160.1 (C-1), 157.3 (C-3), 156.6 (C-4a), 152.8 (C-19), 152.7 (C-6), 147.5 (C-10a), 134.2 (C-5), 128.4 (C-12), 117.1 (C-11), 116.8 (C-8), 114.9 (C-8a), 114.6 (C-7), 113.7 (C-4), 107.7 (C-20), 106.1 (C-2), 103.9 (C-9a), 79.3 (C-13), 42.2 (C-16), 30.2 (C-17,C-18), 28.1 (C-14,C-15); HRESI-MS: *m*/*z* 395.1496 [M + H]^+^ (Calc. for C_23_H_23_O_6_ 395.4195).

#### 3.3.2 General procedure to prepare 1a–c and 2a–c^42,43^

Toxyloxanthone C (1) (24.3 mg, 0.074 mmol) or macluraxanthone (2) (27.9 mg, 0.071 mmol) was dissolved in pyridine (2 mL), followed by the dropwise addition of an excess of acetic anhydride (Ac_2_O) at 0 °C. The reaction mixture was stirred at room temperature for 2 hours. The reaction mixture was poured into cold water (20 mL) and extracted with EtOAc (2 × 20 mL). The organic layer was combined, washed with water and brine, dried over anhydrous Na_2_SO_4_, filtered, and evaporated under reduced pressure to obtain a crude solid. Recrystallization of a crude solid from hexane yielded 1a (12.8 mg, 41%) or 2a (22 mg, 65%).

The reactions of 1 or 2 with pentanoyl chloride or methanesulfonyl chloride were performed following the same procedure described above, affording 1b (10.2 mg, 11%), 1c (27.3 mg, 77%), 2b (19.9 mg, 19%), and 2c (34.3 mg, 88%), respectively.

##### 3.3.2.1 General procedure to prepare 1d–e and 2d–g^42^

To a solution of toxyloxanthone C (1) (30.0 mg, 0.089 mmol) or macluraxanthone (2) (59.2 mg, 0.15 mmol) in dry acetone (2 mL), anhydrous sodium carbonate (160 mg), and methyl iodide (0.12 mL) were added. The reaction mixture was refluxed overnight. The reaction was quenched by the addition of cold water and extracted with EtOAc (2 × 20 mL). The organic combined layer was washed with water, brine, and dried over anhydrous Na_2_SO_4_. Evaporation of the solvent gave a crude oil. The crude product was purified by silica gel column chromatography using 20% EtOAc/hexane as an eluent, yielding 1d (30.2 mg, 94%) or 2d (28.6 mg, 45%) and 2e (8.6 mg, 14%).

The reaction of 1 (49.0 mg, 0.149 mmol) or 2 with ethyl iodide (EtI) (59.1 mg, 0.149 mmol) was performed in the same procedure as described above, yielding 1e (42.3 mg, 86%), or 2f (41.7 mg, 70%) and 2g (13.4 mg, 27%).

#### 3.3.3 Bromination using *N*-bromosuccinimide (NBS)^43^

Toxyloxanthone C (1) (30.8 mg, 0.094 mmol) was dissolved in EtOAc (2 mL), and a solution of NBS (0.155 mmol) in MeCN : H_2_O (1 : 1, 2 mL) was added dropwise at 0 °C. The reaction mixture was stirred at 0 °C for 1 hour, followed by stirring at room temperature for 1 hour. The reaction mixture was poured into cold water (20 mL) and extracted with EtOAc (2 × 20 mL). The organic layer was combined, washed with water and brine, dried over anhydrous Na_2_SO_4_, and concentrated under reduced pressure to afford a crude solid. Recrystallization from hexane yielded 1f (24.2 mg, 36%). Bromination of macluraxanthone (2) (37.3 mg, 0.094 mmol) using the same procedure resulted in no product formation.

### 3.4 Spectroscopic data

5,6-Diacetoxytoxyloxanthone C (1a); yellow solid; mp 164.1–165.3 °C; IR *ν*_max_ (cm^−1^); 2965, 2915, 1775, 1648, 1579, 1428, 1370, 1260; ^1^H NMR (400 MHz, CDCl_3_) *δ* 12.84 (1H, s, 1-OH), 8.14 (1H, d, *J* = 8.8, 0.8 Hz, H-8), 7.20 (1H, d, *J* = 8.8, 0.8 Hz, H-7), 6.29 (1H, s, *J* = 0.8 Hz, H-4), 4.52 (1H, q, *J* = 6.8 Hz, H-12), 2.43 (3H, s, H-2′′), 2.35 (3H, s, H-2′), 1.50 (3H, s, H-14), 1.39 (3H, d, *J* = 6.8 Hz, H-13), 1.26 (3H, s, H-15); ^13^C NMR (100 MHz, CDCl_3_) *δ* 179.9 (C-9), 167.7 (C-1′′), 167.4 (C-1′), 166.5 (C-1), 158.8 (C-3), 157.6 (C-4a), 149.2 (C-6), 147.4 (C-10a), 130.1 (C-5), 123.5 (C-8), 119.6 (C-8a), 118.6 (C-2), 117.6 (C-7), 104.2 (C-9a), 91.4 (C-12), 90.1 (C-4), 43.4 (C-11), 25.3 (C-2′′), 20.8 (C-2′), 20.7 (C-14), 20.4 (C-15), 14.5 (C-13); HRESI-MS *m*/*z* 413.1245 [M + H]^+^ (Calc. for 413.1236, C_22_H_21_O_8_).

1,5,6-Tripentanoyloxytoxyloxanthone C (1b); white solid; mp 85.2–87.9 °C; IR *ν*_max_ (cm^−1^); 2958, 2935, 2873, 1765, 1658, 1456, 1418, 1257; ^1^H NMR (400 MHz, CDCl_3_) *δ* 8.13 (1H,d, *J* = 8.8 Hz, H-8), 7.15 (1H, d, *J* = 8.8 Hz, H-7), 6.47 (1H, s, H-4), 4.59 (1H, q, *J* = 6.4 Hz, H-12), 2.77 (2H, t, *J* = 7.6 Hz, H-2′′′), 2.67 (2H, t, *J* = 7.6 Hz, H-2′′), 2.59 (2H, t, *J* = 7.6 Hz, H-2′), 1.97–1.67 (6H, m, H-3′′′, H-3′′ and H-3′), 1.54 (3H, s, H-14), 1.48 (6H, m, H-4′′′, H-4′′ and H-4′), 1.43 (3H, d, *J* = 6.4 Hz, H-13), 1.43 (3H, s, H-15), 1.10–0.92 (9H, m, H-5′′′, H-5′′, H-5′); ^13^C NMR (100 MHz, CDCl_3_) *δ* 173.8 (C-9), 172.6 (C-1?), 170.6 (C-1′′), 170.1 (C-1′), 164.1 (C-1), 154.0 (C-3), 152.2 (C-4a), 148.5 (C-6), 147.3 (C-10a), 130.8 (C-5), 124.4 (C-8), 121.2 (C-8a), 120.1 (C-2), 118.7 (C-7), 109.5 (C-9a), 103.6 (C-12), 91.4 (C-4), 44.5 (C-11), 34.2 (C-2′′′), 33.9 (C-2′′), 33.6 (C-2′), 27.0 (C-3′′′), 26.9 (C-3′′), 26.7 (C-3′), 25.8 (C-14), 22.42 (C-4′′′), 22.38 (C-4′′), 22.38 (C-4′), 21.3 (C-15), 14.4 (C-13), 14.0 (C-5′′′), 13.9 (C-5′′), 13.8 (C-5′); HRESI-MS *m/z* 581.2718 [M + H]^+^ (Calc. for 581.2751, C_33_H_41_O_9_).

5,6-Dimesyloxytoxyloxanthone C (1c); yellow solid; mp 191.1–192.3 °C; IR *ν*_max_ (cm^−1^); 3020, 2940, 1648, 1597, 1448, 1355, 1274; ^1^H NMR (400 MHz, CDCl_3_) *δ* 12.65 (1H, s, 1-OH), 8.20 (1H, d, *J* = 8.8 Hz, 1H, H-8), 7.48 (1H, d, *J* = 8.8 Hz, H-7), 6.30 (1H, s, H-4), 4.55 (1H, q, *J* = 6.8 Hz, H-12), 3.49 (3H, s, 6-OSO_2_CH_3_), 3.38 (3H, s, 5-OSO_2_CH_3_), 1.50 (3H, s, H-14), 1.40 (d, *J* = 6.8 Hz, 3H, H-13), 1.26 (s, 3H, H-15); ^13^C NMR (400 MHz, CDCl_3_) *δ* 180.0 (C-9), 166.9 (C-1), 158.9 (C-3), 157.3 (C-4a), 149.5 (C-6), 146.6 (C-10a), 130.1 (C-5), 125.1 (C-8), 120.6 (C-8a), 118.6 (C-2), 118.3 (C-7), 104.0 (C-9a), 91.7 (C-12), 89.9 (C-4), 43.5 (C-11), 40.3 (6-OSO_2_CH_3_), 39.5 (5-OSO_2_CH_3_), 25.3 (C-14), 20.7 (C-15), 14.5 (C-13); HRESI-MS *m/z* 485.0579 [M + H]^+^ (Calc. for 485.0576, C_20_H_21_O_10_S_2_).

5,6-Dimethoxytoxyloxanthone C (1d); yellow solid; mp 142.7–145.9 °C; IR *ν*_max_ (cm^−1^); 3069, 2958, 2938, 2880, 2844, 1648, 1590, 1428, 1282, 1212; ^1^H NMR (400 MHz, CDCl_3_) *δ* 13.10 (1H s, 1-OH), 7.97 (1H, d, *J* = 8.8 Hz, H-8), 7.95 (1H, d, *J* = 8.8 Hz, H-7), 6.40 (1H, s, H-4), 4.50 (1H, q, *J* = 6.8 Hz, H-12), 4.00 (3H, d, 6-OCH_3_), 3.99 (3H, d, 5-OCH_3_), 1.50 (s, 3H, H-14), 1.40 (3H, d, *J* = 6.4 Hz, H-13), 1.25 (3H, s, H-15); ^13^C NMR (100 MHz, CDCl_3_) *δ* 180.7 (C-9), 166.1 (C-1), 158.8 (C-3), 158.0 (C-4a), 157.6 (C-6), 150.3 (C-10a), 136.2 (C-5), 121.5 (C-8), 117.0 (C-8a), 115.5 (C-2), 108.6 (C-7), 103.8 (C-9a), 91.2 (C-12), 89.9 (C-4), 61.7 (6-OCH_3_), 56.6 (5-OCH_3_), 43.4 (C-11), 25.3 (C-14), 20.7 (C-15), 14.4 (C-13); HRESI-MS *m*/*z* 357.1346 [M + H]^+^ (Calc. for 357.1338, C_20_H_21_O_6_).

5,6-Diethoxytoxyloxanthone C (1e); yellow solid; mp 132.3–132.5 °C; IR *ν*_max_ (cm^−1^); 2977, 1656, 1604 1569, 1440, 1284, 1146; ^1^H NMR (400 MHz, CDCl_3_) *δ* 13.13 (1H s, 1-OH), 7.92 (1H, d, *J* = 8.8 Hz, H-8), 6.94 (1H, d, *J* = 8.8 Hz, H-7), 6.38 (1H, s, H-4), 4.50 (1H, q, *J* = 6.4 Hz, H-12), 4.19 (4H, q, *J* = 6.8 Hz, H-1′′,1′), 1.50 (6H, m, H-14, H-2′′) 1.41 (6H, m, H-13, H-2′), 1.25 (s, 3H, H-15); ^13^C NMR (100 MHz, CDCl_3_) *δ* 180.7 (C-9), 165.9 (C-1), 158.8 (C-3), 158.1 (C-4a), 157.3 (C-6), 150.8 (C-10a), 135.3 (C-5), 121.2 (C-8), 116.9 (C-8a), 115.2 (C-2), 109.5 (C-7), 103.8 (C-9a), 91.1 (C-12), 89.9 (C-4), 69.8 (C-1′′), 64.9 (C-1′), 43.4 (C-11), 25.3 (C-15), 20.7 (C-14), 15.7 (C-2′′), 14.9 (C-2′) 14.4 (C-13); HRESI-MS *m*/z 385.1651 [M + H]^+^ (Calc. for C_22_H_25_O_6_, 385.1651).

4-Bromotoxyloxanthone C (1f); brown solid; mp 162.4–162.7 °C; IR *ν*_max_ (cm^−1^); 3595, 3165, 2959, 1646, 1607, 1579,1425, 1309 1247, 1203, 698; ^1^H NMR (400 MHz, CD_3_OD) *δ* 7.59 (1H, d, *J* = 8.4 Hz, H-8), 6.92 (1H, d, *J* = 8.4 Hz, H-7), 4.61 (1H, q, *J* = 6.4 Hz, H-12), 1.51 (3H, s, H-14), 1.44 (3H, d, *J* = 6.4 Hz, H-13), 1.25 (3H, s, H-15); ^13^C NMR (100 MHz, CD_3_OD) *δ* 182.1 (C-9), 164.2 (C-1), 158.7 (C-3), 155.1 (C-4a), 153.7 (C-6), 147.8 (C-10a), 133.9 (C-5), 118.3 (C-8), 117.7 (C-8a), 114.6 (C-2), 114.1 (C-7), 105.3 (C-9a), 92.8 (C-12), 81.9 (C-4), 45.8 (C-11), 25.6 (C-14), 20.9 (C-15), 14.5 (C-13); HRESI-MS *m*/z 407.1025 [M + H]^+^ (Calc. for C_18_H_16_BrO_6_, 407.1030), *m*/z 409.0111 [M+2 + H]^+^ (Calc. for C_18_H_16_BrO_6_ + 2, 409.0110).

5,6-Diacetoxymacluraxanthone (2a); yellow solid; mp 165.9–168.2 °C; IR *ν*_max_ (cm^−1^); 3728, 2927, 1625, 1453, 1374; ^1^H NMR (400 MHz, CDCl_3_) *δ* 13.4 (1H, s, 1-OH), 8.13 (1H, d, *J* = 8.8 Hz, H-8), 7.22 (1H, d, *J* = 8.8 Hz, H-7), 6.73 (1H, d, *J* = 10.0 Hz, H-11), 6.24 (1H, dd, *J* = 17.6, 10.4 Hz, H-19), 5.58 (1H, d, *J* = 10.0 Hz, H-12), 4.88 (1H, dd, *J* = 17.6, 1.2 Hz, H-20a) 4.84 (1H, dd, *J* = 10.4, 1.2 Hz, H-20b), 2.39 (3H, s, H-2′′) 2.34 (3H, s, H-2′) 1.65 (6H, s, H-17,H-18) 1.46 (6H, s, H-14,H-15); ^13^C NMR (100 MHz, CDCl_3_) *δ* 180.3 (C-9), 167.7 (C-1′), 167.6 (C-1′′), 160.1 (C-3), 156.7 (C-1), 154.9 (C-4a), 150.2 (C-19), 149.2 (C-6), 147.7 (C-10a), 130.8 (C-5), 127.7 (C-12), 123.5 (C-11), 119.1 (C-8), 118.8 (C-8a), 115.8 (C-7), 113.5 (C-4), 108.5 (C-20), 105.8 (C-2), 103.7 (C-9a), 78.7 (C-13), 41.2 (C-16), 30.1 (C-17,C-18), 28.1 (C-14,C-15), 20.8 (C-2′′), 20.6 (C-2′); HRESI-MS *m*/*z* 479.1706 [M + H]^+^ (Calc. for C_27_H_27_O_8_, 479.1706).

1,5,6-Tripentanoyloxymacluraxanthone (2b); yellow solid; mp 99.0–100.2 °C; IR *ν*_max_ (cm^−1^): 2958, 2933, 1766, 1652, 1595, 1460, 1130; ^1^H NMR (400 MHz, CDCl_3_) *δ* 8.09 (2H, d, *J* = 8.8 Hz, H-8), 7.15 (2H, d, *J* = 8.8 Hz, H-7), 6.44 (2H, d, *J* = 10.0 Hz, H-11), 6.28 (2H, dd, *J* = 17.6, 10.4 Hz, H-19), 5.72 (2H, d, *J* = 10.0 Hz, H-12), 4.86 (1H, dd, *J* = 17.6, 1.2 Hz, H-20a), 4.84 (1H, dd, *J* = 10.4, 1.2 Hz, H-20b), 2.82 (2H, t, *J* = 7.6 Hz, H-2′′′), 2.66 (2H, t, *J* = 7.6 Hz, H-2′′), 2.57 (2H, t, *J* = 7.6 Hz, H-2′), 1.78 (6H, m, H-3′, H-3′′, H-3′′′), 1.67 (6H, s, H-17, H-18), 1.48 (6H, m, H-4′, H-4′′, H-4′′′), 1.46 (6H, s, H-14,H-15), 1.00 (9H, m, H-5′, H-5′′, H-5′′′); ^13^C NMR (100 MHz, CDCl_3_) *δ* 174.5 (C-9), 172.1 (C-1′′′), 170.6 (C-1′′), 170.5 (C-1′), 157.6 (C-1), 156.4 (C-3), 150.0 (C-4a), 148.5 (C-19), 147.3 (C-6), 144.4 (C-10a), 131.5 (C-5), 130.7 (C-12), 123.9 (C-11), 120.6 (C-8), 120.5 (C-8a), 118.7 (C-7), 115.7 (C-4), 112.9 (C-20), 109.4 (C-2), 108.5 (C-9a), 78.4 (C-13), 41.7 (C-16), 34.2 (C-2′′′), 33.9 (C-2′′), 33.8 (C-2′), 30.0 (C-17, C-18), 28.0 (C-14, C-15), 26.9 (C-3′′′, C-3′′), 26.8 (C-3′), 22.6 (C-4′′′), 22.4 (C-4′′), 22.3 (C-4′), 14.0 (C-5′′′), 13.9 (C-5′′), 13.9 (C-5′); HRESI-MS: *m/z* 647.3219 [M + H]+ (Calc. for C_38_H_47_O_9_, 647.3220).

5,6-Dimesyloxymacluraxanthone (2c); yellow solid; mp 179.8–181.9 °C; IR *ν*_max_ (cm^−1^): 3011 2978 1652 1600 1576 1455 1408 1352 1291 1175; ^1^H NMR (400 MHz, CDCl_3_): *δ* 13.26 (1H, s, 1-OH), 8.24 (1H, d, *J* = 8.8 Hz, H-8), 7.48 (2H, d, *J* = 8.8 Hz, H-7), 6.73 (1H, d, *J* = 10.0 Hz, H-11), 6.34 (1H, dd, *J* = 17.6, 10.4 Hz, H-19), 5.61 (1H, d, *J* = 10.0 Hz H-12), 4.91 (1H, dd, *J* = 17.6, 1.2 Hz, H-20a), 4.84 (1H, dd, *J* = 10.4, 1.2 Hz, H-20b), 3.43 (3H, s, 6-OSO_2_CH_3_), 3.33 (3H, s, 5-OSO_2_CH_3_), 1.72 (6H, s, H-17,H-18), 1.47 (6H, s, H-14,H-15); ^13^C NMR (100 MHz, CDCl_3_) *δ* 179.5 (C-9), 160.7 (C-1), 156.6 (C-3), 154.9 (C-4a), 150.6 (C-19), 149.9 (C-6), 146.5 (C-10a), 129.7 (C-5), 128.0 (C-12), 125.3 (C-11), 120.4 (C-8), 117.6 (C-8a), 115.6 (C-7), 114.6 (C-4), 108.2 (C-20), 106.1 (C-2), 103.7 (C-9a), 79.0 (C-13), 41.2 (C-16), 40.3 (C-6-OSO_2_CH_3_), 39.0 (C-5-OSO_2_CH_3_), 30.0 (C-17,C-18), 28.1 (C-14,C-15); HRESI-MS: *m*/*z* 551.1012 [M + H]^+^ (Calc. for C_25_H_27_O_10_S_2_, 551.1046).

5,6-Dimethoxymacluraxanthone (2d); yellow solid; mp 138.1–141.2 °C; IR *ν*_max_ (cm^−1^): 2966 2917 1648 1596 1458 1428 1286; ^1^H NMR (400 MHz, CDCl_3_) *δ* 13.63 (1H, s, 1-OH), 7.96 (2H, d, *J* = 8.8 Hz, H-8), 6.98 (1H, d, *J* = 8.8 Hz, H-7), 6.73 (2H, d, *J* = 10.0 Hz, H-11), 6.37 (1H, dd, *J* = 17.6, 10.4 Hz, H-19), 5.58 (2H, d, *J* = 10 Hz, H-12), 4.92 (1H, dd, *J* = 17.6, 1.2 Hz, H-20a), 4.84 (1H, dd, *J* = 10.4, 1.2 Hz, H-20b), 4.00 (3H, s, 6-OCH_3_), 3.92 (3H, s, 5-OCH_3_), 1.727 (6H, s, H-17,H-18), 1.47 (6H, s, H-14,H-15); ^13^C NMR (100 MHz, CDCl_3_) *δ* 181.1 (C-9) 159.4 (C-1), 158.1 (C-3), 156.6 (C-4a), 155.4 (C-19), 150.8 (C-6), 150.1 (C-10a), 136.5 (C-5), 127.3 (C-12), 121.4 (C-11), 116.1 (C-8), 114.9 (C-8a), 113.8 (C-7), 108.7 (C-4), 107.9 (C-20), 105.3 (C-2), 103.4 (C-9a), 78.3 (C-13), 61.6 (6-OCH_3_), 56.5 (5-OCH_3_), 41.2 (C-16), 30.0 (C-17,C-18), 27.9 (C-14,C-15); HRESI-MS: *m*/*z* 423.1795 [M + H]^+^ (Calc. for C_25_H_27_O_6_, 423.1808).

5-Methoxymacluraxanthone (2e); yellow crystals; mp 151.4–158.8 °C; IR *ν*_max_ (cm^−1^): 3279 2933 2840 1619 1567 1412 1282; ^1^H NMR (400 MHz, CDCl_3_) *δ* 13.54 (1H, s, 1-OH) 7.73 (1H, d, *J* = 8.8 Hz, H-8), 6.95 (1H, d, *J* = 8.8 Hz, H-7), 6.75 (1H, d, *J* = 10.0 Hz, H-11), 6.65 (1H, dd, *J* = 17.6, 10.4 Hz, H-19), 6.22 (1H, s, 6-OH), 5.60 (1H, d, *J* = 10.0 Hz, H-12), 5.18 (1H, dd, *J* = 17.6, 1.2 Hz, H-20a), 5.04 (1H, dd, *J* = 10.4, 1.2 Hz, H-20b), 4.01 (3H, s, 5-OCH_3_), 1.65 (6H, s, H-17,H-18), 1.50 (6H, s, H-14,H-15); ^13^C NMR (100 MHz, CDCl_3_) *δ* 181.1 (C-9), 159.2 (C-1), 156.8 (C-3), 155.1 (C-4a), 154.6 (C-19), 151.6 (C-6), 144.5 (C-10a), 133.6 (C-5), 127.3 (C-12), 116.9 (C-11), 116.1 (C-8), 114.4 (C-8a), 113.5 (C-7), 108.5 (C-4), 105.5 (C-20), 104.8 (C-2), 103.2 (C-9a), 78.4 (C-13), 56.7 (5-OCH_3_), 41.4 (C-16), 28.6 (C-17,C-18), 28.0 (C-14,C-15); HRESI-MS: *m*/*z* 409.1657 [M + H]^+^ (Calc. for C_24_H_25_O_6_ 409.1651).

5,6-Diethoxymacluraxanthone (2f); yellow solid; mp 118.6–120.3 °C; IR *ν*_max_ (cm^−1^): 3599 2979 1644 1594 1571 1446 1287; ^1^H NMR (400 MHz, CDCl_3_) *δ* 13.68 (1H, s, 1-OH), 7.91 (1H, d, *J* = 8.8 Hz, H-8), 6.94 (1H, d, *J* = 8.8 Hz, H-7), 6.74 (1H, d, *J* = 10.0 Hz, H-11), 6.38 (1H, dd, *J* = 17.6, 10.4 Hz, H-19), 5.57 (1H, d, *J* = 10.0 Hz, H-12), 4.90 (1H, dd, *J* = 17.6, 1.2 Hz, H-20a), 4.83 (1H, dd, *J* = 10.4, 1.2 Hz, H-20b), 4.20 (2H, m, H-1′′), 4.15 (2H, m, H-1′), 1.73 (6H, s, H-18,17), 1.47 (12H, m, 2′′,2′,14,15); ^13^C NMR (100 MHz, CDCl_3_) *δ* 181.1 (C-9), 159.3 (C-1), 157.5 (C-3), 156.6 (C-4a), 155.5 (C-19), 150.9 (C-6), 150.3 (C-10a), 135.5 (C-5), 127.3 (C-12), 121.1 (C-11), 116.1 (C-8), 114.7 (C-8a), 113.7 (C-7), 109.5 (C-4), 107.8 (C-20), 105.2 (C-2), 103.4 (C-9a), 78.2 (C-13), 69.5 (C-1′′), 64.9 (C-1′), 41.2 (C-16), 30.0 (C-17,C-18), 27.9 (C-14,C-15), 15.6 (C-2′′), 14.9 (C-2′); HRESI-MS: *m*/*z* 451.2121 [M + H]^+^ (Calc. for C_27_H_31_O_6_, 451.2121).

5-Ethoxymacluraxanthone (2g); yellow solid; mp 126.3–128.6 °C; IR *ν*_max_ (cm^−1^): 3272 2964 1647 1619 1594 1568 1460 1417 1139; ^1^H NMR (400 MHz, CDCl_3_) *δ* 13.57 (1H, s, 1-OH), 7.71 (1H, d, *J* = 8.8 Hz, H-8), 6.94 (1H, d, *J* = 8.8 Hz, H-7), 6.76 (1H, d, *J* = 10.0 Hz, H-11), 6.63 (1H, dd, *J* = 17.6, 10.4 Hz, H-19), 6.18 (1H, s, 6-OH), 5.60 (1H, d, *J* = 10.0 Hz, H-12), 5.16 (1H, dd, *J* = 17.6, 1.2 Hz, H-20a), 5.04 (1H, dd, *J* = 10.4, 1.2 Hz, H-20b), 4.25 (2H, m, H-1′′), 1.66 (6H, s, H-17,18), 1.52 (3H, d, H-2′′), 1.50 (6H, s, 14,15); ^13^C NMR (100 MHz, CDCl_3_) *δ* 181.1 (C-9), 159.2 (C-1), 156.8 (C-3), 154.7 (C-4a), 154.6 (C-19), 151.0 (C-6), 144.6 (C-10a), 133.7 (C-5), 127.2 (C-12), 116.8 (C-11), 116.2 (C-8), 114.3 (C-8a), 113.5 (C-7), 109.4 (C-4), 105.5 (C-20), 105.1 (C-2), 103.3 (C-9a), 78.3 (C-13), 65.2 (C-1′′), 41.4 (C-16), 28.7 (C-17,C-18), 28.0 (C-14,C-15), 14.9 (C-2′′); HRESI-MS: *m*/*z* 423.1808 [M + H]^+^ (Calc. for C_25_H_27_O_6_, 423.1808).

### 3.5 Cytotoxicity and cell proliferation assays

All types of cancer and non-tumorigenic cells were purchased from ATCC. Cytotoxicity assays against human cervical carcinoma (HelaS3; ATCC CCL2-2), human hepatocellular carcinoma (HepG2; ATCC HB-8065), human lung carcinoma (A549; ATCC CCL-185), and African green monkey kidney (Vero; ATCC CCL-81) cell lines were performed by terminal deoxynucleotidyl transferase (TdT) dUTP Nick-End Labeling (TUNEL) assay.^44^ Cells proliferation was performed using the MTT colorimetric assay,^44^ as in our previous publication.^4^

### 3.6 Antibacterial assay

Four bacterial cultures *Bacillus cereus* ATCC 11778, *Bacillus subtilis* ATCC 6633, *Staphylococcus aureus* ATCC 25923, and Methicillin resistant *Staphylococcus aureus* (MRSA) were employed as the test organisms. Minimum inhibitory concentration (MIC) values were determined using the dilution method, as described in the M07-A9,^45^ as described in our previous paper.^4,46^

### 3.7 Molecular docking simulation

All the calculations were performed on Intel® Core™ i7-12700F CPU 2.11 GHz with 32 GB DDR4 RAM. The crystallographic structures of cyclin-dependent kinase 2 (CDK2) in complex with the inhibitor RC-3-89 (PDB code: 4GCJ), methicillin acyl-penicillin binding protein 2a from methicillin resistant *Staphylococcus aureus* (PDB code: 1MWU), and *Bacillus cereus* PatB1 (PDB code: 5V8E) were obtained from the Protein Data Bank. The existing ligands and water molecules were removed from the crystal structure. Molecular docking studies were performed using the AutoDock 4.2.6 program.^47^ The 3D structures of compounds 1, 1a, 1b, and 1f were minimized at the B3LYP/6-31G(d) levels using the Gaussian 03 program. All hydrogens were added and Gasteiger charges were assigned by using AutoDockTools.^47^ A grid box of 40 × 40 × 40 points was centered at the coordinates of the RC-3-89 inhibitor (*x* = −51.563, *y* = 98.287, *z* = −62.755) to define the binding site of the CDK2 protein. Similarly, the grid box was generated at the center of the coordinate's ligand with dimensions of 40 × 40 × 40 points, centered at *x* = 28.221, *y* = 28.839, and *z* = 87.515 for PBP2a from MRSA, and centered at *x* = −67.474, *y* = −1.104, and *z* = −15.158 for *Bacillus cereus* PatB1. For *B. subtilis* TagU, a grid box was generated in the absence of a co-crystallized ligand to cover all active site residues^35^ with dimensions of 70 × 70 × 70 points, centered at *x* = 8.698, *y* = 24.542, and *z* = 24.418. All grid boxes were constructed with a grid spacing of 0.375 Å. The optimized ligands were docked onto protein templates using the Lamarkian genetic algorithm. The docking protocol was set to default, with 150 independent docking runs. The protein templates of CDK2, PBP2a from MRSA, and SCWP *O*-acetyltransferase of *B. cereus* exhibited the best results of redocking experiments with the RMSD of 0.68, 1.67, and 1.42 Å, respectively (Table S1). Finally, the docking results were then analyzed to identify the best cluster of each compound with the lowest free binding energy. Protein–ligand interactions were visualized by using the Discovery Studio 2021 Client program (Acceryls, Inc.,San Diego, CA, USA).

### 3.8 *In silico* physicochemical properties

The physicochemical properties of all compounds were evaluated to assess their potential as drug candidates using the SwissADME^48^ web server. Drug-likeness was verified using the Lipinski's and Veber's rules.

## 4 Conclusions

Two main compounds, toxyloxanthone C (1) and macluraxanthone (2) from *M. cochinchinensis* roots, have been structurally modified through simple organic reactions including acylation, alkylation, sulfonylation, and bromination yielding thirteen derivatives 1a–f and 2a–g. All compounds were evaluated for their cytotoxic and antibacterial activities. Compounds 1a, 1b, and 1f exhibited greater cytotoxic potency against A549 cells compared to their parent compound. Molecular docking studies revealed that 1a, 1b, and 1f bound to CDK2 through hydrogen bonding and hydrophobic interactions with key amino acid residues. Furthermore, 1a and 1b demonstrated lower binding energies than 1. Notably, 1a exhibited potential as a lead anticancer candidate for further development due to its strong cytotoxicity with minimal toxicity toward normal cells. Additionally, 1a, 1b, 1f, and 2a displayed stronger antibacterial activity against four bacterial strains than their parent compounds. The docking results indicated that 1a and 1f interacted with key amino acids in the PBP2a active site of MRSA. In addition, the binding conformation of 1a in the active site of LCP enzyme from *B. subtilis* aligned with that of tetracycline. While 1a, 1f, and tetracycline exhibited similar binding orientations with the key amino acids within the active site of *B. cereus*. The physicochemical properties of 1a and 1f satisfied Lipinski's and Veber's rules, supporting their potential as drug-like candidates. Most derivatives of 2 exhibited reduced anticancer and antibacterial activities compared to the parent xanthone. This decrease in activity is likely due to steric hindrance between the prenyl group at the C-4 position and the additional substituents introduced in the derivatives, a structural effect consistent with previous reports on modified prenylated xanthones.^10,27^ Overall, 1a and 1f exhibited significant dual bioactivities, showing cytotoxicity against A549 cells and antibacterial activity against four bacterial cultures; however, 1f also displayed cytotoxicity toward Vero cells. Therefore, 5,6-diacetoxytoxyloxanthone C (1a) is interesting candidate for further study as anticancer and antibacterial agents.

## Author contributions

C. Linphosan performed the experiments, analyzed the data, evaluated antibacterial activity, and drafted the initial manuscript. W. Klangsawad isolated parent compounds. J. Yahuafai performed cytotoxicity assay. J. Jandaruang and T. Promgool evaluated antibacterial activity. S. Pitchuanchom supervised the molecular docking studies. J. Paluka and S. Boonlue provided antibacterial testing facilities. K. Poopasit carried out the NMR experiment. K. Kanokmedhakul to consultant and manuscript editing. O. Limtragool, the principal investigator, designed the experiments, analyzed the data, conducted *in silico* studies, and prepared the manuscript.

## Conflicts of interest

The authors declare no conflict of interest.

## Supplementary Material

RA-015-D5RA05758B-s001

## Data Availability

The datasets supporting this article have been uploaded as part of the SI. See DOI: https://doi.org/10.1039/d5ra05758b.
