# Supplementary material for: Structural modifications of toxyloxanthone C and macluraxanthone isolated from Maclura cochinchinensis: cytotoxicity, antibacterial activity, and in silico studies
Source: RSC Adv. 2025 Sep 17;15(40):33816–29. doi: 10.1039/d5ra05758b (PMC12441791; doi:10.1039/d5ra05758b)
Supplement: RA-015-D5RA05758B-s001 [file RA-015-D5RA05758B-s001.pdf]

Supplementary Information (SI) for RSC Advances.
This journal is © The Royal Society of Chemistry 2025

Supplementary Materials

Structural modifications of toxyloxanthone C and macluraxanthone isolated from *Maclura cochinchinensis*: cytotoxicity, antibacterial activity, and *in silico* studies

Chaiwat Linphosan,[1] Waranya Klangsawad,[2] Jantana Yahuafai,[3] Jinda Jandaruang,[4] Trinop Promgool,[5] Siripit Pitchuanchom,[1] Jakkapat Paluka,[6] Sophon Boonlue,[7] Kitisak Poopasit,[2] Kwanjai Kanokmedhakul,[2] Oue-artorn Limtragool*,[1]

[1]Multidisciplinary Research Unit of Pure and Applied Chemistry, Department of Chemistry and Center of Excellence for Innovation in Chemistry, Faculty of Science, Mahasarakham University, Maha Sarakham, Thailand.

[2]Department of Chemistry and Center of Excellence for Innovation in Chemistry, Faculty of Science, Khon Kaen University, Khon Kaen, Thailand.

[3]Clinical Research Section, Division of Research and Academic Support, National Cancer Institute, Bangkok, Thailand.

[4]Innovation in Chemistry for Community Research Unit, Chemistry program, Faculty of Science and Technology, Sakon Nakhon Rajabhat University, Sakon Nakhon, Thailand.

[5]Department of Industrial Chemistry, Faculty of Applied Science, King Mongkut's University of Technology North Bangkok, Bangkok, Thailand.

[6]Research Administration Division, Khon Kaen University, Khon Kaen, Thailand.

[7]Department of Microbiology, Faculty of Science, Khon Kaen University, Khon Kaen, Thailand

*Corresponding author

*Tel.: +6643-754246. Fax: +6643-754246. E-mail address: oueartorn.l@msu.ac.th

Table S1 Redock experiments.

Compound	Binding energy (kcal/mol)	Protein-ligand interactions
Reference ligand with PBP2a of MRSA		
Redock (RMSD 1.67)	-7.73	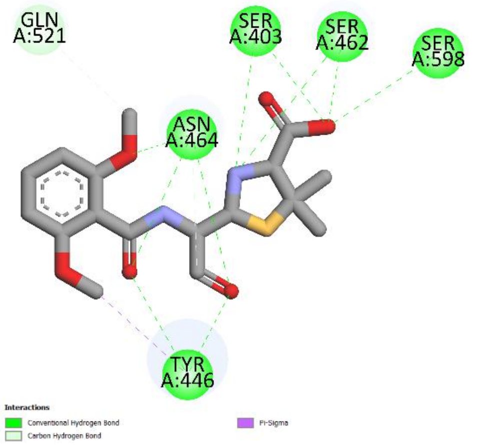
Reference ligand with SCWP O-acetyltransferase of *B. cereus*		
Redock (RMSD 1.42)	-4.24	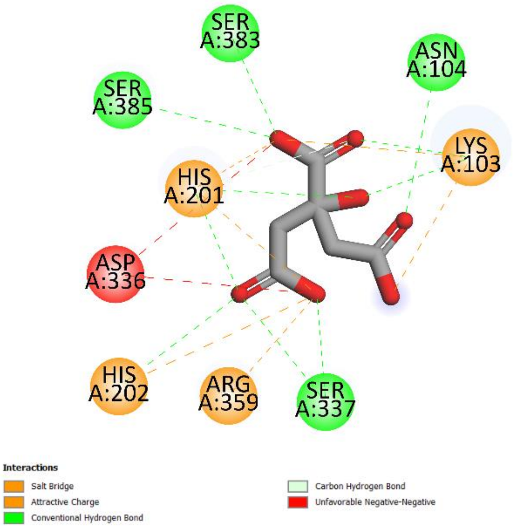

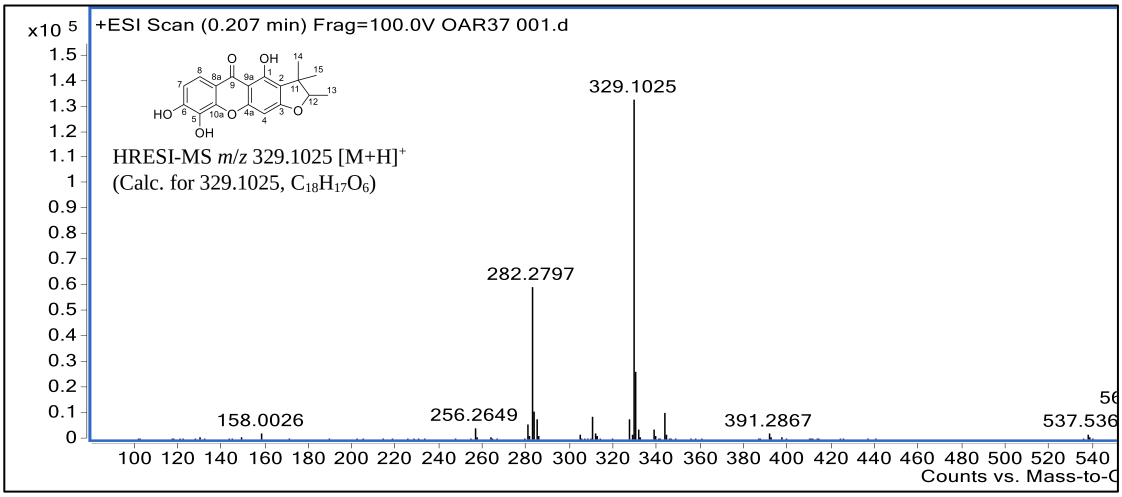

Figure S1 MS spectrum of toxyloxanthone C (**1**)

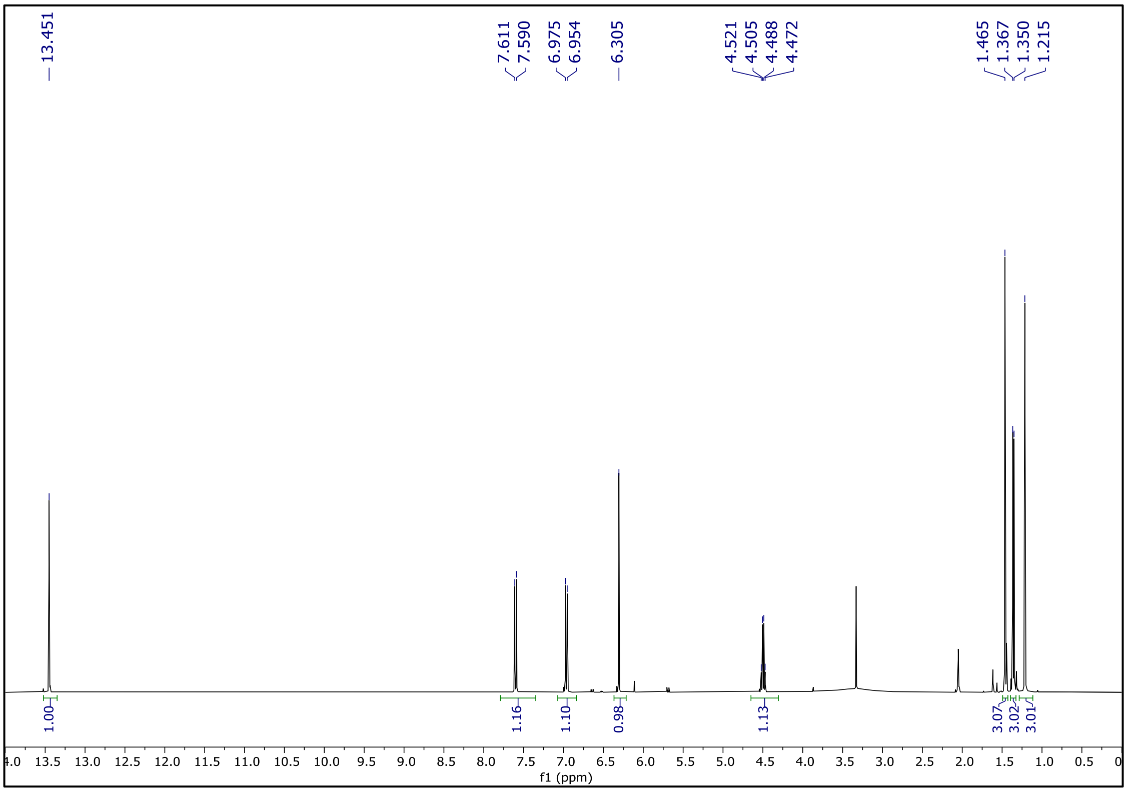

Figure S2 ^{1}H NMR spectrum of toxyloxanthone C (**1**) in CD_3COCD_3

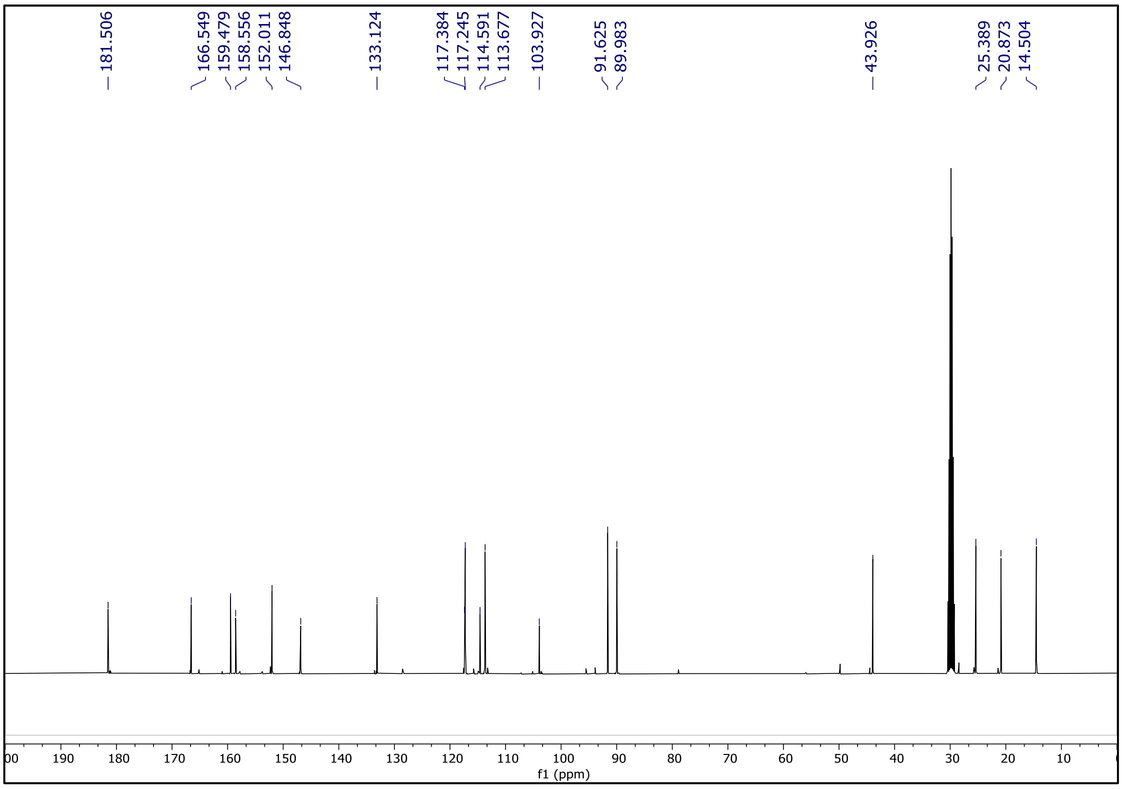

Figure S3 ^{13}C NMR spectrum of toxyloxanthone C (**1**) in CD_3COCD_3

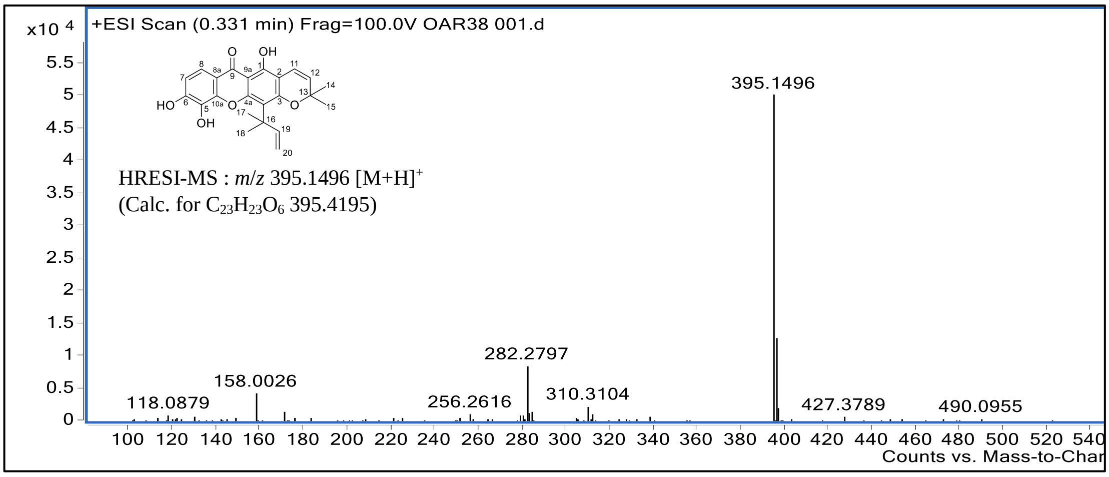

Figure S4 MS spectrum of macluraxanthone (**2**)

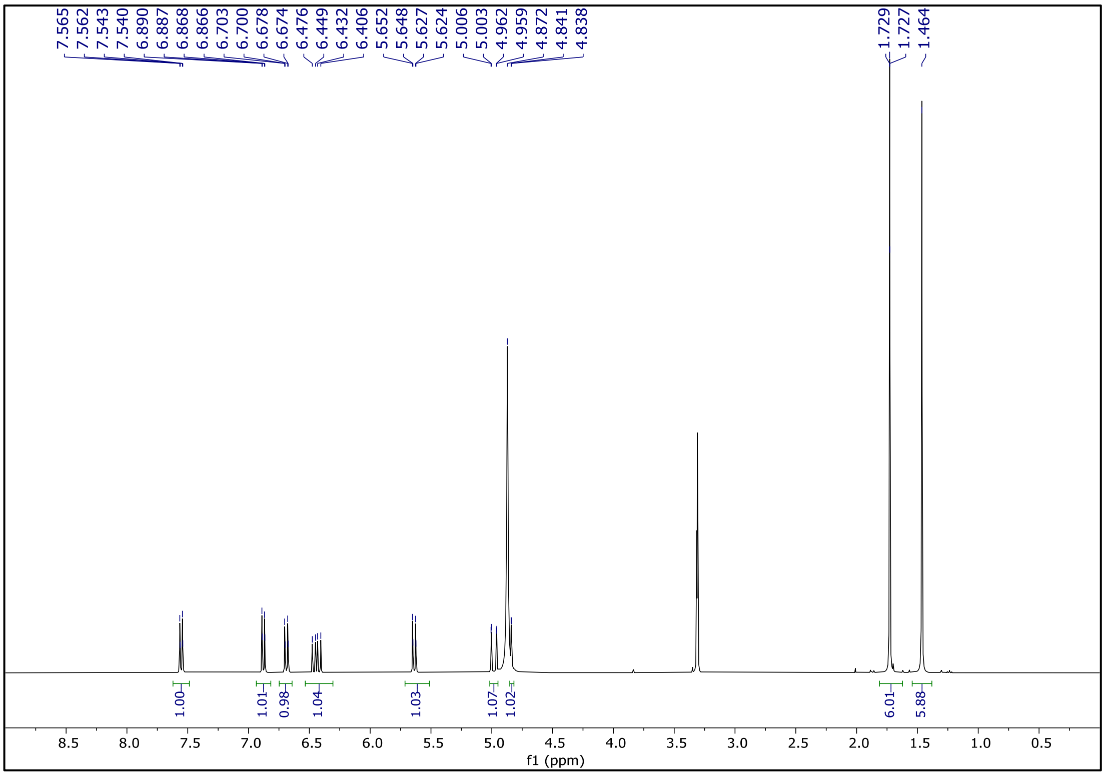

Figure S5 ^{1}H NMR spectrum of macluraxanthone (**2**) in CD_3OD

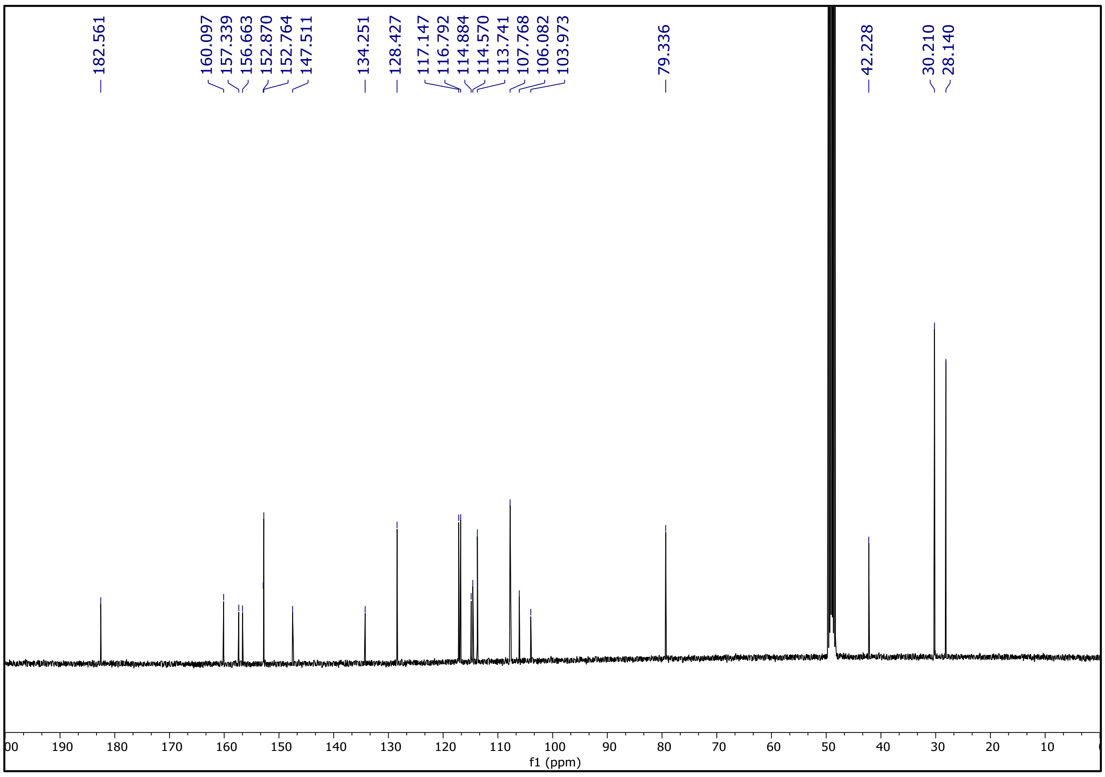

Figure S6 ^{13}C NMR spectrum of macluraxanthone (**2**) in CD_3OD

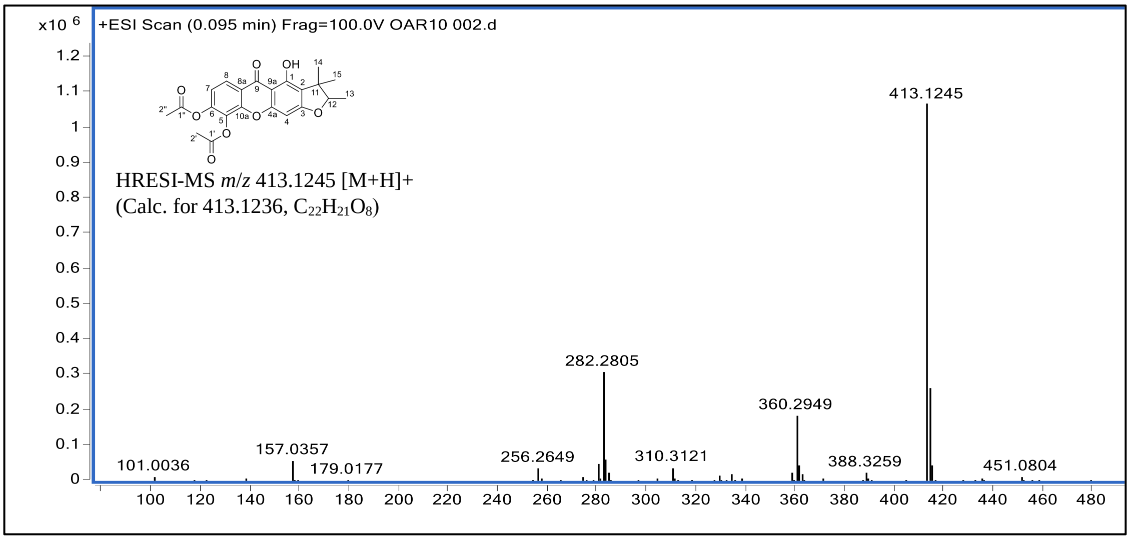

Figure S7 MS spectrum of 5,6-diacetoxytoxyloxanthone C (**1a**)

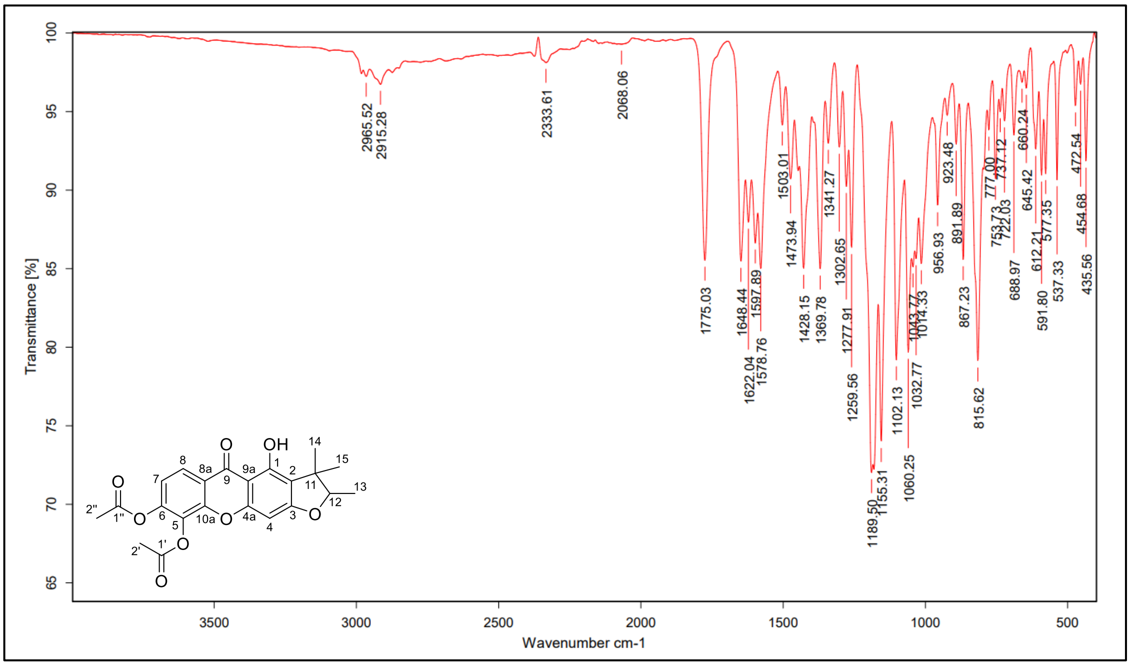

Figure S8 IR spectrum of 5,6-diacetoxytoxyloxanthone C (**1a**)

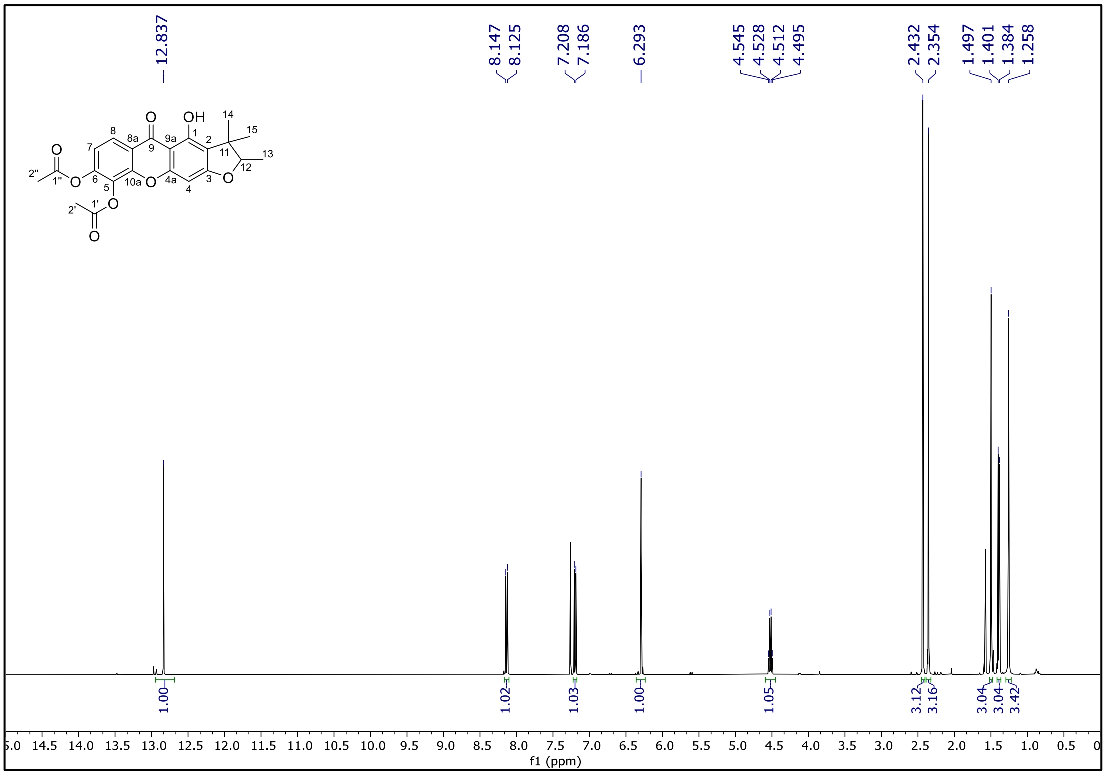

Figure S9 ^{1}H NMR spectrum of 5,6-diacetoxytoxyloxanthone C (**1a**) in $CDCl_3$

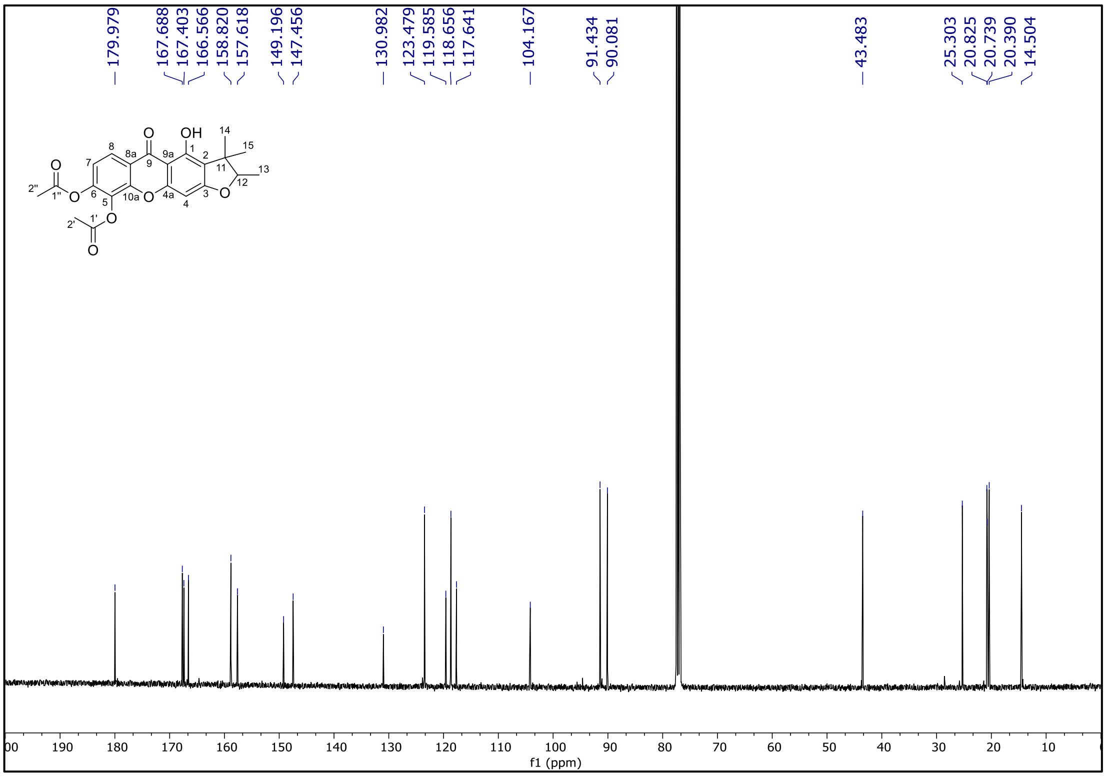

Figure S10 ^{13}C NMR spectrum of 5,6-diacetoxytoxyloxanthone C (**1a**) in $CDCl_3$

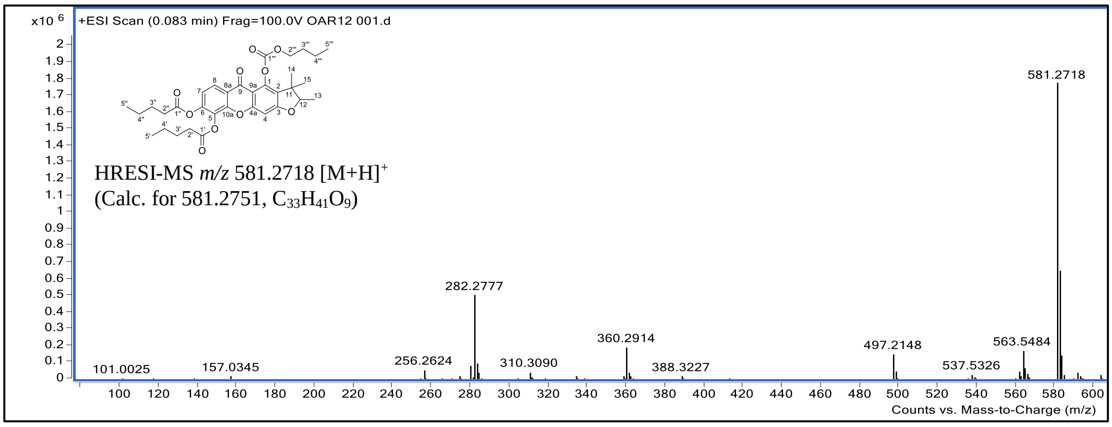

Figure S11 MS spectrum of 1,5,6-tripentanoyloxytoxyloxanthone C (**1b**)

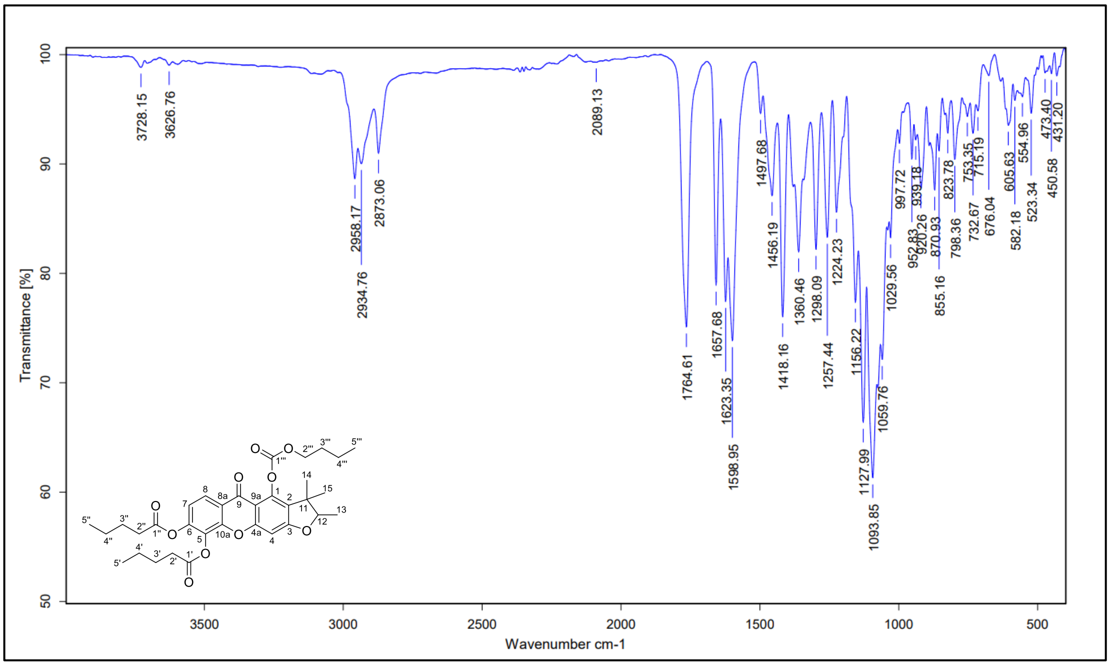

Figure S12 IR spectrum of 1,5,6-tripentanoyloxytoxyloxanthone C (**1b**)

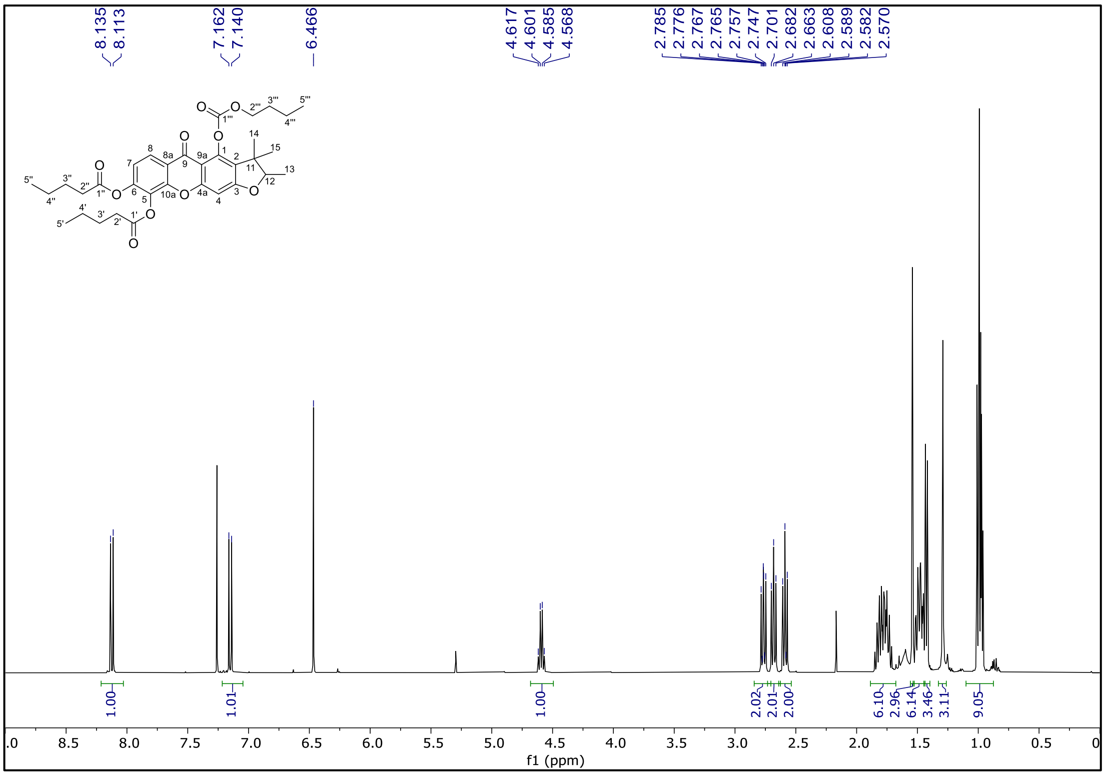

Figure S13 ^{1}H NMR spectrum of 1,5,6-tripentanoyloxytoxyloxanthone C (**1b**) in $CDCl_3$

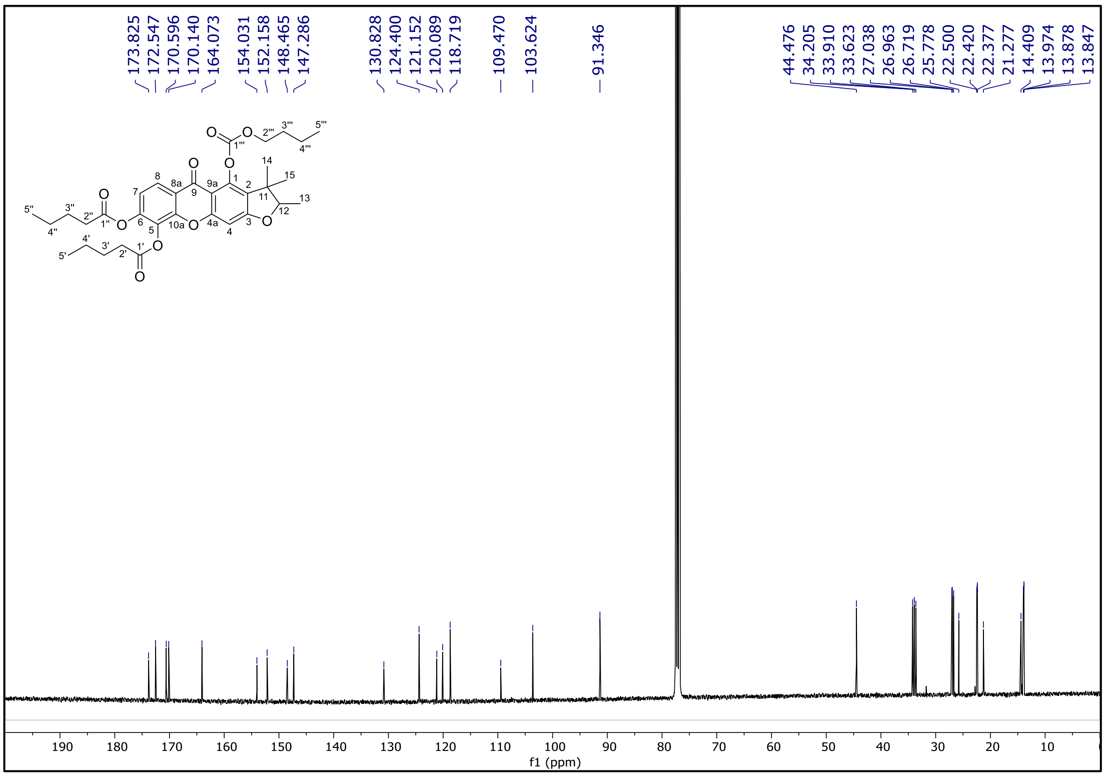

Figure S14 ^{13}C NMR spectrum of 1,5,6-tripentanoyloxytoxyloxanthone C (**1b**) in $CDCl_3$

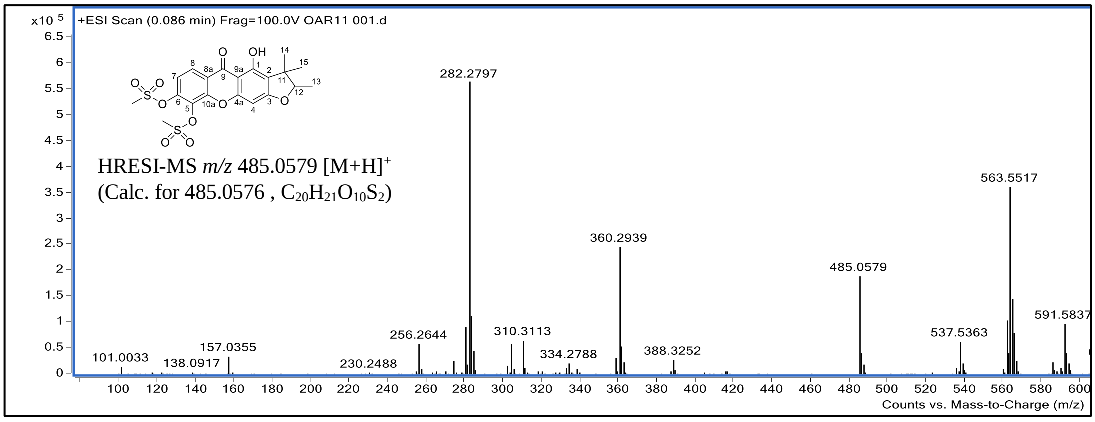

Figure S15 MS spectrum of 5,6-dimesyloxytoxyloxanthone C (**1c**)

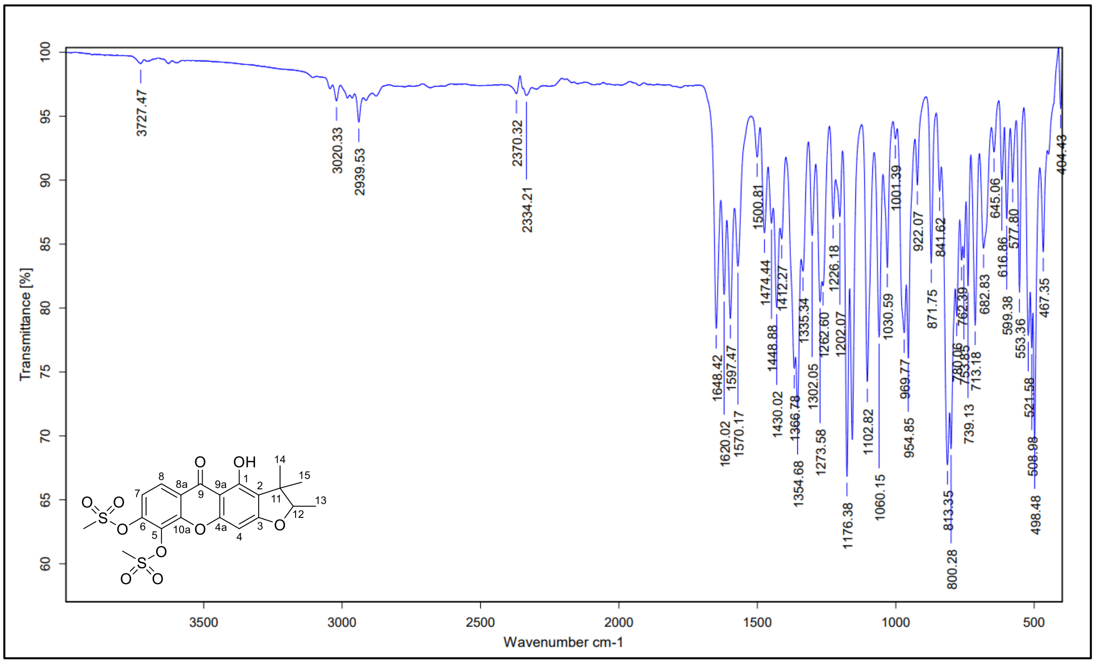

Figure S16 IR spectrum of 5,6-dimesyloxytoxyloxanthone C (**1c**)

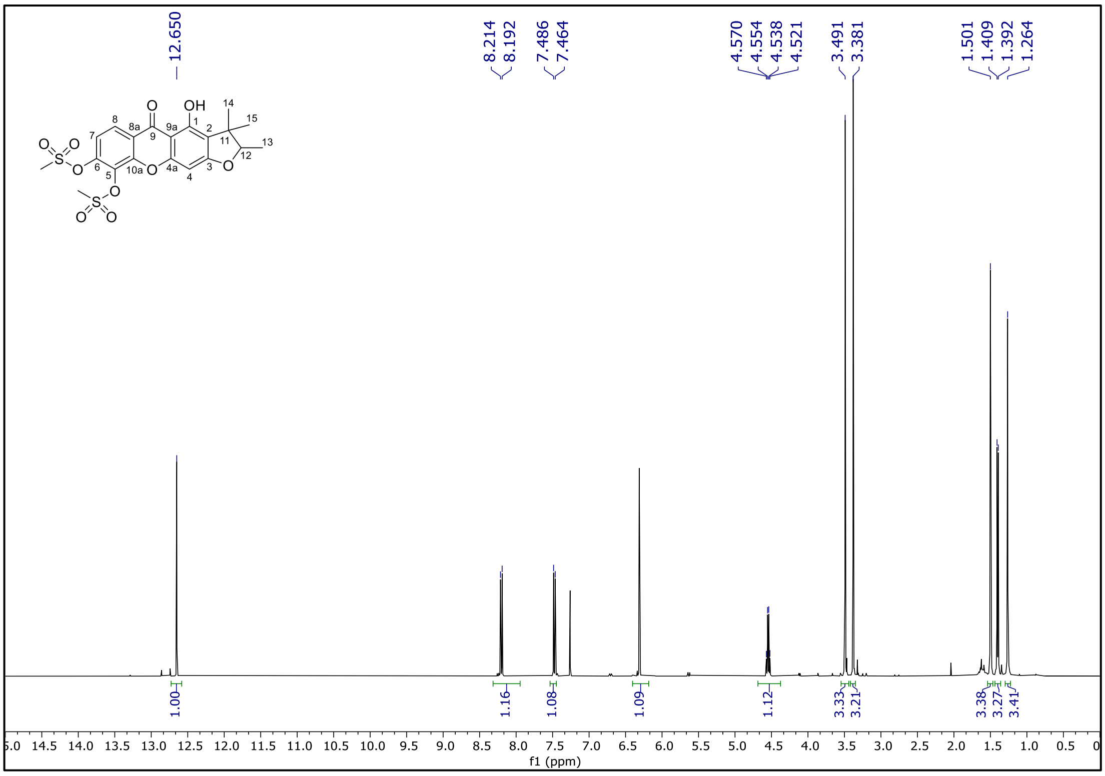

Figure S17 ^{1}H NMR spectrum of 5,6-dimesyloxytoxyloxanthone C (**1c**) in $CDCl_3$

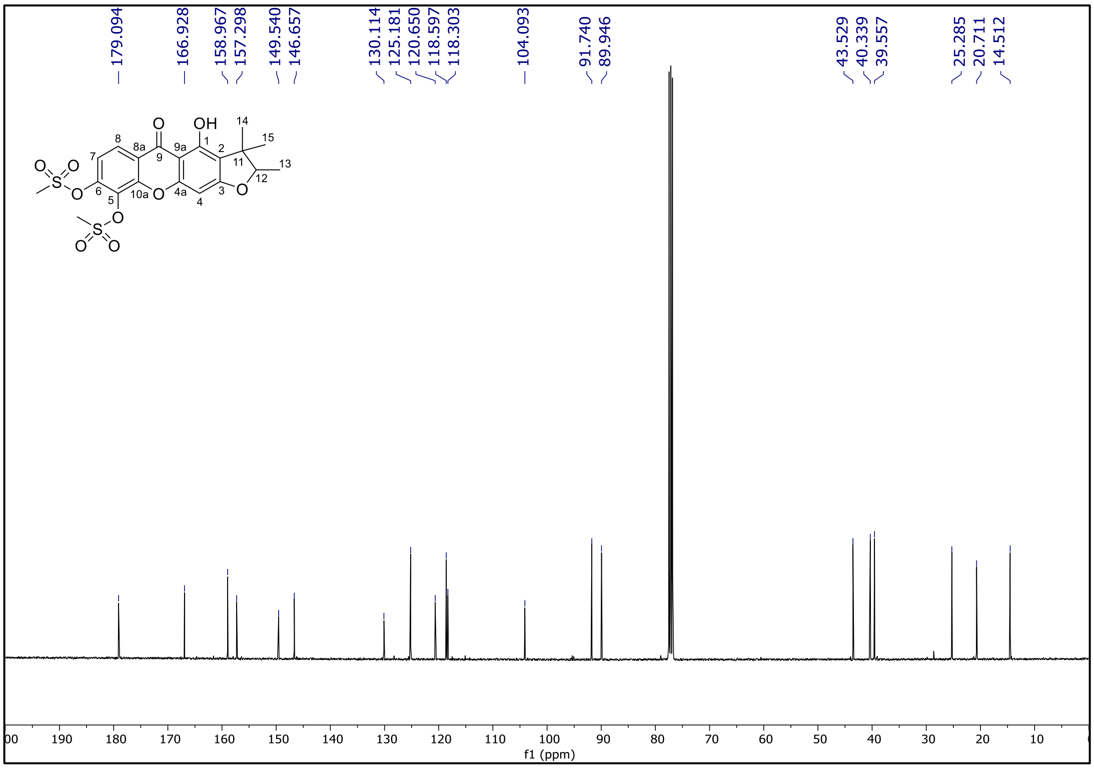

Figure S18 ^{13}C NMR spectrum of 5,6-dimesyloxytoxyloxanthone C (**1c**) in $CDCl_3$

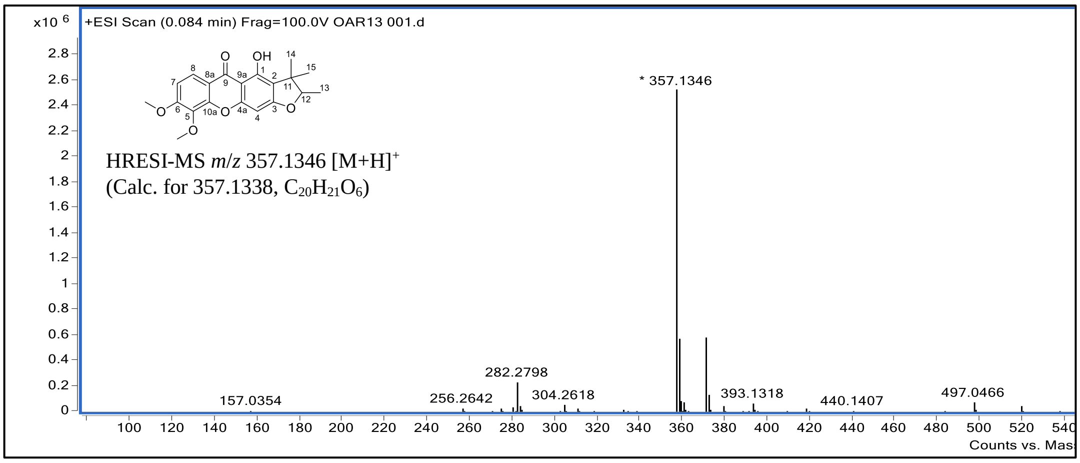

Figure S19 MS spectrum of 5,6-dimethoxytoxyloxanthone C (**1d**)

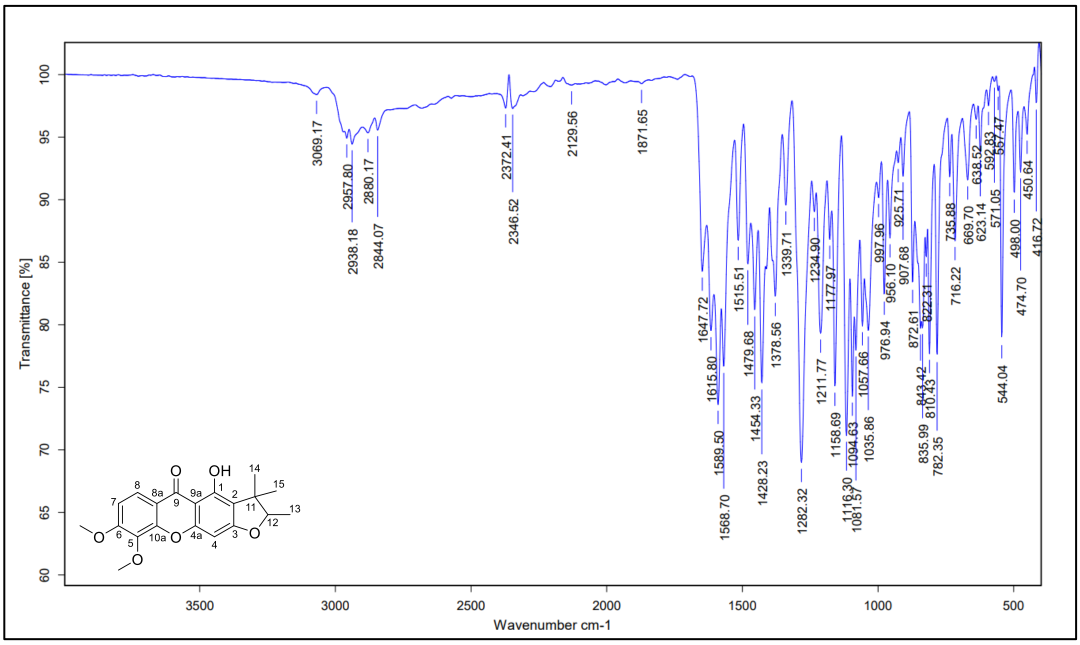

Figure S20 IR spectrum of 5,6-dimethoxytoxyloxanthone C (**1d**)

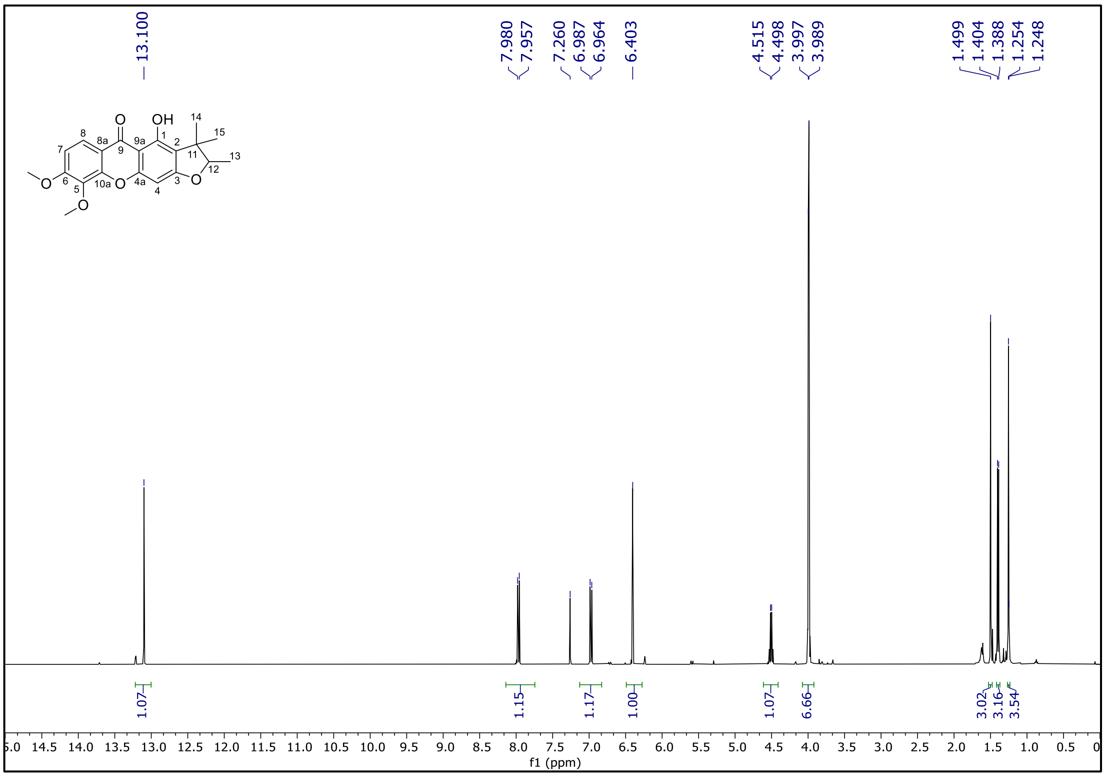

Figure S21 ^{1}H NMR spectrum of 5,6-dimethoxytoxyloxanthone C (**1d**) in $CDCl_3$

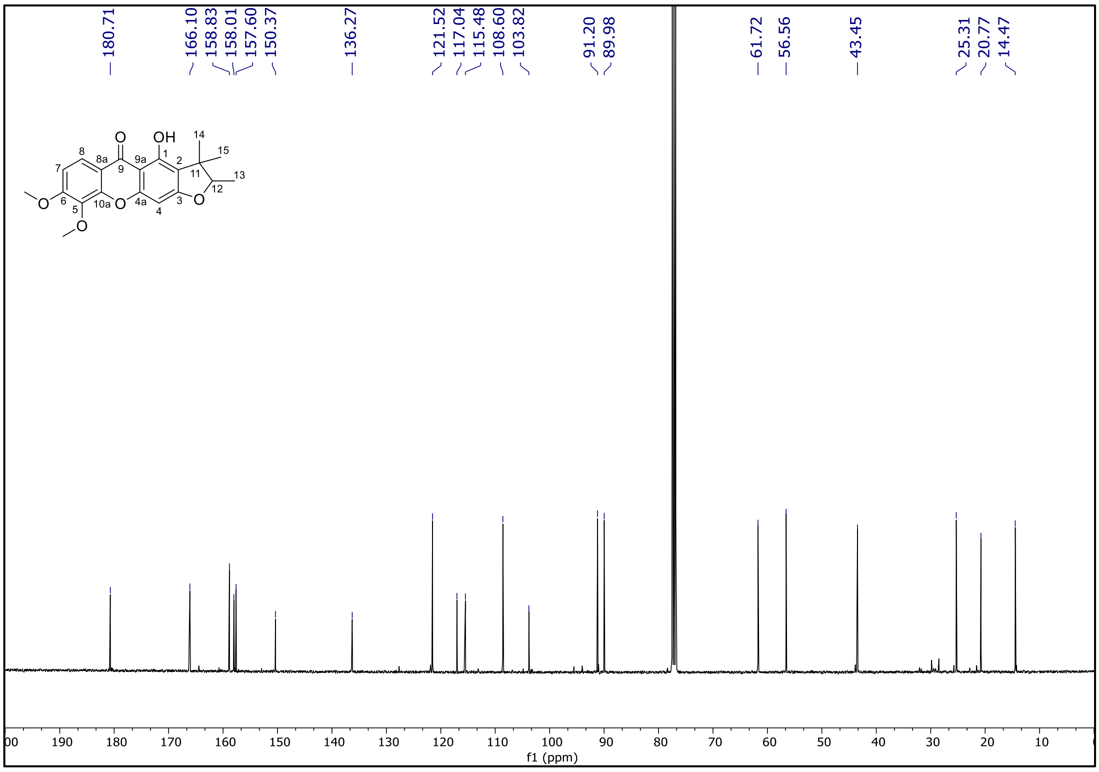

Figure S22 ^{13}C NMR spectrum of 5,6-dimethoxytoxyloxanthone C (**1d**) in $CDCl_3$

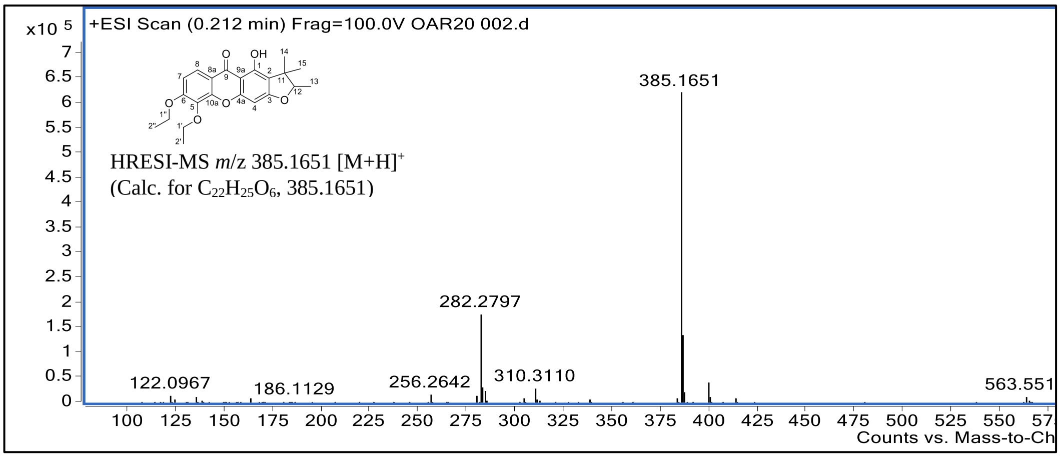

Figure S23 MS spectrum of 5,6-diethoxytoxyloxanthone C (**1e**)

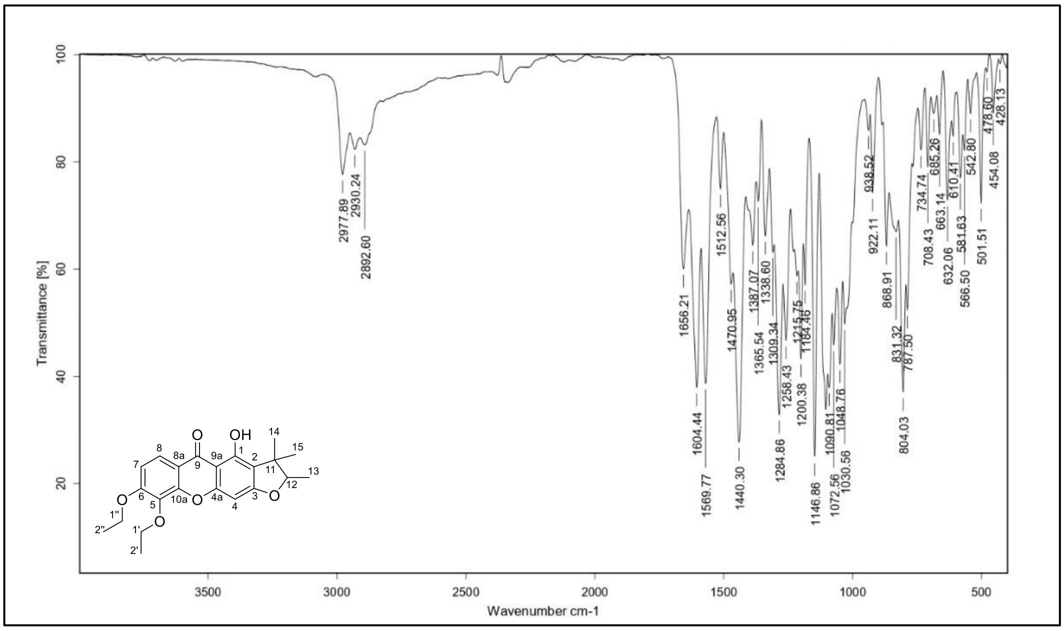

Figure S24 IR spectrum of 5,6-diethoxytoxyloxanthone C (**1e**)

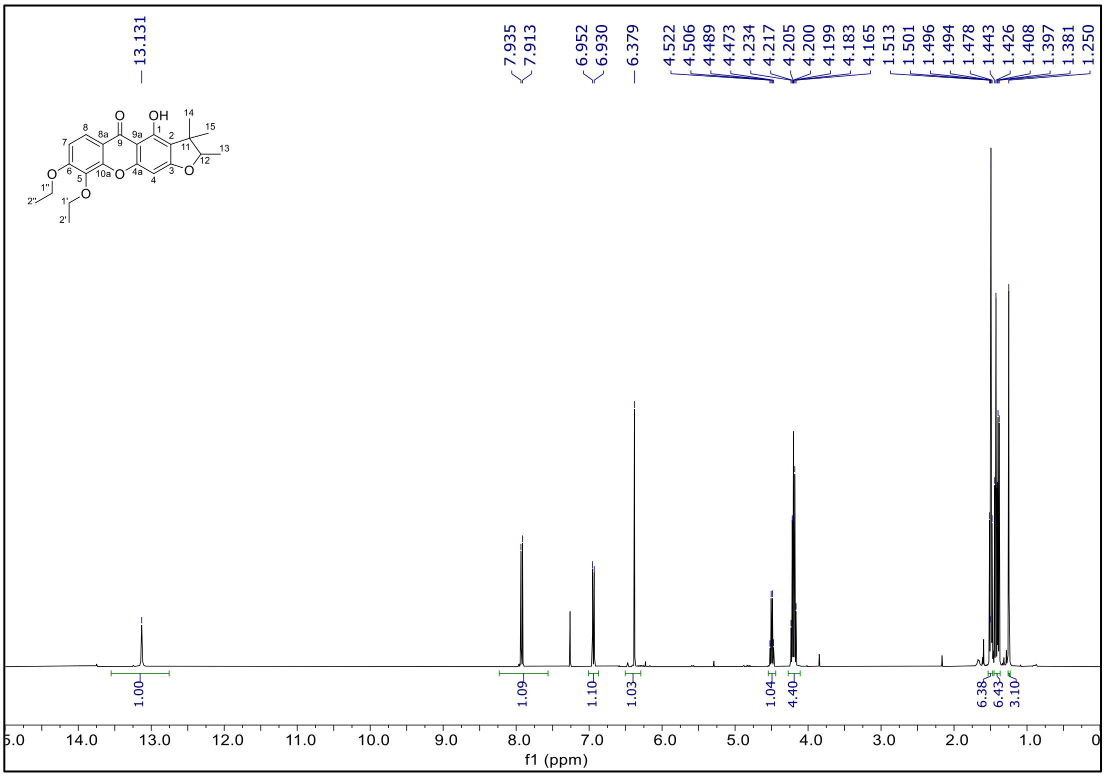

Figure S25 ^{1}H NMR spectrum of 5,6-diethoxytoxyloxanthone C (**1e**) in $CDCl_3$

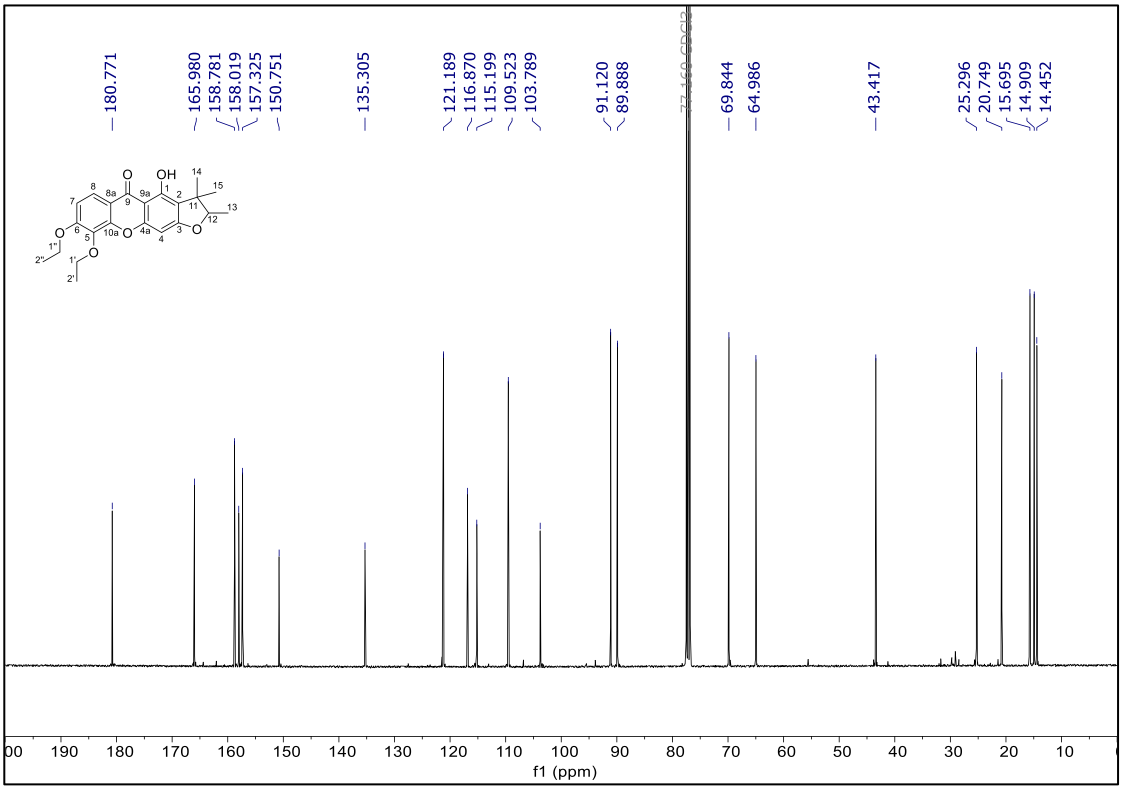

Figure S26 ^{13}C NMR spectrum of 5,6-diethoxytoxyloxanthone C (**1e**) in $CDCl_3$

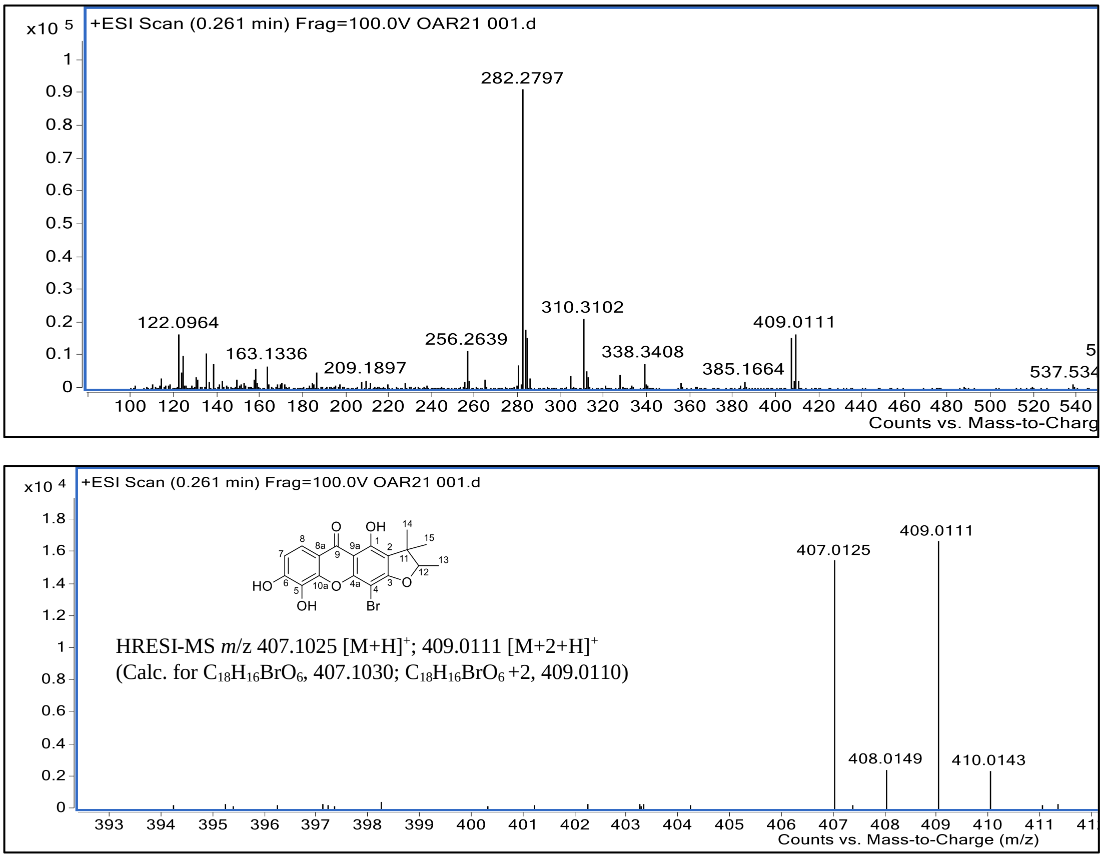

Figure S27 MS spectrum of 4-bromotoxyloxanthone C (**1f**)

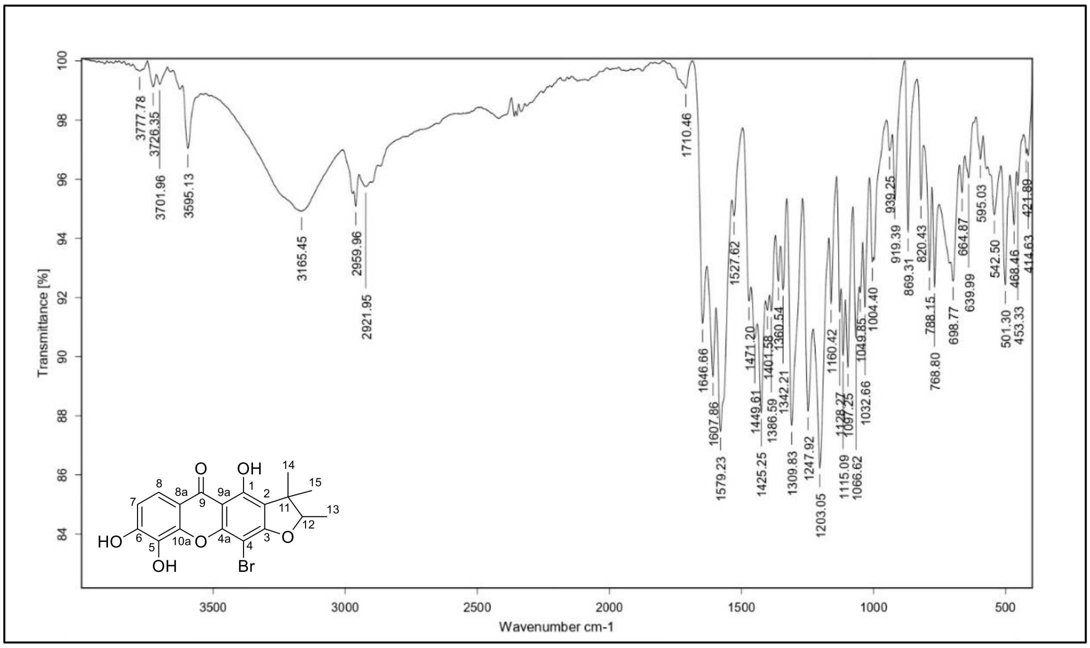

Figure S28 IR spectrum of 4-bromotoxyloxanthone C (**1f**)

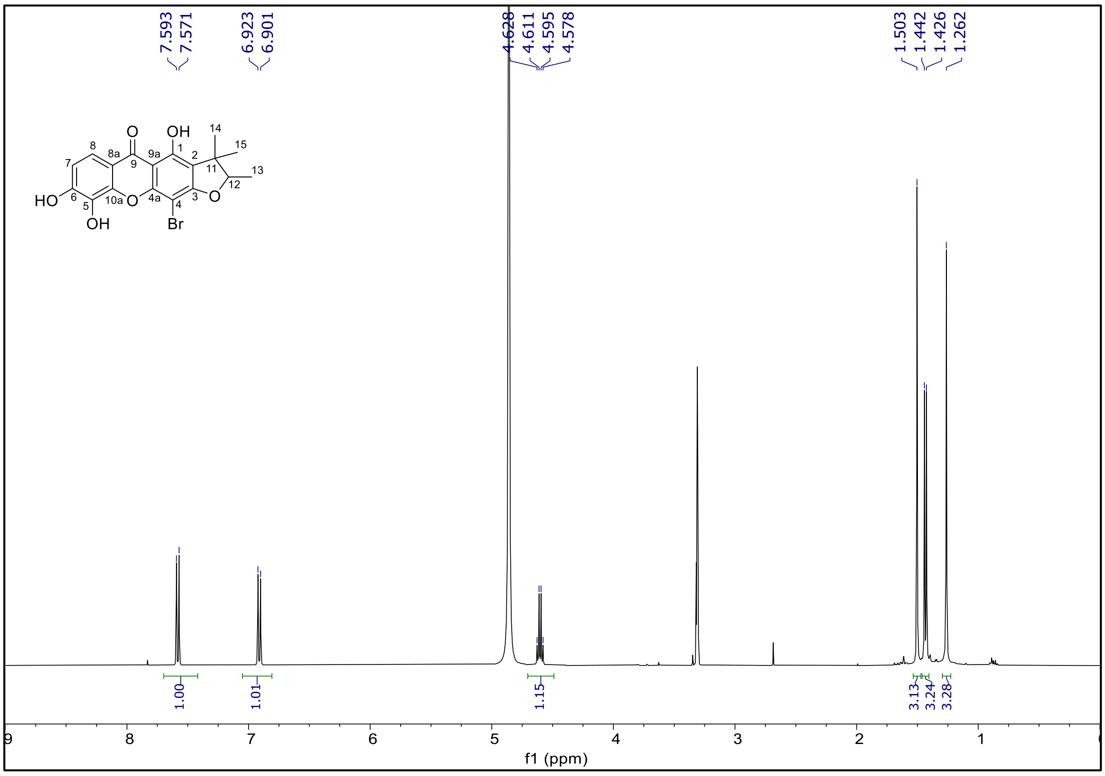

Figure S29 ^{1}H NMR spectrum of 4-bromotoxyloxanthone C (**1f**) in CD_3OD

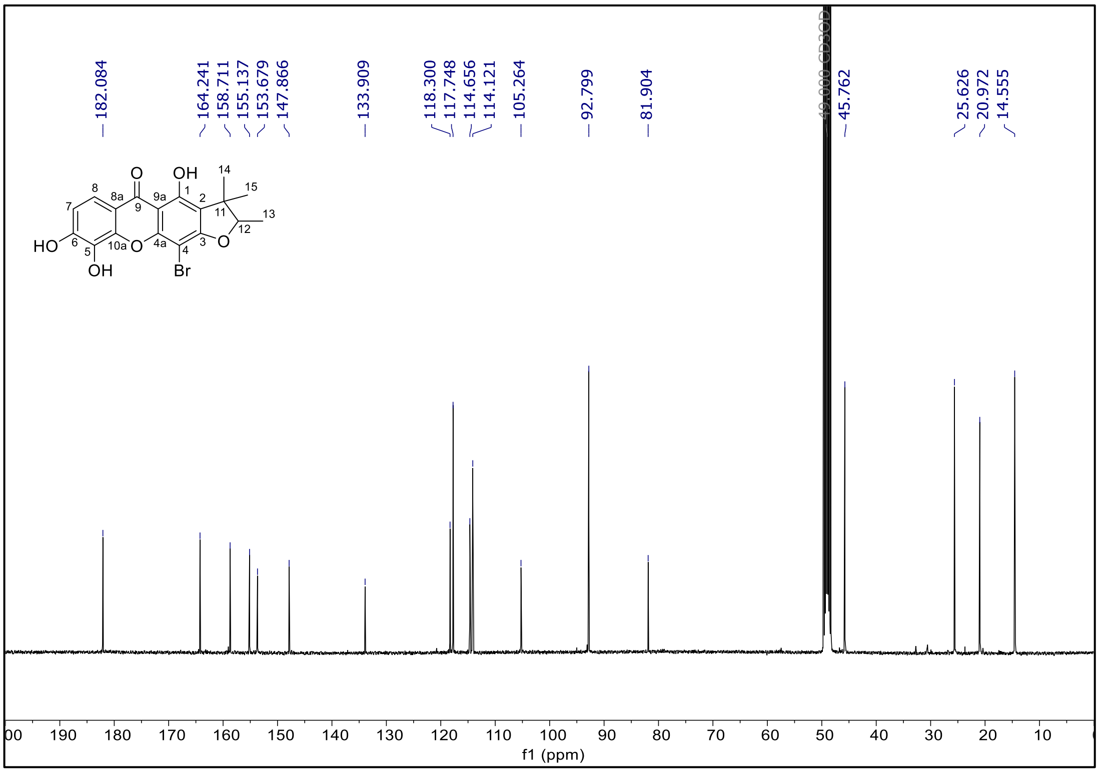

Figure S30 ^{13}C NMR spectrum of 4-bromotoxyloxanthone C (**1f**) in CD_3OD

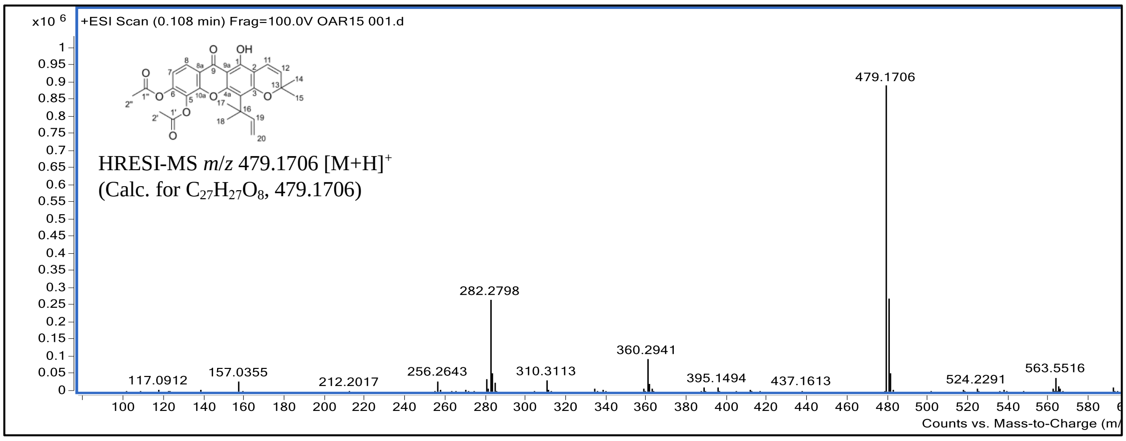

Figure S31 MS spectrum of 5,6-diacetoxymacluraxanthone (**2a**)

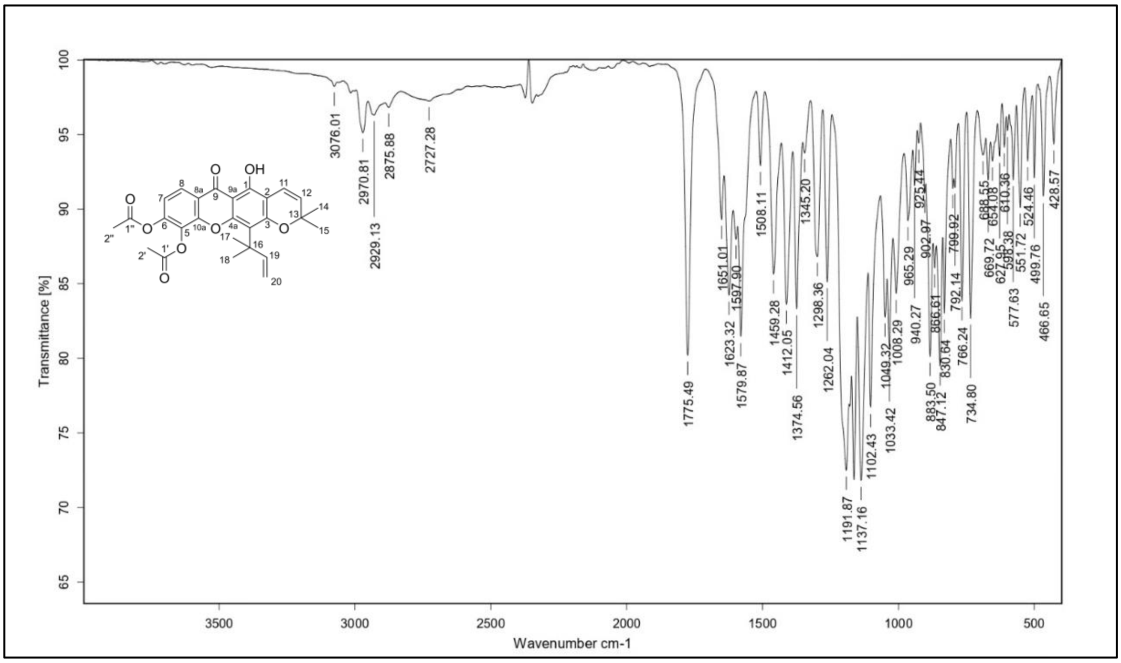

Figure S32 IR spectrum of 5,6-diacetoxymacluraxanthone (**2a**)

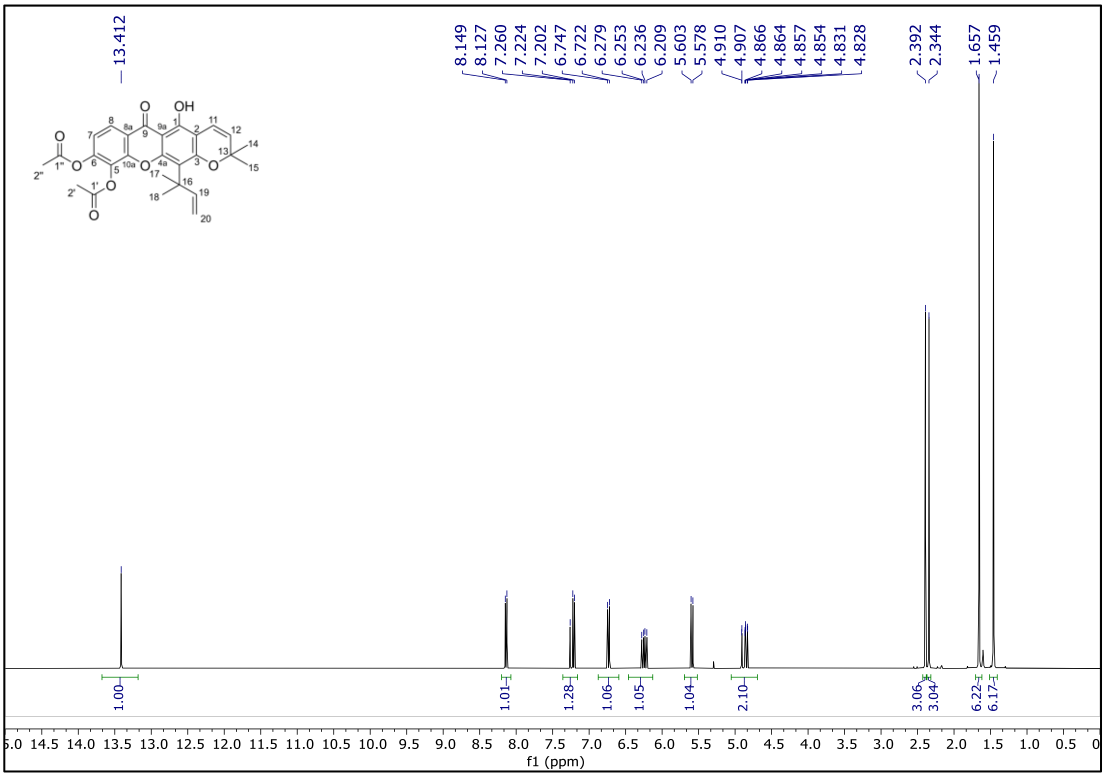

Figure S33 ^{1}H NMR spectrum of 5,6-diacetoxymacluraxanthone (**2a**) in $CDCl_3$

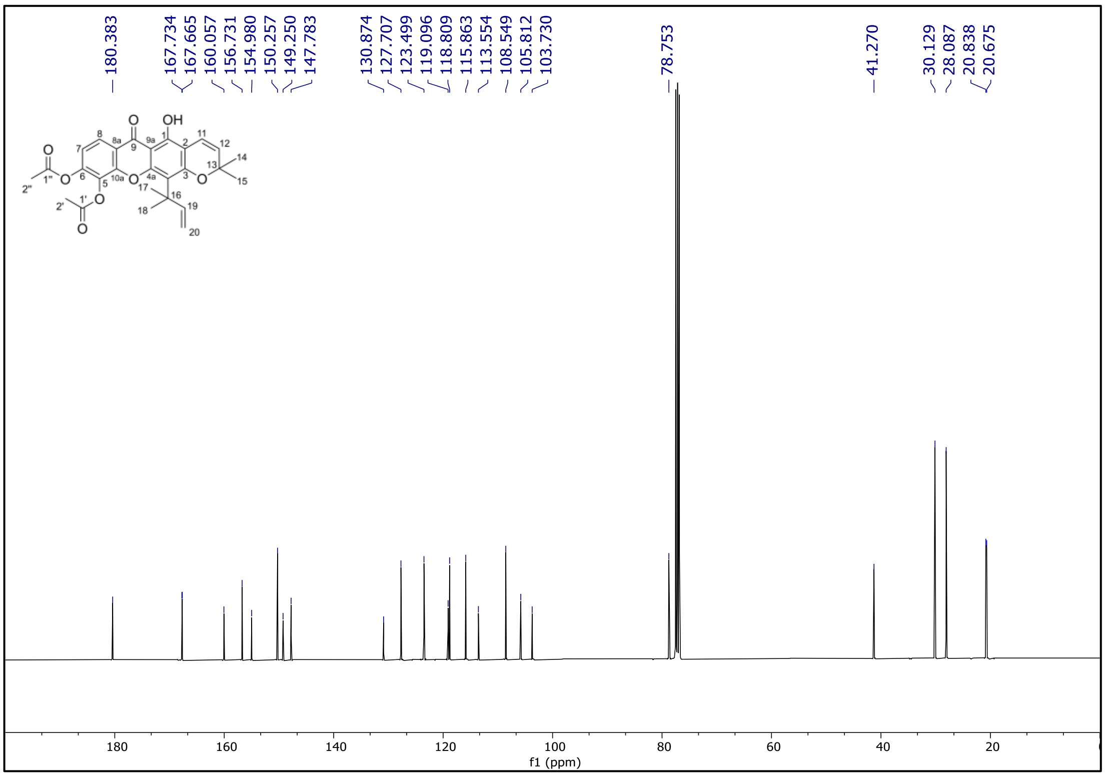

Figure S34 ^{13}C NMR spectrum of 5,6-diacetoxymacluraxanthone (**2a**) in $CDCl_3$

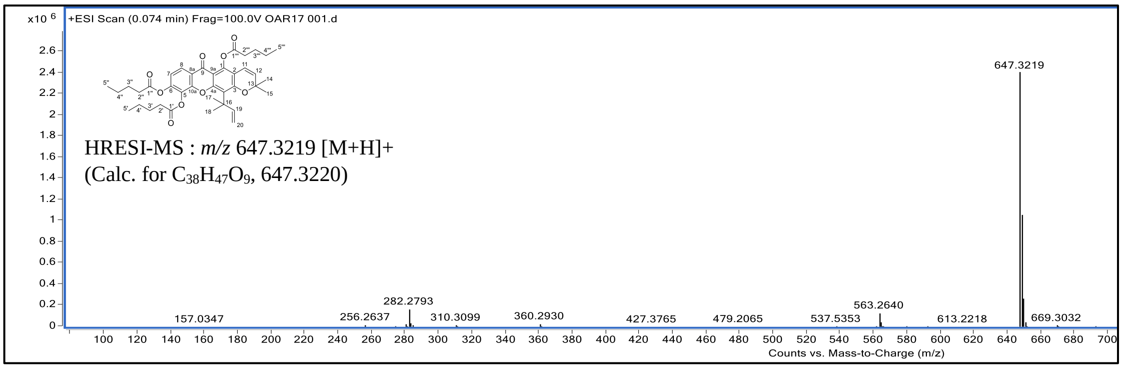

Figure S35 MS spectrum of 1,5,6-tripentanoyloxymacluraxanthone (**2b**)

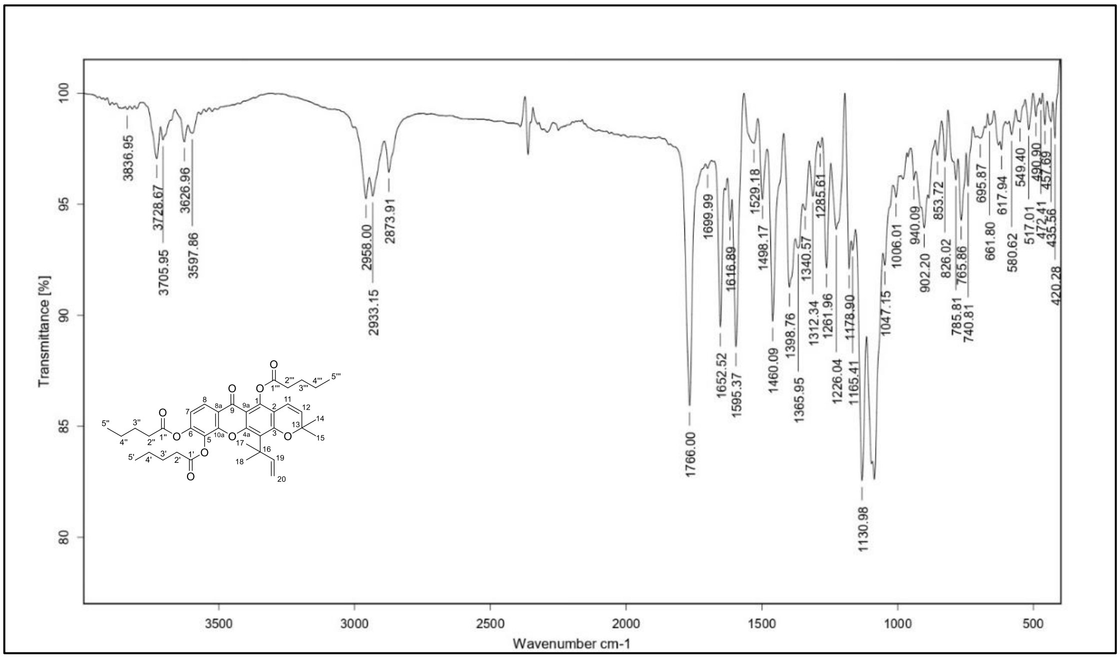

Figure S36 IR spectrum of 1,5,6-tripentanoyloxymacluraxanthone (**2b**)

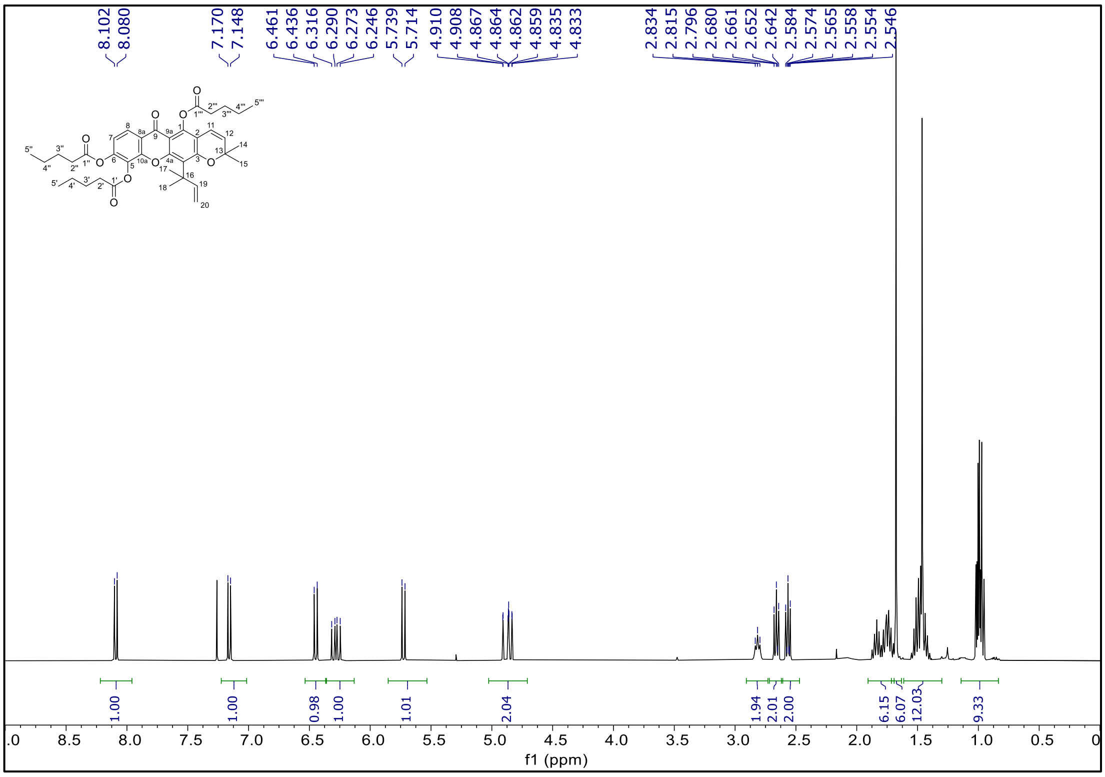

Figure S37 ^{1}H NMR spectrum of 1,5,6-tripentanoyloxymacluraxanthone (**2b**) in $CDCl_3$

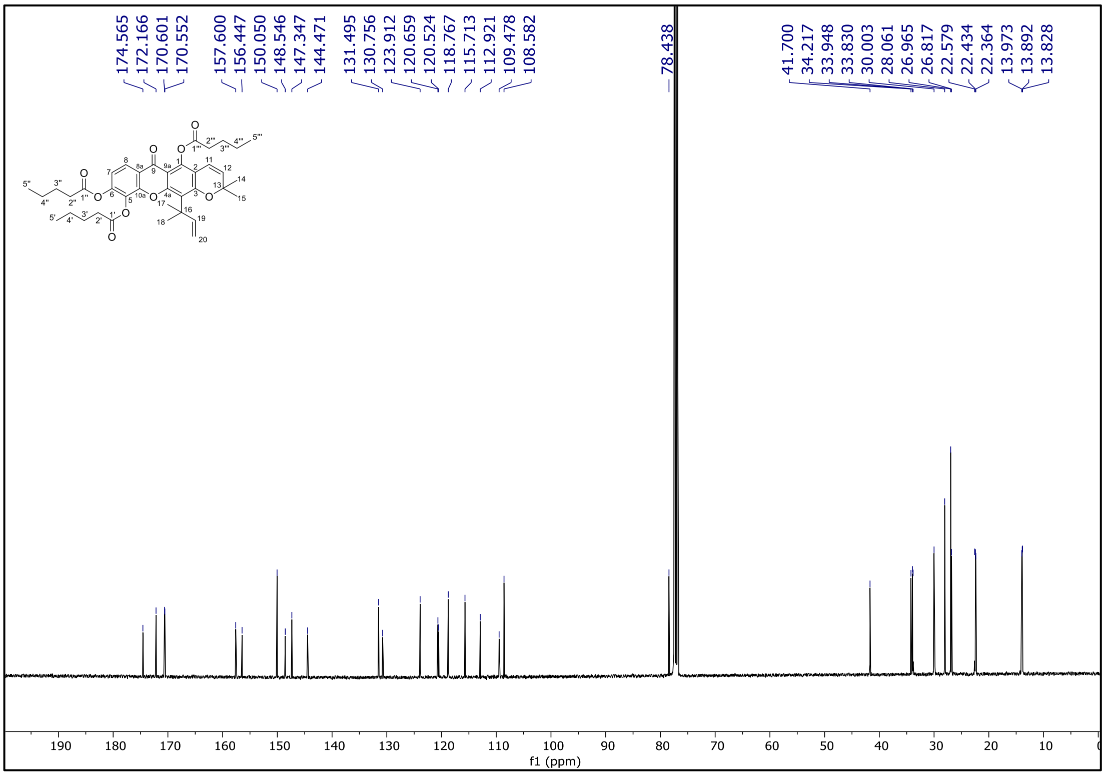

Figure S38 ^{13}C NMR spectrum of 1,5,6-tripentanoyloxymacluraxanthone (**2b**) in $CDCl_3$

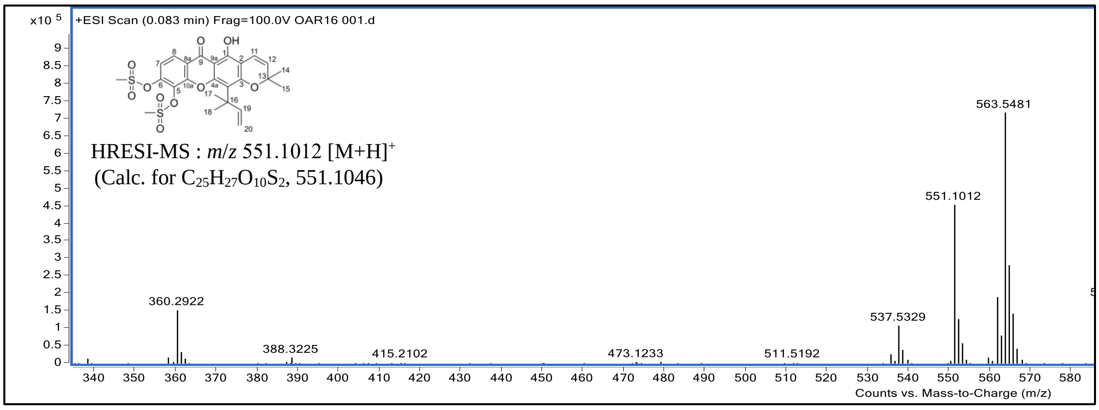

Figure S39 MS spectrum of 5,6-dimesyloxymacluraxanthone (**2c**)

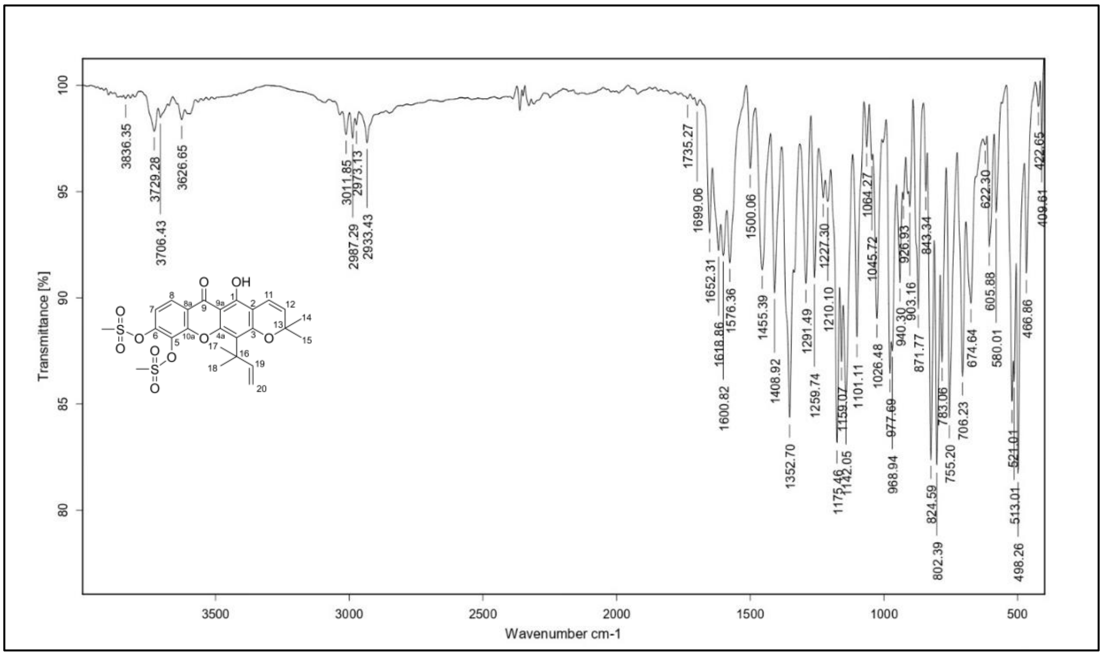

Figure S40 IR spectrum of 5,6-dimesyloxymacluraxanthone (**2c**)

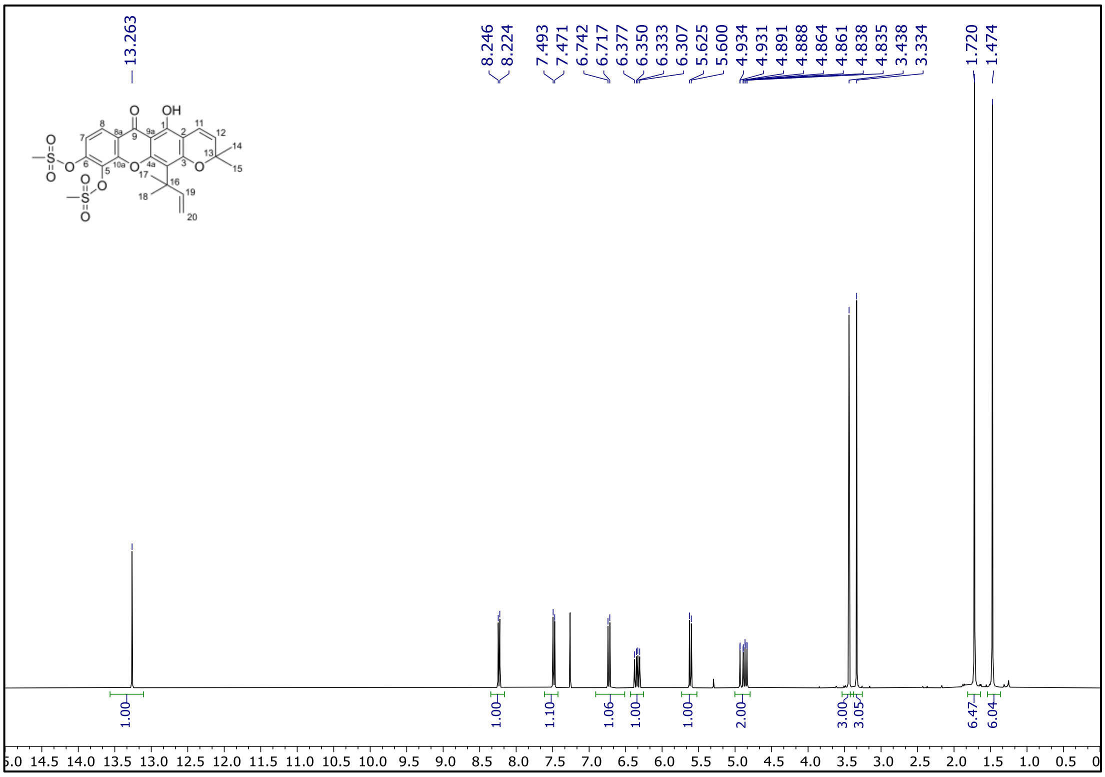

Figure S41 ^{1}H NMR spectrum of 5,6-dimesyloxymacluraxanthone (**2c**) in $CDCl_3$

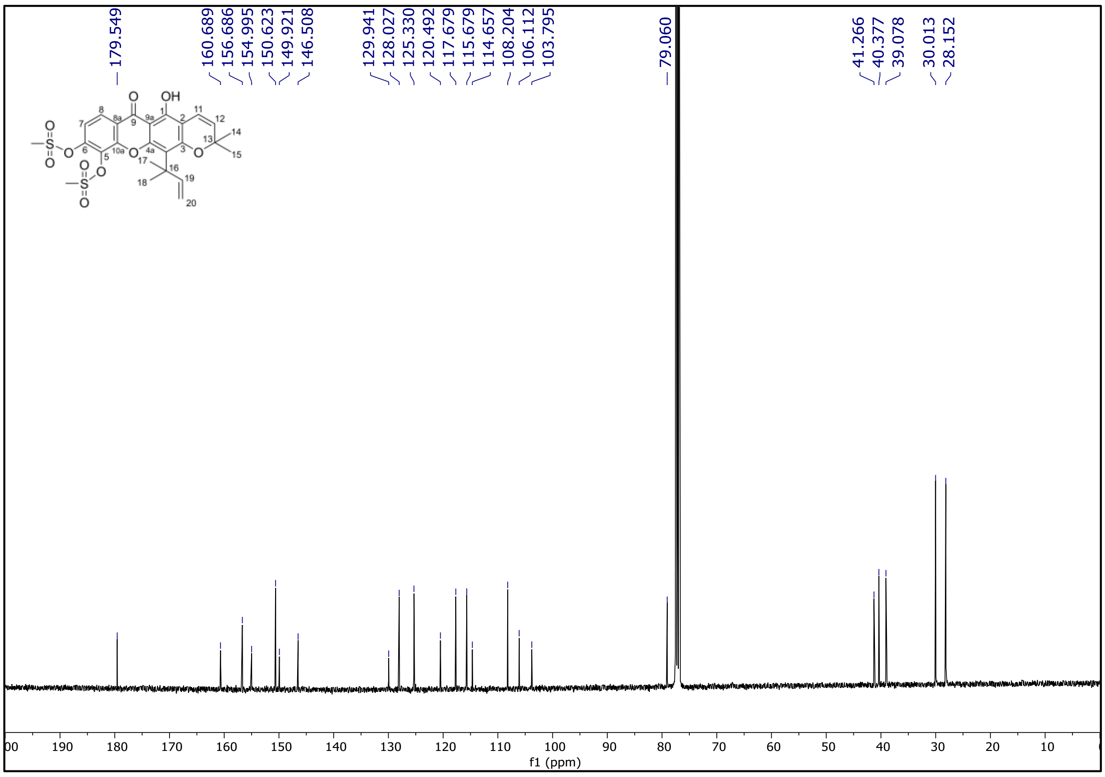

Figure S42 ^{13}C NMR spectrum of 5,6-dimesyloxymacluraxanthone (**2c**) in $CDCl_3$

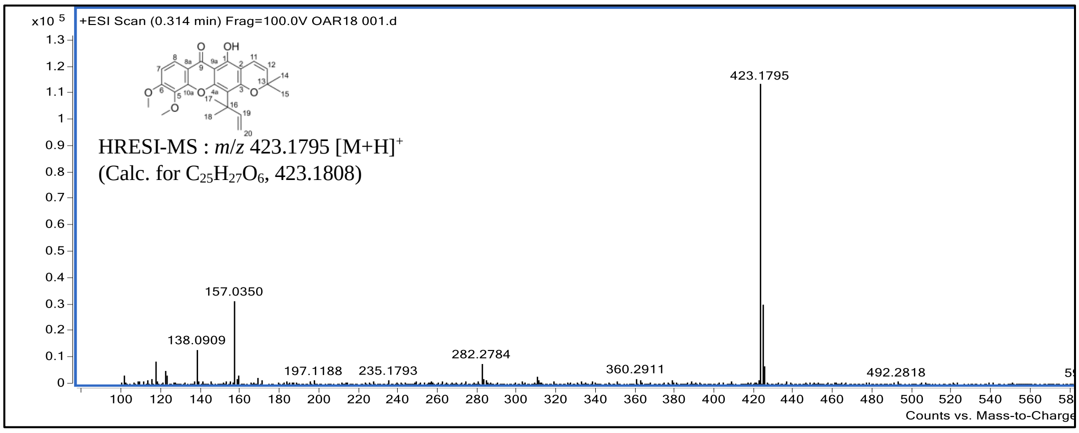

Figure S43 MS spectrum of 5,6-dimethoxymacluraxanthone (**2d**)

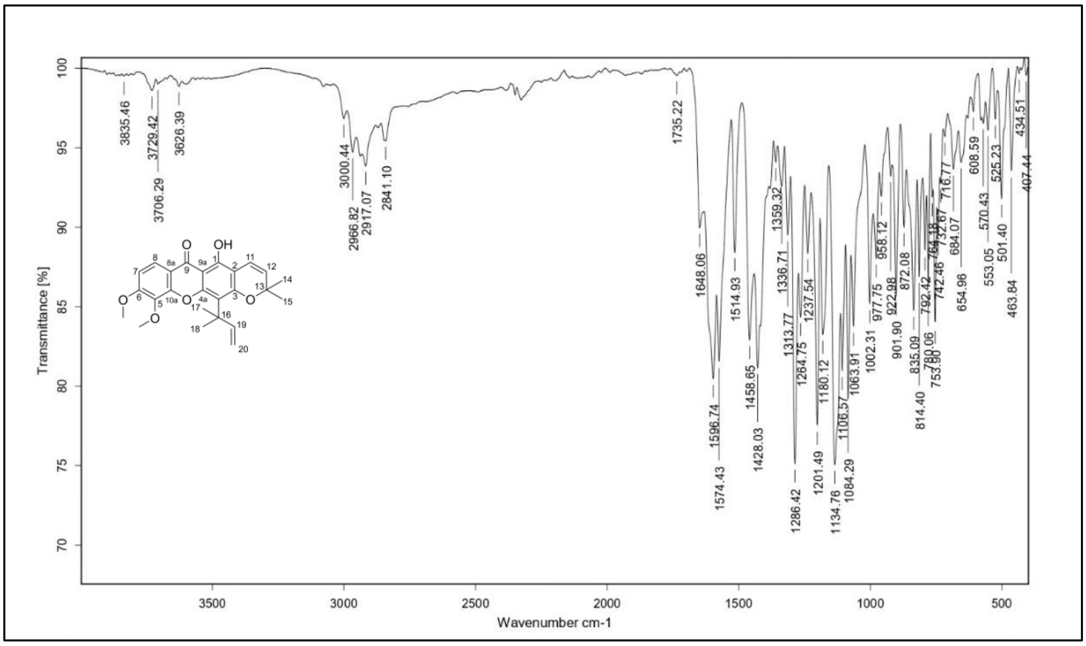

Figure S44 IR spectrum of 5,6-dimethoxymacluraxanthone (**2d**)

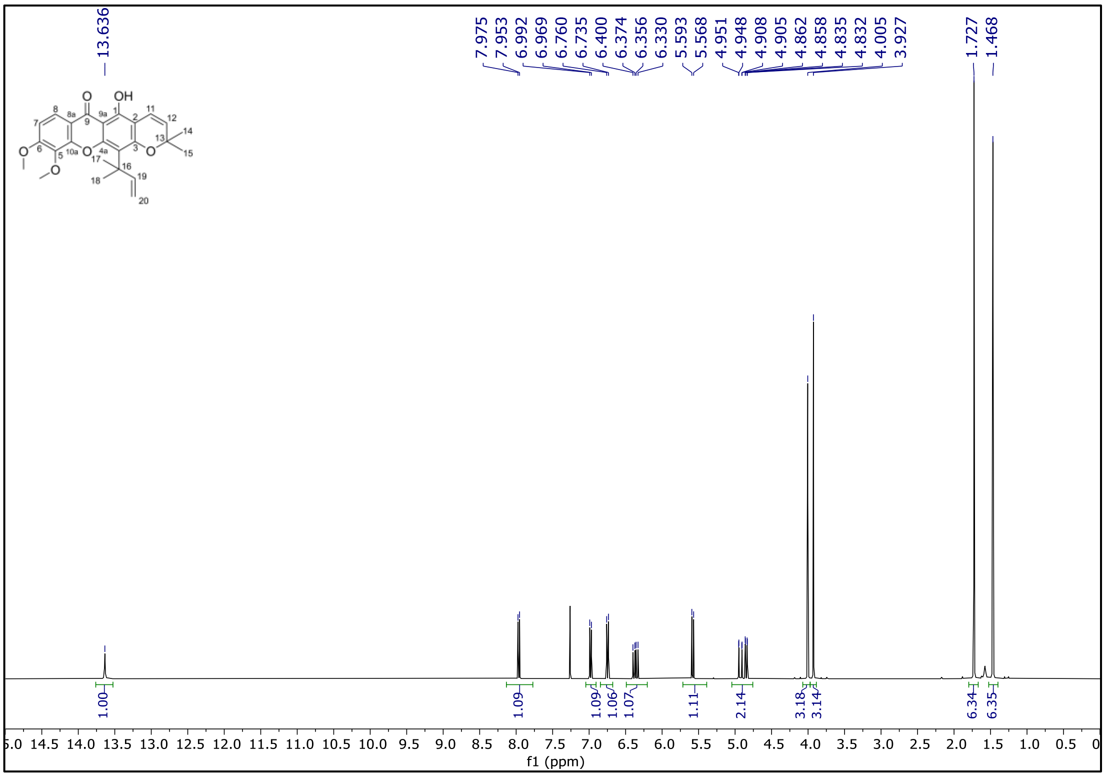

Figure S45 ^{1}H NMR spectrum of 5,6-dimethoxymacluraxanthone (**2d**) in $CDCl_3$

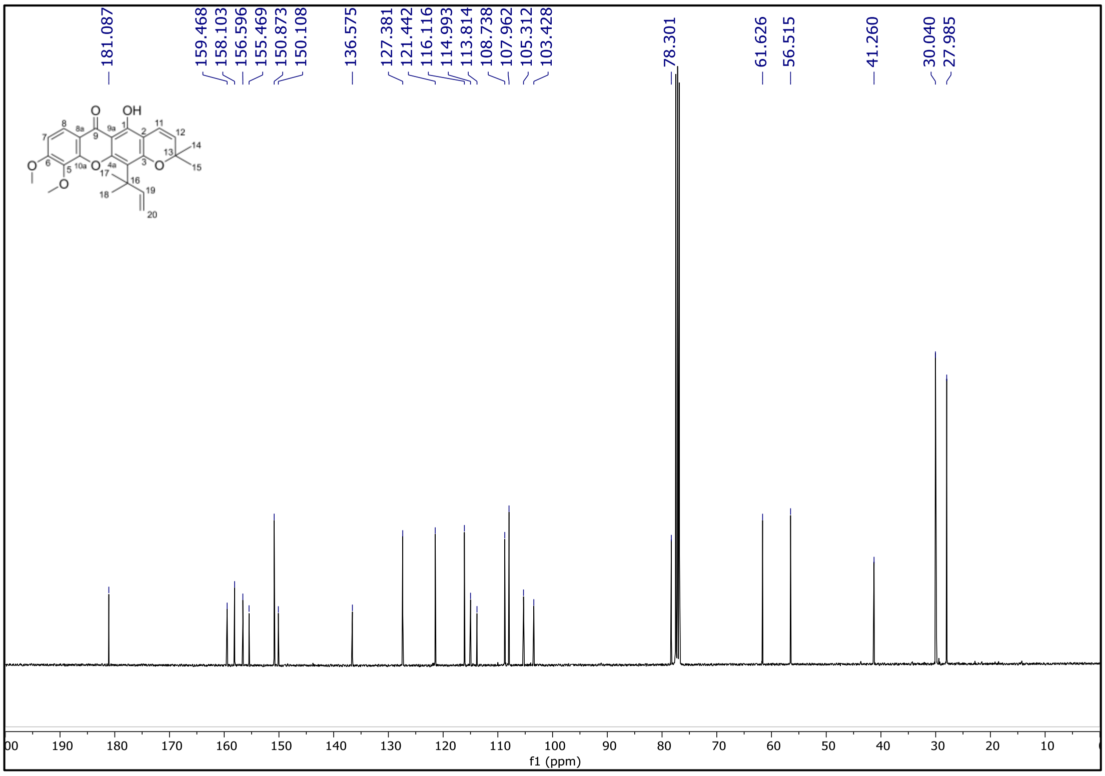

Figure S46 ^{13}C NMR spectrum of 5,6-dimethoxymacluraxanthone (**2d**) in $CDCl_3$

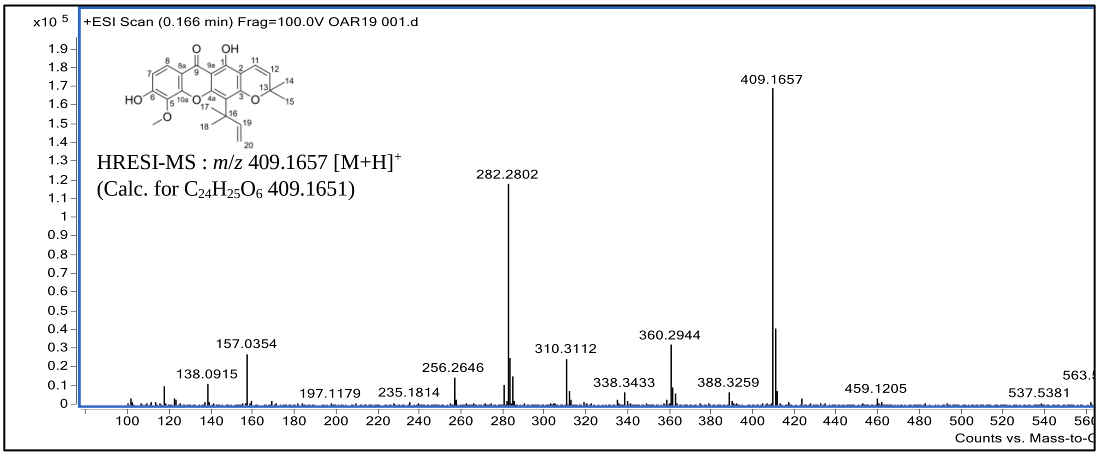

Figure S47 MS spectrum of 5-methoxymacluraxanthone (**2e**)

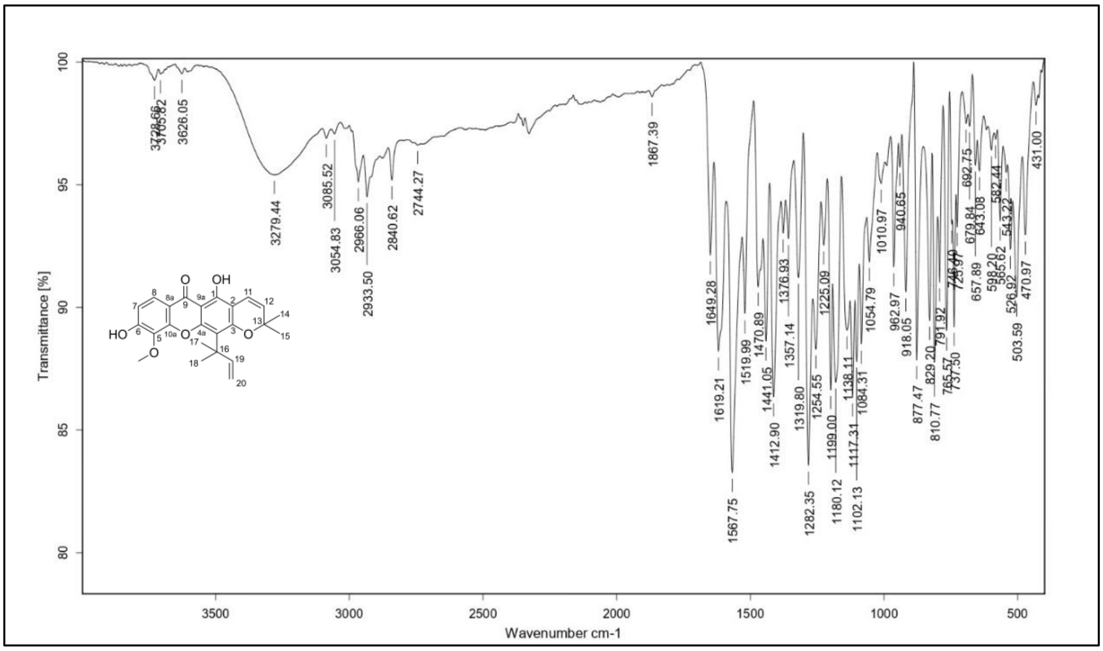

Figure S48 IR spectrum of 5-methoxymacluraxanthone (**2e**)

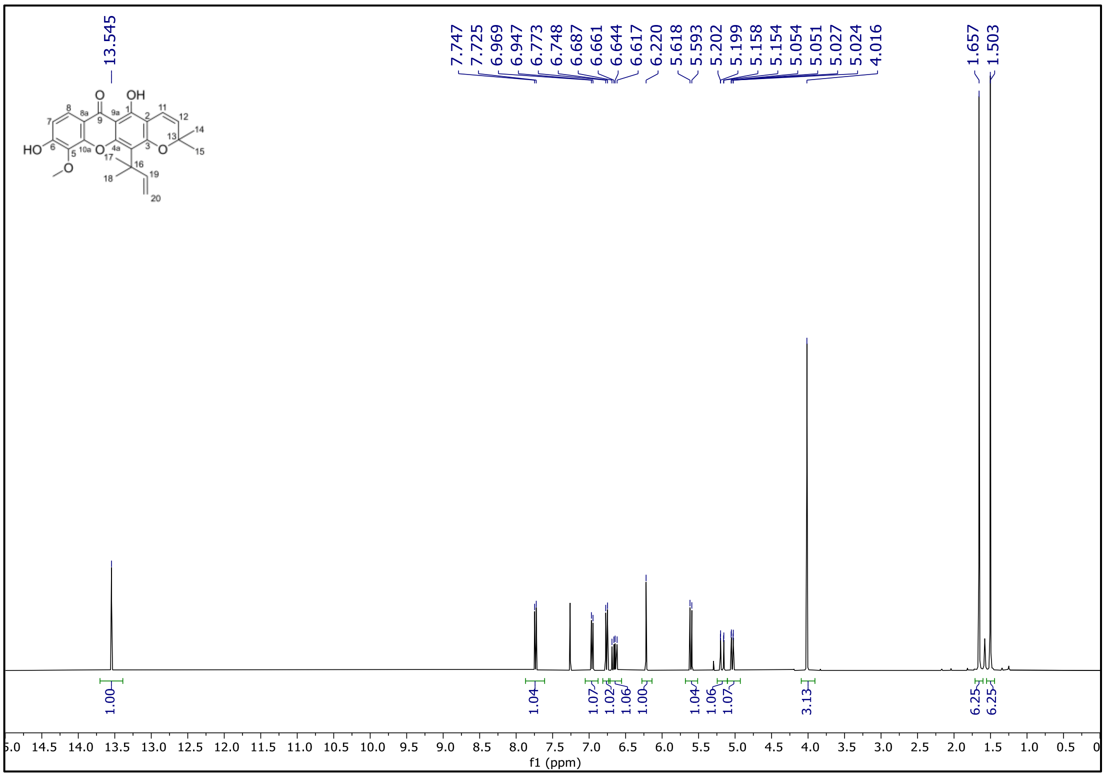

Figure S49 ^{1}H NMR spectrum of 5-methoxymacluraxanthone (**2e**) in $CDCl_3$

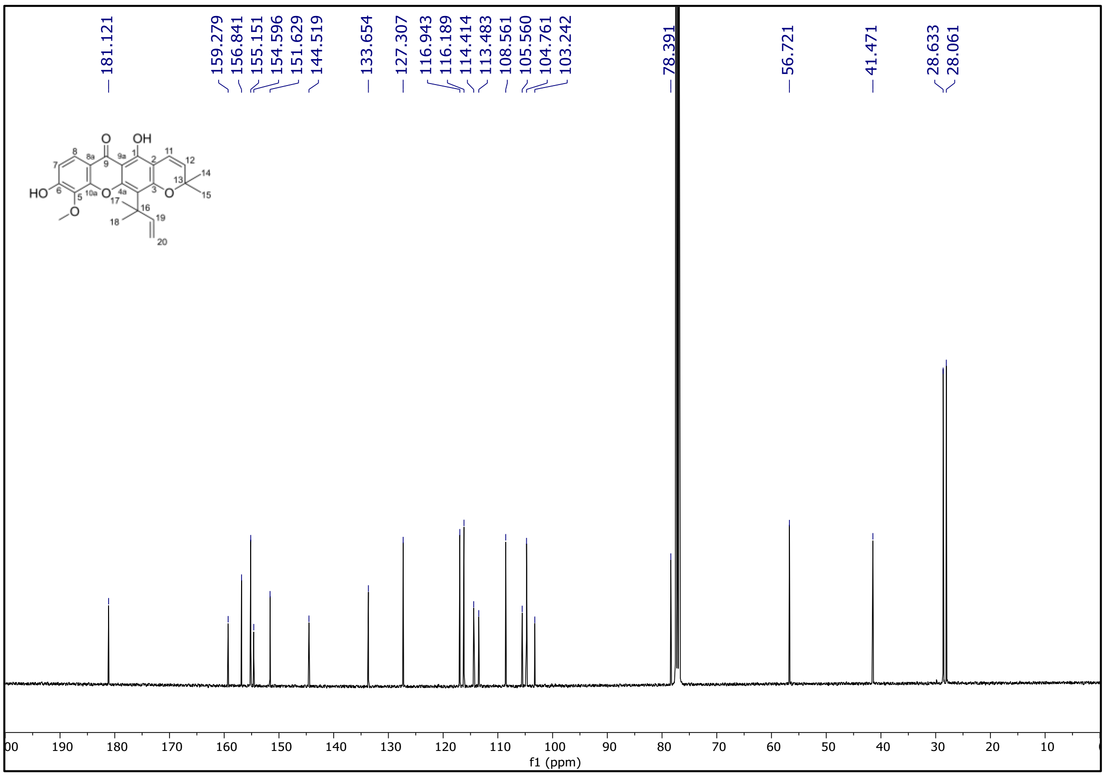

Figure S50 ^{13}C NMR spectrum of 5-methoxymacluraxanthone (**2e**) in $CDCl_3$

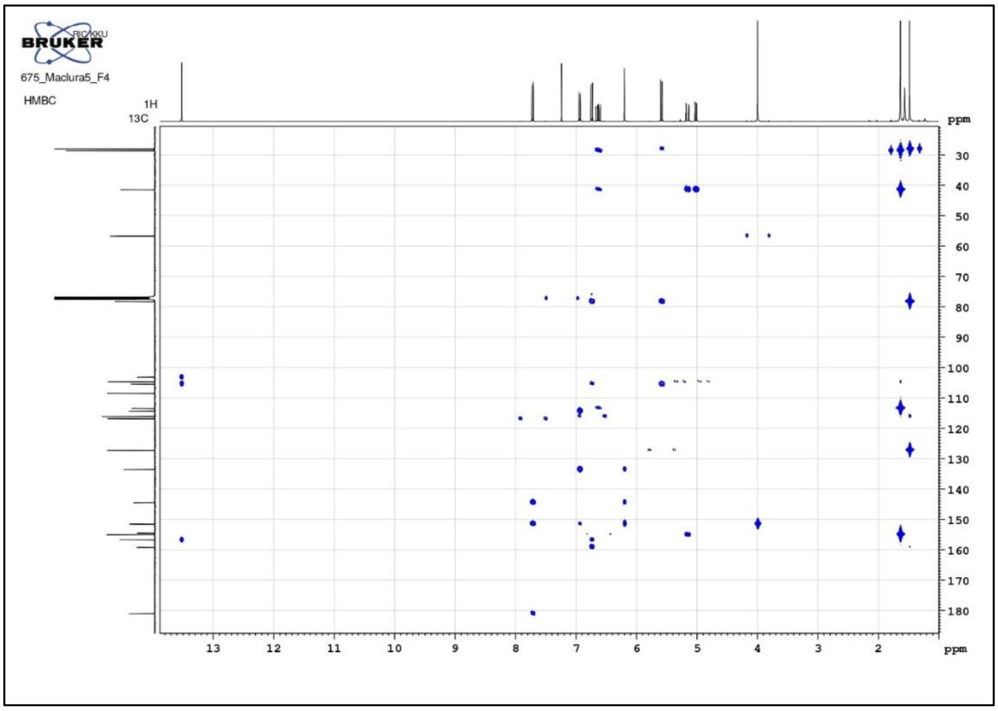

Figure S51 HMBC spectrum of 5-methoxymacluraxanthone (**2e**)

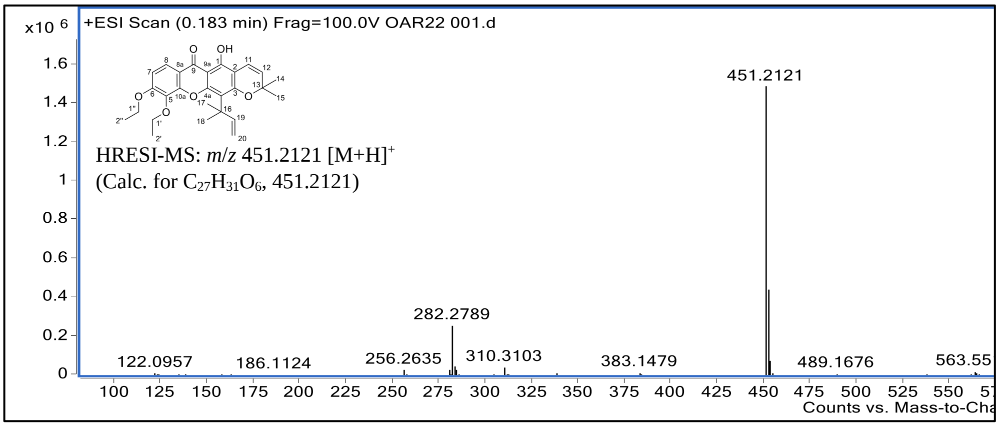

Figure S52 MS spectrum of 5,6-diethoxymacluraxanthone (**2f**)

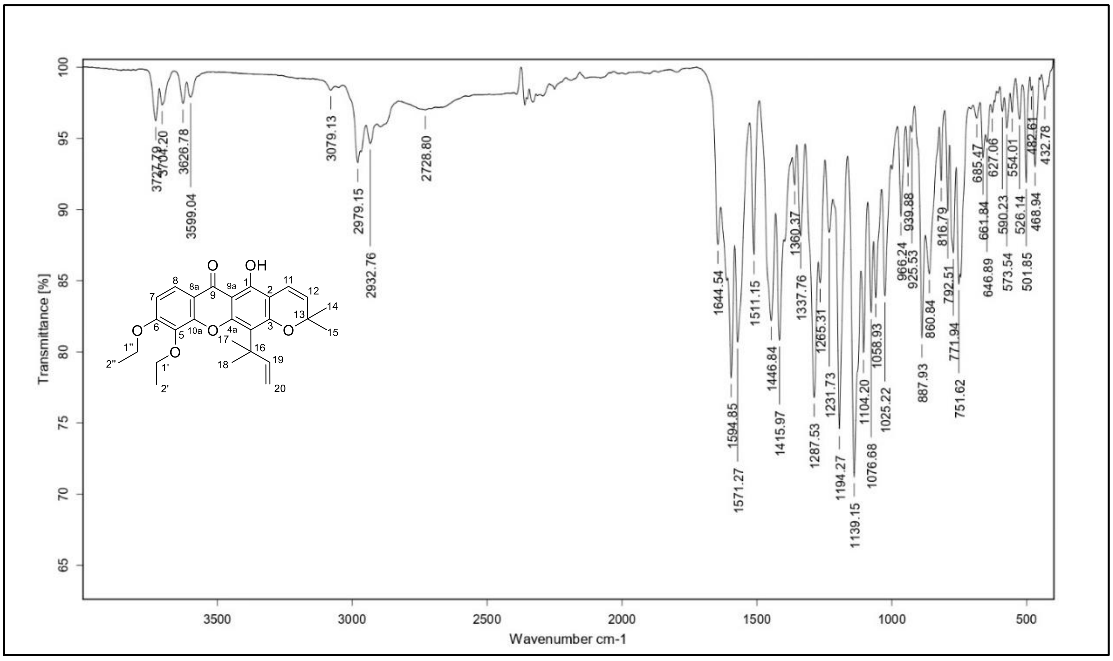

Figure S53 IR spectrum of 5,6-diethoxymacluraxanthone (**2f**)

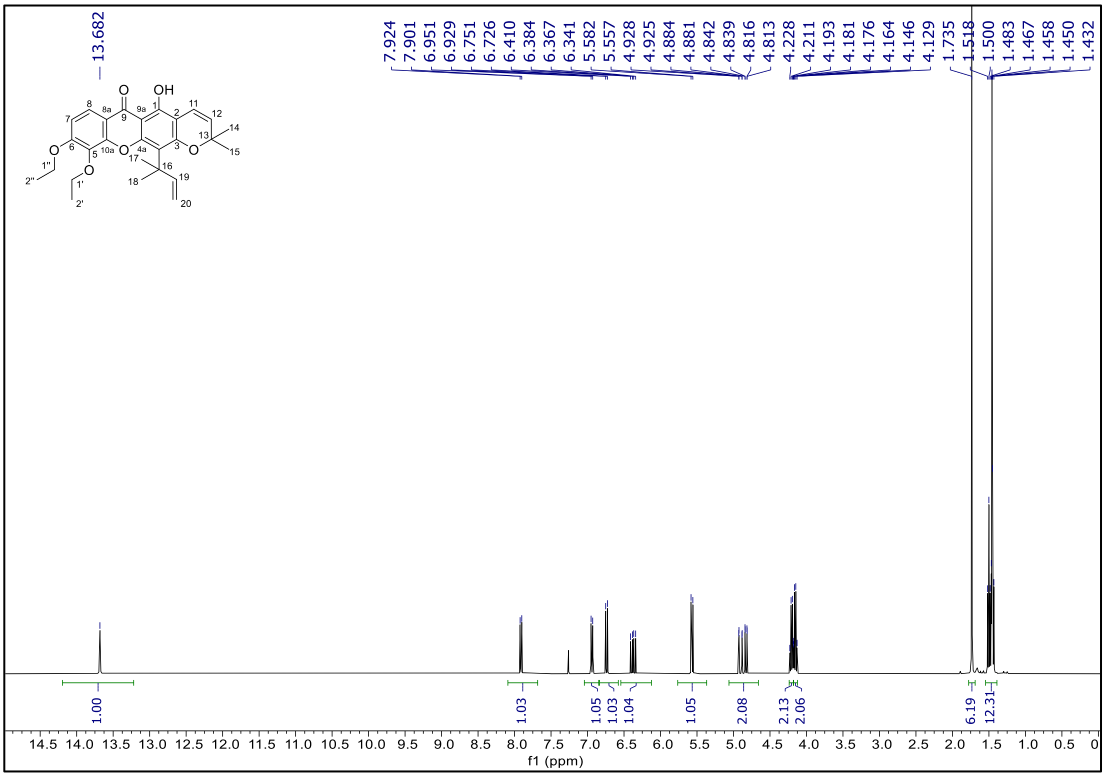

Figure S54 ^{1}H NMR spectrum of 5,6-diethoxymacluraxanthone (**2f**) in $CDCl_3$

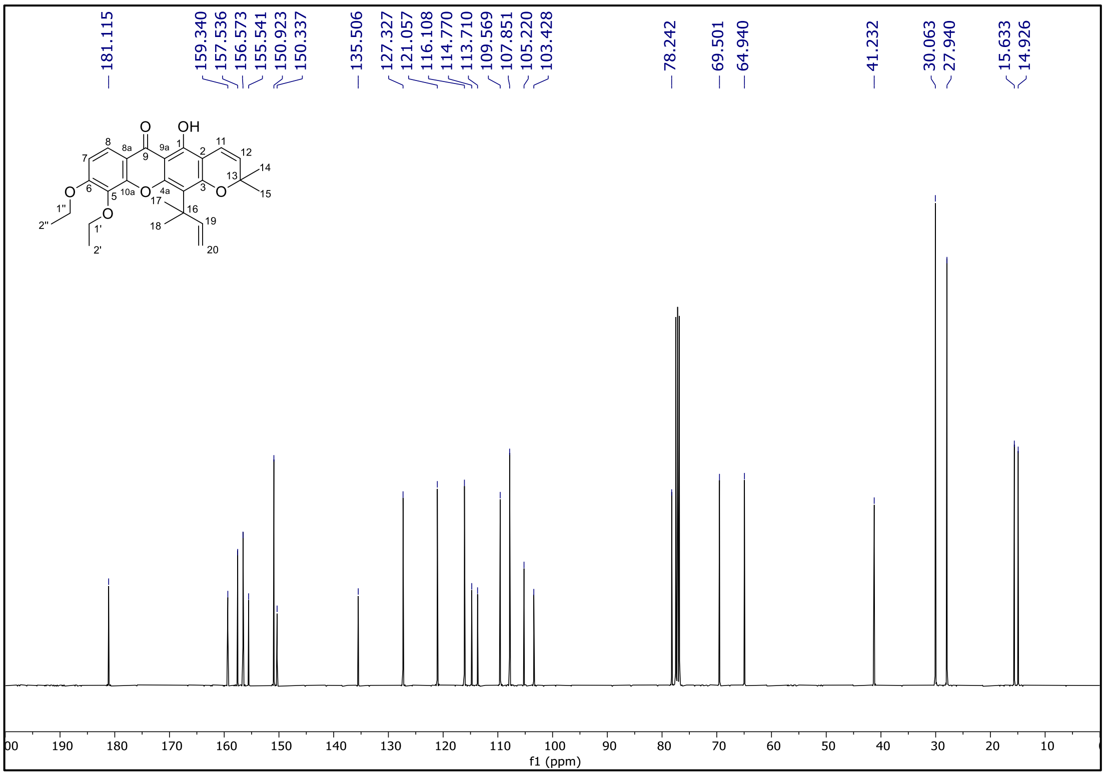

Figure S55 ^{13}C NMR spectrum of 5,6-diethoxymacluraxanthone (**2f**) in $CDCl_3$

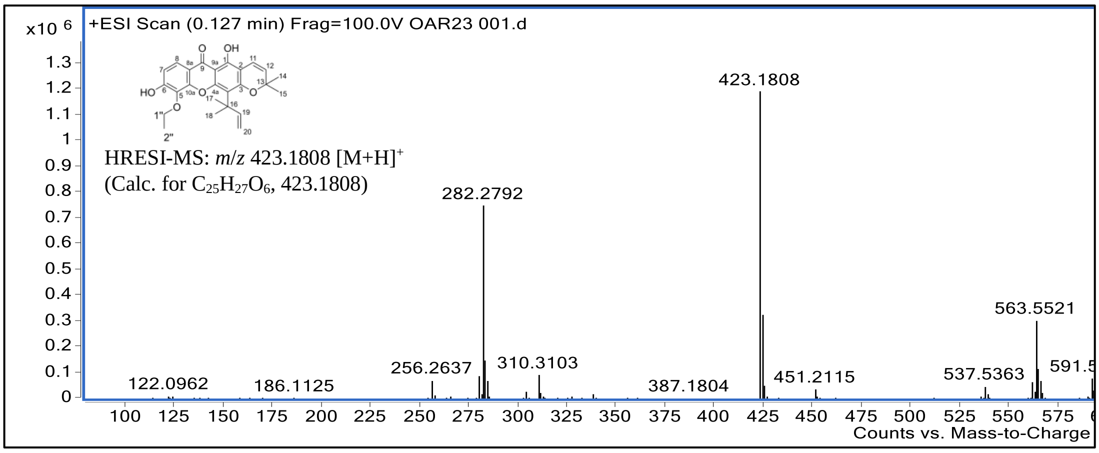

Figure S56 MS spectrum of 5-ethoxymacluraxanthone (**2g**)

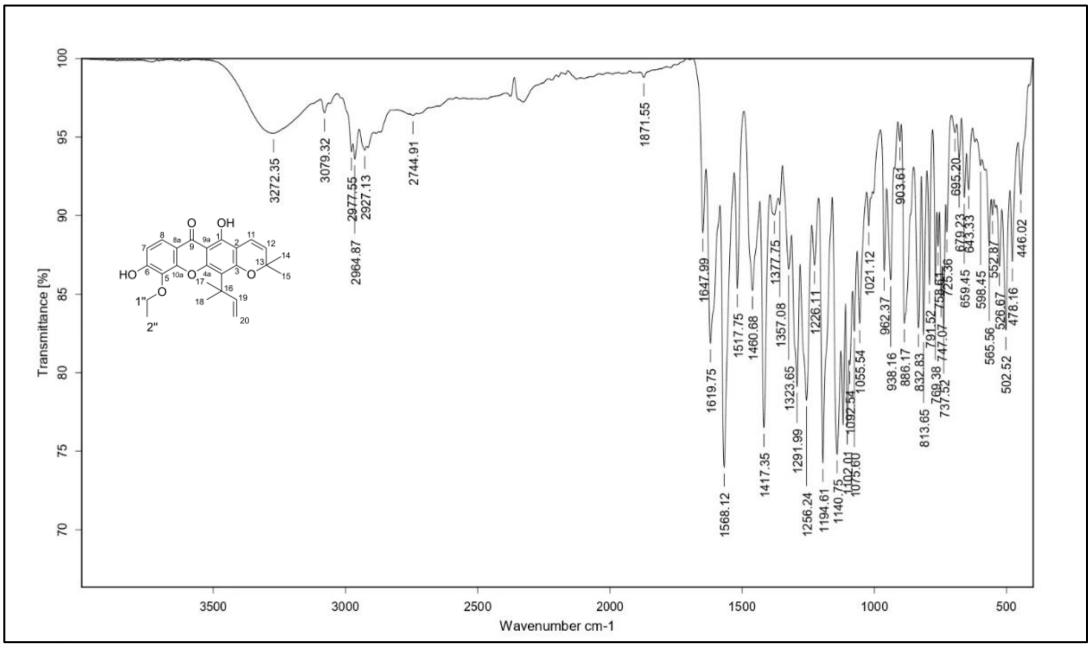

Figure S57 IR spectrum of 5-ethoxymacluraxanthone (**2g**)

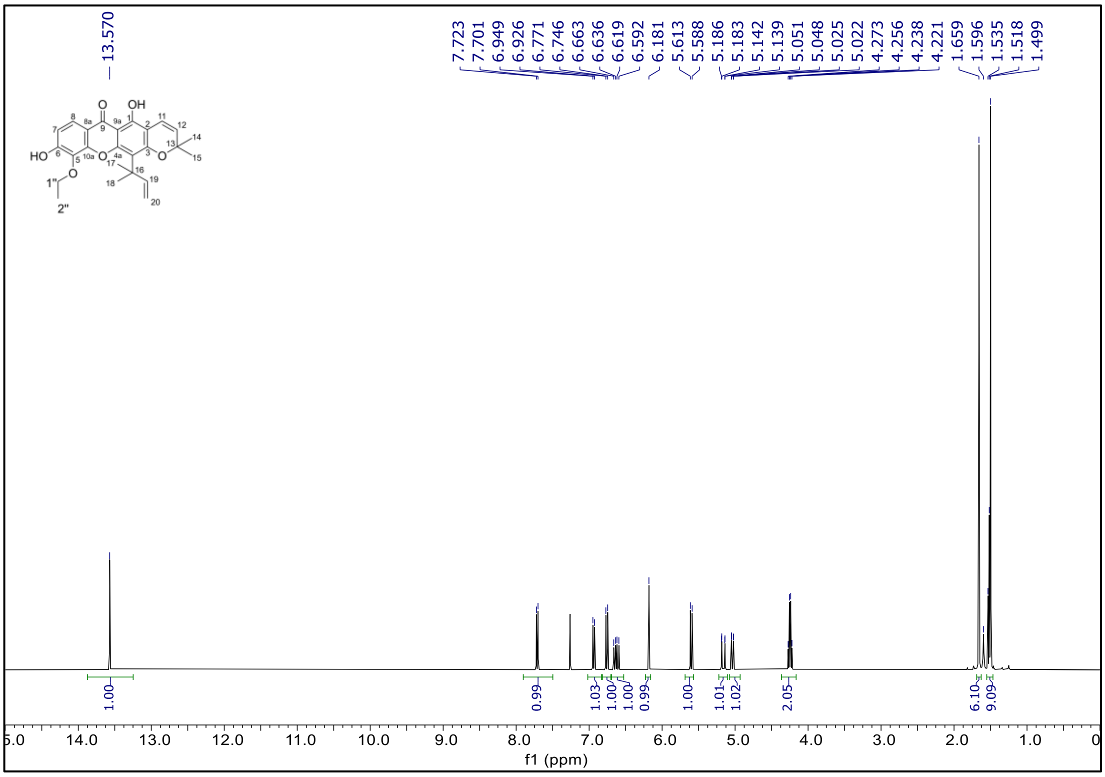

Figure S58 ^{1}H NMR spectrum of 5-ethoxymacluraxanthone (**2g**) in $CDCl_3$

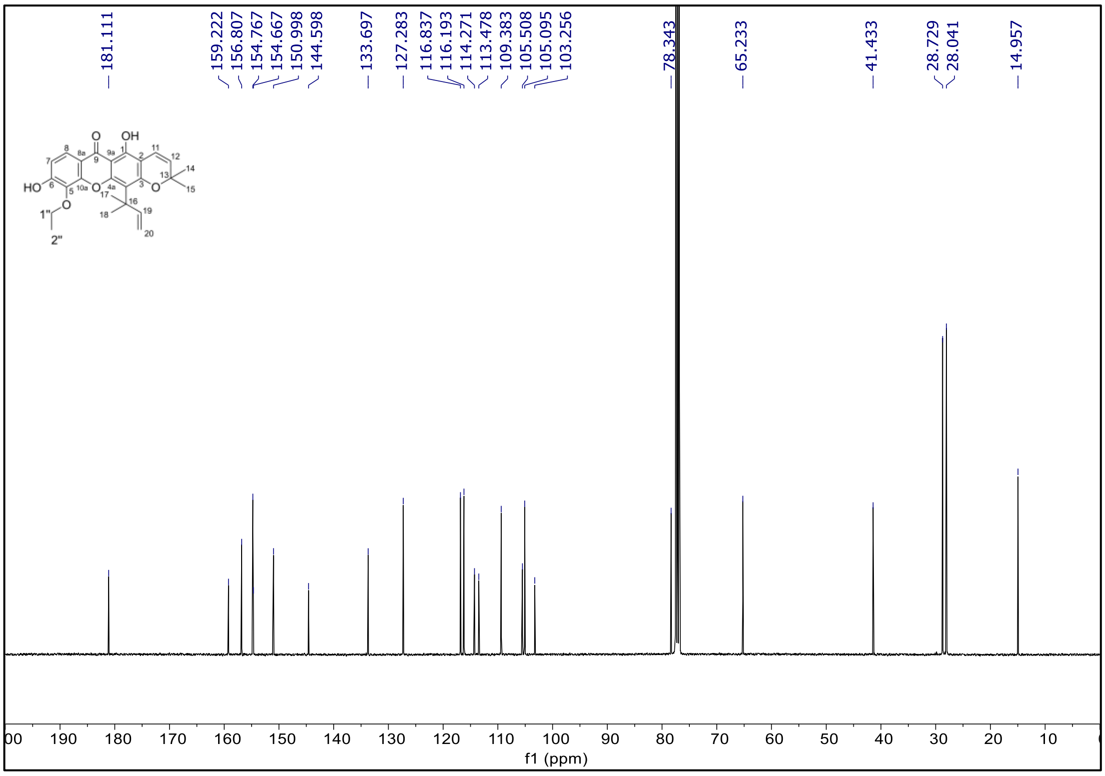

Figure S59 ^{13}C NMR spectrum of 5-ethoxymacluraxanthone (**2g**) in $CDCl_3$

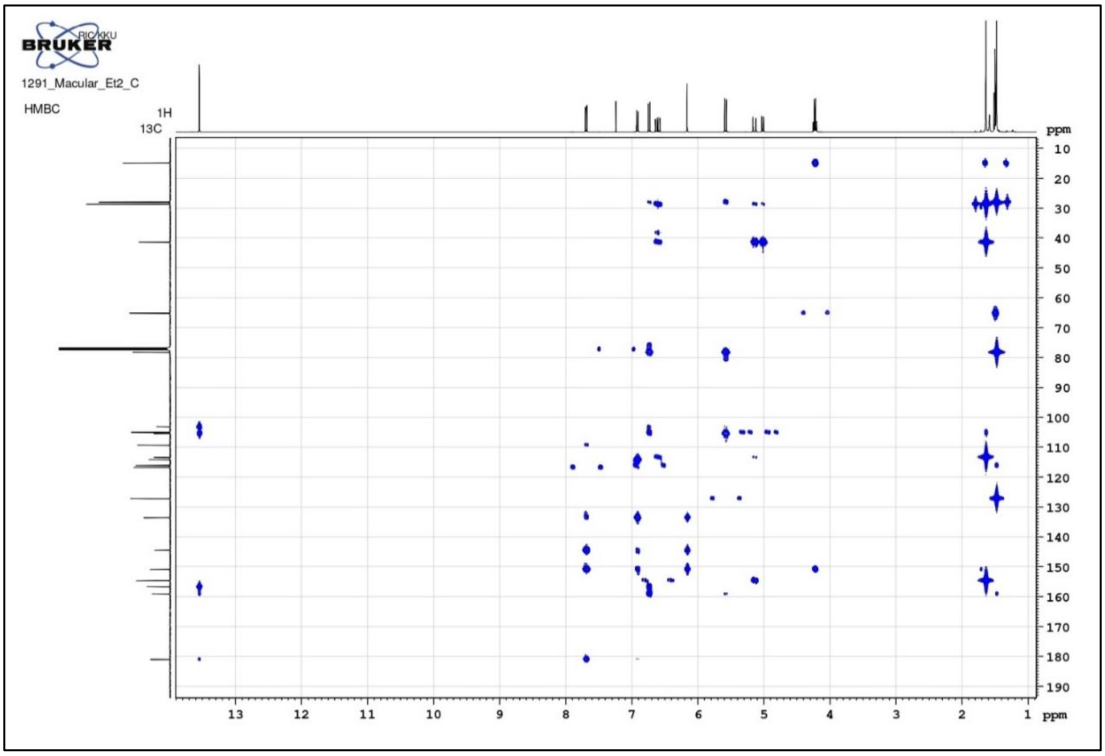

Figure S60 HMBC NMR spectrum of 5-ethoxymacluraxanthone (**2g**)
